# Stepwise Synthesis of Tetrabenzotriazaporphyrins (TBTAPs) and Their Open 2‐ and 3‐Ring Fragments

**DOI:** 10.1002/chem.71357

**Published:** 2026-07-06

**Authors:** Jacob Gretton, Ala Alturk, Sonia Remiro‐Buenamañana, Alejandro Díaz‐Moscoso, Amirah A. Aldawsari, Maha S. Albayaa, Isabelle Chambrier, David L. Hughes, Simon J. Coles, Graham J. Tizzard, Andrew N. Cammidge

**Affiliations:** ^1^ School of Chemistry, Pharmacy and Pharmacology University of East Anglia Norwich UK; ^2^ Instituto De Investigaciones Químicas (IIQ‐CICIC) Seville Spain; ^3^ UK National Crystallography Service, School of Chemistry University of Southampton Southampton UK

**Keywords:** helicene, hybrids, phthalocyanines, porphyrins, synthesis

## Abstract

The intermediate structures between phthalocyanines and porphyrins (so‐called hybrid structures) are intriguing materials that offer the potential to combine features of each parent, and also offer tunability of properties through modification and further functionalization. We report here the stepwise synthesis of tetrabenzotriazaporphyrins(TBTAPs) bearing different core and meso‐aryl substituents. The results are unexpected; our previous results showed that the reaction between aminoisoindoline initiator (“A”) and a phthalonitrile (“B”) led preferentially to 2:2, ABBA hybrids, presumed to be via reaction between 2x “AB” intermediates. Here we report the synthesis of discrete “AB” fragments and their subsequent reaction chemistry. Reaction of the AB intermediates under typical macrocyclization conditions fails to produce more than a trace of the expected ABBA TBTAP hybrid. Rather, the reactions yield the 3:1 ABBB TBTAPs predominantly, alongside an open ABA trimer (a tripyrrin analogue). The protocol provides a useful alternative synthesis of TBTAPs that minimizes the formation of phthalocyanine side‐product. ABA trimer synthesis was separately optimized to produce a short series. The materials show broad absorption across the visible range and assemble into a preferred helical, coiled arrangement as revealed by crystallography.

## Graphical abstract

Unexpected reaction pathways to porphyrin‐phthalocyanine hybrids are uncovered by the synthesis of “half‐macrocycle” fragments and subsequent macrocyclization. Rather than the expected 2:2 “ABBA” product, the 3:1 “ABBB” hybrid is isolated as the only macrocyclic product. Open trimers, isolated as side products, show broad absorption spectra and intriguing, coiled conformations.

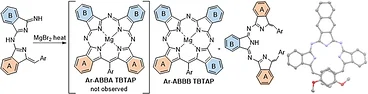

## 1 Introduction

Porphyrinoid macrocycles are ubiquitous in both nature and our everyday lives. The parent structures, porphyrin and phthalocyanine, have been extensively studied and their chemistry and application have been comprehensively reviewed [1, 2]. Linear fragments of porphyrins have separately received significant attention, arguably most notably in relation to the synthetic dipyrromethene compounds and their boron complexes (“BODIPYs”) because of their favorable absorption and emission properties in the visible range, and due to the ability to tune properties by synthetic manipulation [3, 4, 5, 6]. Hybrid structures that share features of both porphyrins (C‐bridges between pyrrolic units) and phthalocyanines (N‐bridges, benzo‐fusion on pyrrolic units) have been known for a century [7]. Such hybrids offer much potential because of the ability to combine properties of both parents (a different approach to tuning) and the inherent additional versatility that is presented in unsymmetrical structures. However, their chemistry is much less well developed than that of the parent macrocycles because synthesis has been challenging and often inefficient. It is worth noting that hybrid structures are also known for the linear fragments, and they also show tunability of properties. The syntheses of these smaller systems are further advanced than for the porphyrinoid hybrids themselves; for example, there are now many examples of aza‐ and benzo‐fused BODIPY analogues (Figure 1) [8].

**FIGURE 1:**
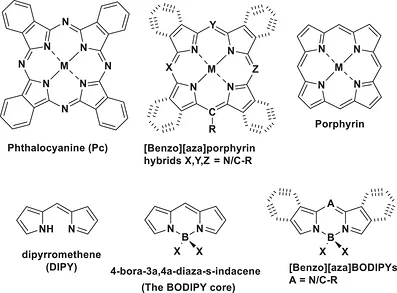
Core structures of porphyrin, phthalocyanine, DIPY, and BODIPY, plus their hybrids.

Our previous work has expanded the chemistry of the porphyrin–phthalocyanine hybrids, initially through detailed investigation of the use of organometallic nucleophiles reacting with phthalonitrile derivatives [9, 10, 11] (reactions related to the original hybrid syntheses reported in the 1930's [12, 13]). Observations of likely intermediates in these reaction pathways led to the development of a novel synthesis of *meso*‐aryl‐triazatetrabenzoporphyrins (TBTAPs, e.g., **2**) employing aminoisoindoline precursors **1** (Scheme 1) [14, 15]. The related subPhthalocyanine hybrids **3** were prepared when a boron template was employed in place of magnesium [16]. In both cases, the assumed mechanism was one where the aminoisoindoline initiated sequential addition of phthalonitrile units, followed by macrocyclization.

**SCHEME 1:**
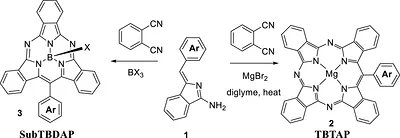
Synthesis of TBTAPs **2** and subTBDAPs **3** from aminoisoindoline precursors **1**.

Subsequent extension of the synthesis strategy was investigated in order to demonstrate scope and to produce derivatives that incorporated, in a controlled manner, additional functionality around the core itself. This was achieved in a complementary fashion by modification of the benzo‐fragment of the aminoisoindoline (unit “A”, to incorporate this single substituted benzo fragment next to the *meso*‐carbon), and/or modification of the phthalonitrile co‐reactant (unit “B”, to be incorporated as the other three benzo‐units around the macrocycle). The expected derivatives were therefore “ABBB” TBTAPs. Remarkably, however, the outcome was a mixture of the expected ABBB systems (minor product, even with slow addition of “A” to excess of “B”) and ABBA TBTAPs (major product) [17]. The reaction outcome implies operation of alternative competing mechanisms, and dominance of the 2:2 ABBA hybrid pointed strongly to a sequence involving condensation between 2 “AB” intermediates. The Ar‐ABBA system presents a potentially very useful architecture, with high symmetry and three distinct regions for tuning electronic and other properties (“A” and “B” regions of the core, and the largely decoupled *meso*‐aryl unit).

## 2 Results and Discussion

### 2.1 Synthesis and Isolation of “AB” Intermediates

The mechanism shown in Scheme 2 was considered the most likely origin of the ABBA TBTAP hybrids. To test the hypothesis and to potentially fully control the formation of a single hybrid, access to the discrete “AB” intermediates was required. Intercepting related intermediates in phthalocyanine chemistry has been attempted with some limited success [18, 19, 20, 21, 22, 23], but it is often challenging to find reaction conditions that initiate oligomer growth without over‐reaction to the stable macrocycle. Phthalonitriles themselves are generally found to have low reactivity—a feature we exploited to produce TBTAP hybrids efficiently [14, 15]. For the current investigation, however, we sought to deliberately activate phthalonitriles such that they would react selectively with aminoisoindolines like **1**, and under mild conditions. We focused on the reaction products produced when phthalonitrile derivatives are reacted with (sodium) alkoxides at low or ambient temperatures [24, 25]. Several examples of the resulting cyclic products have been made, characterized, and indeed employed in phthalocyanine chemistry [26, 27]. With simple, unhindered alcohols such as methanol, products from double addition are isolable [24, 25], whereas it is known that more hindered alcohols lead to isolation of the single‐addition adducts [24, 25]. Under solution‐phase reaction conditions, it is expected that the systems are in equilibrium, with the mono‐alkoxy adducts more reactive toward nucleophiles (Scheme 3). We investigated several examples that qualitatively supported this pattern. In particular, we found that spirocyclic adducts (formed from diols) were particularly stable and easy to isolate (see Supporting Information). We found overall that the simple methanol adduct offered the best balance between ease of isolation (driven by solubility, with the adduct easily isolated in pure form, by simple filtration from the reaction mixture) and reactivity. Synthesis of “AB” intermediates initially employed aminoisoindoline **1a**. Reaction with the preformed dimethoxy phthalonitrile adduct **6a** proceeded smoothly, and again it was convenient to exploit solubility differences to drive the overall equilibration and achieve simple isolation of the AB intermediates, initially by filtration. After further optimization, it was found that in many cases the AB intermediates could be prepared in a single operation by direct reaction between aminoisoindolines and phthalonitrile in methanol with catalytic sodium methoxide, presumably via transient phthalonitrile–methanol adduct **5** (R = Me) (Scheme 3).

**SCHEME 2:**
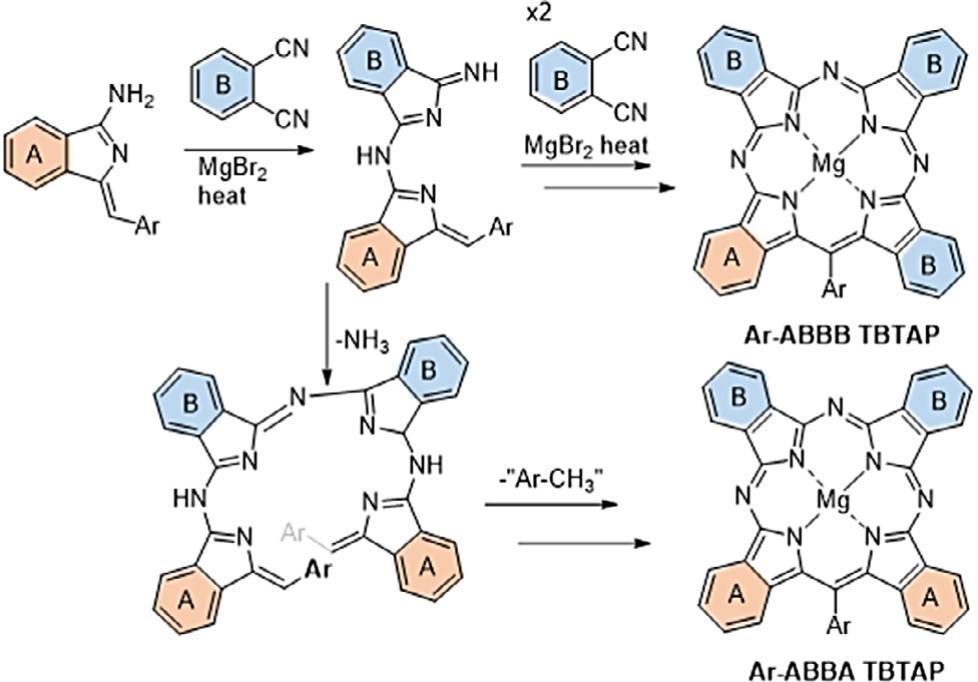
Initially proposed mechanistic sequences that lead to ABBB (3:1) and ABBA (2:2) TBTAP hybrids [17].

**SCHEME 3:**
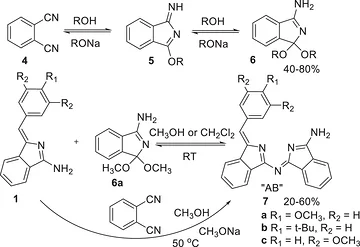
Sequential and direct synthesis of “AB” intermediates.

AB intermediates **7a–c** bear identical (parent) benzo substituents on both A and B units, so would not deliver differentially substituted TBTAP hybrids. Nevertheless, AB intermediates **7a–c** were subjected to macrocycle formation conditions in order to ensure that TBTAP formation occurred in accordance with the proposed sequence (Scheme 2, bottom). TBTAP formation occurred smoothly when **7a–c** were heated (initially in diglyme at reflux) in the presence of MgBr_2_ as a template. In fact, these direct reactions were found to have some advantages over our initially developed protocol that employed aminoisoindoline and phthalonitrile. Yields were comparable (up to ∼40%), but the process is operationally simplified, using just a single reactant plus template. Furthermore, macrocycle formation could be achieved at lower temperatures (refluxing xylene or toluene), and the combination of lower reaction temperatures and no additional phthalonitrile meant that formation of phthalocyanine side product was reduced to just trace quantities, making separation more straightforward (Scheme 4).

**SCHEME 4:**
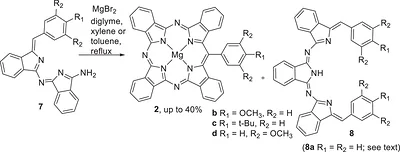
Direct synthesis of meso‐aryl TBTAPs **2** from pre‐formed “AB dimers”, plus the observed “ABA trimer” side products **8**.

Scheme 4 also shows the formation of an open trimeric product formed from 2 aminoisoindoline units and a central phthalonitrile. We have observed structure **8a** as a trace component in the early stages of reactions between aminoisolindoline **1** (R_1_ = R_2_ = H) and phthalonitrile templated by boron in the synthesis of subPhthalocyanine hybrids [16] (Scheme 1, left), but this is the first observation of their formation in significant quantities. Their optimized synthesis and characterization are described later.

The successful transformation of AB intermediates **7** justified the synthesis of corresponding derivatives bearing different “A” and “B” units in order to interrogate the reaction pathway. A short series of derivatives bearing substituents on either the benzo unit of the aminoisoindoline portion or the phthalonitrile‐derived portion was selected, and in general terms, their synthesis followed the protocols developed for the parent series **7**. AB dimer intermediates were prepared by up to three variations on the same general strategy, and the choice of protocol was determined by consideration of solubility (and therefore equilibrium in the synthesis steps and ease of isolation). Substituted aminoisoindoline precursors were prepared as described in our earlier studies [17]. Substituted phthalonitriles were converted to their corresponding dimethoxyisoindolinamine intermediates by treatment with sodium methoxide in the minimum volume of methanol, although the synthesis protocol was refined for these derivatives. Scheme 5 illustrates the sequences for the tetramethyltetralin derivative, and the structures of two further AB dimers that were targeted to investigate TBTAP synthesis.

**SCHEME 5:**
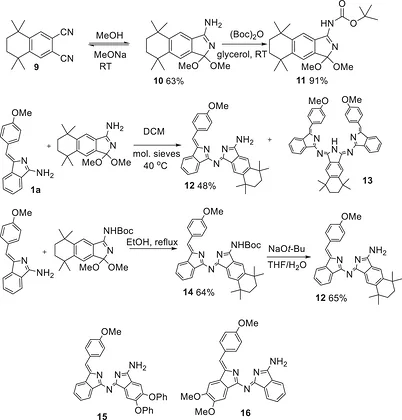
Synthesis of differentially substituted AB dimers.

Treatment of substituted phthalonitriles like **9** with sodium methoxide/methanol led again to the formation of the required intermediate **10**. However, as mentioned previously, the reaction is reversible, and its overall efficiency is controlled by the selective precipitation of the product. In the case of substituted phthalonitriles, this precipitation was less efficient. Moreover, the isolated intermediates like **10** slowly darkened in storage (accelerated in light), and in fact convert readily to phthalocyanine even at moderate temperatures. Intermediate **10** could be used immediately in condensation with an aminoisoindoline, and under the standard protocol (methanol, reflux), the reaction proceeded smoothly but did not precipitate the product. Chromatographic separation was therefore needed, and AB dimer **12** was isolated in modest yield (∼20%) in addition to the ABA trimer product **13**. Reasoning that the nucleophilic methanol solvent was responsible for the observed scrambling, the reaction was performed in dichloromethane in the presence of molecular sieves to remove the methanol formed in the reaction. This simple switch improved the yield of AB dimer **12** to ∼60%, and the product separated easily from other reaction products by crystallization. An alternative procedure, designed to eliminate the formation of the ABA trimer product, also proved successful. Dimethoxyisoindolamine intermediate **10** could be smoothly converted to its N‐Boc derivative by treatment with Boc–anhydride in glycerol [28]. Condensation with aminoisoindoline **1a**, again using dry DCM and molecular sieves, produced the protected AB dimer **14** (∼60%) and no ABA trimer. Deprotection using NaO*t*Bu/THF/H_2_O [29] gave AB dimer **12**.

### 2.2 TBTAP Formation From AB Intermediates

Investigation of hybrid macrocycle formation from differentially substituted AB intermediates started with the tetramethyltetralin derivative **12**. ABBA TBTAP **17** was expected from this reaction, and the product has been previously isolated as the dominant product from direct reactions between aminoisoindoline **1a** and phthalonitrile **9** (alongside the ABBB TBTAP **18**) [17]. Treatment of AB dimer **12** with MgBr_2_ in refluxing xylene produced a deep green reaction solution from which a single macrocyclic product was isolated alongside the ABA trimer side product (20%). Remarkably, however, the TBTAP was characterized as the ABBB (3:1) hybrid, produced exclusively in 45% yield (Scheme 6). This outcome was mirrored in reactions of the other AB intermediates **15** and **16**, where once again the 3:1 ABBB TBTAP hybrids were the only isolated macrocyclic products. These observations clearly indicate that the proposed AB + AB condensation pathway (Scheme 2) is not operating as a route to ABBA TBTAPs, and they must be formed by another pathway. Dominance of the ABBA (2:2) hybrid in reactions between aminoisoindoline **1a** and phthalonitrile **9** was verified again. Its origin is not obvious, but one possible pathway involves condensation between the growing aminoisoindoline–phthalonitrile oligomer and another molecule of aminoisoindoline. At the AB stage (either in the reaction or from pre‐isolated AB—see later), this results in the isolated ABA trimer side product. Condensation with **1a** at the ABB stage can result in ABBA intermediate that was thought to originate from AB+AB, and it remains the most likely precursor to the observed 2:2 hybrids. The alternative pathways that dominate in the absence of significant concentrations of **1a** lead to open ABBB via ABB, by reaction with either phthalonitrile or any oligomeric intermediate (shown for the AB intermediate **12**; natural bond orbital analysis indicates that these two sites and the amine bearing carbon have essentially equivalent partial positive charge and hence electrophilicity, see Supporting Information) and then macrocyclization to give ABBB TBTAPs like **18**. Indeed, the potential competition of these pathways was supported by observation of peaks corresponding to ABB and ABBA intermediates in MALDI‐MS analyses of reaction aliquots of reactions performed at lower temperatures. It is clear, therefore, that a complex balance of competing pathways is operating, but it remains remarkable that such a high level of control is possible in the two processes.

**SCHEME 6:**
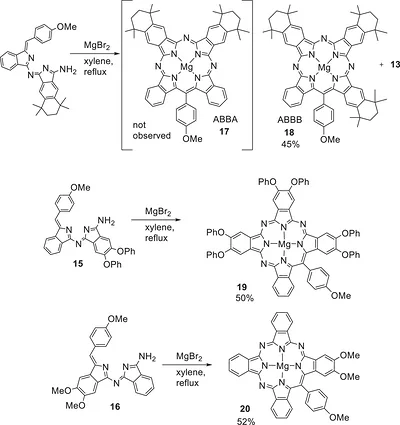
Macrocycle formation from preformed AB dimer intermediates unexpectedly yields 3:1 ABBB TBTAPs as the only hybrid product.

### 2.3 ABA Trimers (Tribenzodiazatripyrrins)

ABA trimers like **8** and **13** were regularly observed as significant side products in the synthesis of AB intermediates and in their subsequent reactions (Scheme 7, top). They are intriguing chromophores in their own right, and their synthesis and spectroscopic properties were separately evaluated in a short study. The ABA trimers could be isolated from high‐temperature syntheses using AB intermediates like **7**. However, the mechanism presented in Scheme 7 suggested that a targeted synthesis employing intermediates like **7**/**22** + additional aminoisoindoline would be efficient, and this proved to be the case. Reactions using 2 equivalents of aminoisoindoline and one equivalent of activated phthalonitrile (like **6**) were similarly efficient and follow essentially the same path. Scheme 8 summarizes the routes, and also shows additional derivatives **24** and **25**, the latter bearing a π‐extended naphthyl fragment in the central “B” unit. ABA trimers in this short series show broad, relatively featureless electronic absorption spectra that tail to ∼650 nm, therefore covering a significant proportion of the visible range. Scheme 8 shows representative examples alongside spectra for typical AB dimers that also show broad absorption profiles. Absorption spectra are relatively insensitive to variation of substituents, including the introduction of the naphthalene in **25**. Neither series shows appreciable emission. Crystals suitable for x‐ray diffraction were obtained for selected examples, and the crystal structure for **25** is also shown in Scheme 8. The observed stereochemistry presents a helical conjugated skeleton, as observed for the symmetrical AA dimers previously reported [30, 31]. At the molecular level, the helical arrangement allows a near‐perfect slipped stacked arrangement of the terminal aryl fragments. The crystal itself is made up of extended stacks of molecules of identical helicity, with neighbouring columns comprised of molecules with the opposite stereochemistry. The stereochemistry and helicity are clearly maintained in solution, evidenced by ^1^H NMR spectroscopy that clearly shows peak shifts consistent with overlapping terminal aromatic groups. For example, signals for the terminal methoxyphenyl fragment of AB dimer **7a** shift upfield by 0.4 and 0.6 ppm in the corresponding trimer **8b** as a result of this overlap (Scheme 8).

**SCHEME 7:**
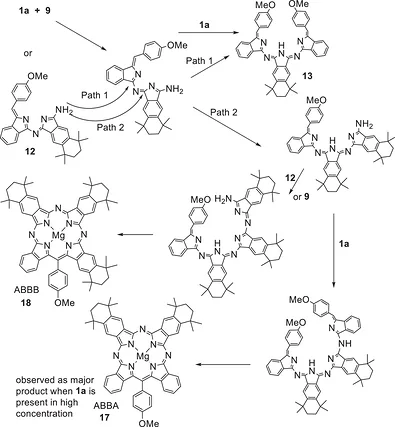
Plausible mechanistic pathways that lead to exclusive ABBB TBTAP (from AB dimer intermediates like **12**) and dominance of ABBA TBTAP in the aminoisoindoline + phthalonitrile reaction.

**SCHEME 8:**
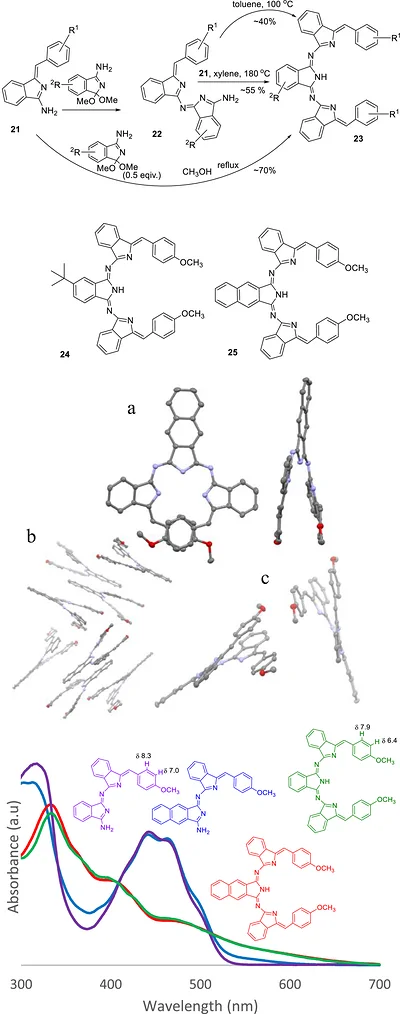
Synthetic routes to ABA trimers, crystal structure of trimer **25** (a. top and side view of single molecule; b. crystal packing in columns; c. pair of molecules in neighbouring columns with opposite helicity), and absorption spectra (in dichloromethane) of AB dimers and ABA trimers plus key NMR chemical shift changes as a result of helical architecture being maintained in solution.

## 3 Conclusion

Condensation between aminoisoindolines and activated phthalonitrile derivatives smoothly yields intermediates that are the expected precursors to “ABBA” tetrabenzotriazaporphyrin (TBTAP) hybrids. Remarkably, when these intermediates are subjected to macrocyclization conditions, the derivative isolated is exclusively the ABBB (3:1) phthalocyanine hybrid, which can only result from selective scrambling. The outcome is surprising, and the synthesis protocol in fact offers general improvements because yields are good, and competing phthalocyanine formation is minimized. The major side‐product in the reactions is the novel ABA trimeric systems (tripyrrin analogues), and their optimized synthesis is separately developed. The ABA trimers present broad absorption spectra up to ∼ 650 nm, and the conjugated core extends from one terminal aryl unit to the second in a helix, allowing near‐perfect overlap of the aromatic termini.

## Conflicts of Interest

The authors declare no conflicts of interest.

## Supporting information




**Supporting File**: chem71357‐sup‐0001‐SuppMat.pdf
The authors have cited additional references within the Supporting Information [32, 33, 34, 35, 36]. Crystal structures have been deposited at the joint CCDC/FIZ Karlsruhe deposition service with the following reference numbers: **8a** (2514571), **25** (2521412).

## Data Availability

The data that support the findings of this study are available from the corresponding author upon reasonable request.

## References

[1] K. M. Kadish , K. M. Smith , and R. Guilard , ed., The Porphyrin Handbook, Vol. 1–20 (Academic Press, 2000).

[2] K. M. Kadish , K. M. Smith , and R. Guilard , ed., Handbook of Porphyrin Science: With Applications to Chemistry, Physics, Materials Science, Engineering, Biology and Medicine, Vol. 1–45 (World Scientific, 2010).

[3] A. Treibs and F.‐H. Kreuzer , “Difluorboryl‐Komplexe Von Di‐Und Tripyrrylmethenen,” Justus Liebigs Annalen der Chemie 718 (1968): 208. 10.1002/jlac.19687180118 5704489

[4] A. Loudet and K. Burgess , “BODIPY Dyes and Their Derivatives: Syntheses and Spectroscopic Properties,” Chemical Reviews 107 (2007): 4891–4932, 10.1021/cr078381n. 17924696

[5] P. Kaur and K. Singh , “Recent Advances in the application of BODIPY in Bioimaging and Chemosensing,” Journal of Materials Chemistry C 7 (2019): 11361–11405, 10.1039/C9TC03719E.

[6] I. S. Yadav and R. Misra , “Design, Synthesis and Functionalization of BODIPY Dyes: Applications in Dye‐Sensitized Solar Cells (DSSCs) and Photodynamic Therapy (PDT),” Journal of Materials Chemistry C 11 (2023): 8688–8723, 10.1039/D3TC00171G.

[7] A. N. Cammidge , I. Chambrier , M. J. Cook , and L. Sosa‐Vargas , “Synthesis and Properties of the Hybrid Phthalocyanine‐Tetrabenzoporphyrin Macrocycles” in Handbook of Porphyrin Science, Vol. 16 ed. K. M. Kadish , K. M. Smith , and R. Guilard , (World Scientific Publishing, 2012), 331–404.

[8] M. Kaur , A. Janaagal , N. Balsukuri , and I. Gupta , “Evolution of Aza‐BODIPY Dyes‐A Hot Topic,” Coordination Chemistry Reviews 498 (2024): 215428, 10.1016/j.ccr.2023.215428.

[9] A. N. Cammidge , M. J. Cook , D. L. Hughes , F. Nekelson , and M. Rahman , “A Remarkable Side‐Product From the Synthesis of an Octaalkylphthalocyanine: Formation of a Tetrabenzotriazaporphyrin,” Chemical Communications (2005): 930, 10.1039/b414820g. 15700085

[10] A. N. Cammidge , I. Chambrier , M. J. Cook , D. L. Hughes , M. Rahman , and L. Sosa‐Vargas , “Phthalocyanine Analogues: Unexpectedly Facile Access to Non‐Peripherally Substituted Octaalkyl Tetrabenzotriazaporphyrins, Tetrabenzodiazaporphyrins, Tetrabenzomonoazaporphyrins and Tetrabenzoporphyrins,” Chemistry—A European Journal 17 (2011): 3136–3146, 10.1002/chem.201002176. 21322067

[11] J. Mack , L. Sosa‐Vargas , S. J. Coles , et al., “Synthesis, Characterization, MCD Spectroscopy, and TD‐DFT Calculations of Copper‐Metalated Nonperipherally Substituted Octaoctyl Derivatives of Tetrabenzotriazaporphyrin, cis—and trans ‐Tetrabenzodiazaporphyrin, Tetrabenzomonoazaporphyrin, and Tetrabenzoporphyrin,” Inorganic Chemistry 51 (2012): 12820–12833, 10.1021/ic301712h. 23140389

[12] C. E. Dent , “1. Preparation of Phthalocyanine‐Like Pigments Related to the Porphyrins,” Journal of the Chemical Society (Resumed) (1938): 1–6, 10.1039/jr9380000001.

[13] P. A. Barrett , R. P. Linstead , G. A. P. Tuey , and J. M. Robertson , “371. Phthalocyanines and Related Compounds. Part XV. Tetrabenztriazaporphin: Its Preparation From Phthalonitrile and a Proof of Its Structure. With a Note on a Preliminary X‐Ray Investigation,” Journal of the Chemical Society (Resumed) (1939): 1809, 10.1039/jr9390001809.

[14] A. Díaz‐Moscoso , G. J. Tizzard , S. J. Coles , and A. N. Cammidge , “Synthesis of Meso‐Substituted Tetrabenzotriazaporphyrins: Easy Access to Hybrid Macrocycles,” Angewandte Chemie International Edition 52 (2013): 10784–10787, 10.1002/anie.201306151. 24038943

[15] N. Alharbi , A. Díaz‐Moscoso , G. J. Tizzard , S. J. Coles , M. J. Cook , and A. N. Cammidge , “Improved Syntheses of Meso‐Aryl Tetrabenzotriazaporphyrins (TBTAPs),” Tetrahedron 70 (2014): 7370–7379, 10.1016/j.tet.2014.06.086.

[16] S. Remiro‐Buenamañana , A. Díaz‐Moscoso , D. L. Hughes , et al., “Synthesis of Meso‐Substituted Subphthalocyanine–Subporphyrin Hybrids: Boron Subtribenzodiazaporphyrins,” Angewandte Chemie International Edition 54 (2015): 7510–7514, 10.1002/anie.201502662. PMC4643193 25981391

[17] F. Alkorbi , A. Díaz‐Moscoso , J. Gretton , et al., “Complementary Syntheses Giving Access to a Full Suite of Differentially Substituted Phthalocyanine‐Porphyrin Hybrids,” Angewandte Chemie International Edition 60 (2021): 7632–7636, 10.1002/anie.202016596. PMC8048519 33428323

[18] V. N. Nemykin , S. V. Dudkin , F. Dumoulin , C. Hirel , A. Gul Gurek , and V. Ahsen , “Synthetic Approaches to Asymmetric Phthalocyanines and Their Analogues,” ARKIVOC (2014): 142–204, 10.3998/ark.5550190.p008.412.

[19] S. W. Oliver and T. D. Smith , “Oligomeric Cyclization of Dinitriles in the Synthesis of Phthalocyanines and Related Compounds: The Role of the Alkoxide Anion,” Journal of the Chemical Society, Perkin Transactions 2 (1987): 1579, 10.1039/p29870001579.

[20] K. J. M. Nolan , M. Hu , and C. C. Leznoff , “"Adjacent" Substituted Phthalocyanines,” Synlett (1997): 593–594, 10.1055/s-1997-3226.

[21] T. Fukuda and N. Kobayashi , “Efficient Synthesis of a Donor‐Acceptor Phthalocyanine Having Adjacently‐Fused Pyrazine Rings,” Chemistry Letters (2002): 866–867, 10.1246/cl.2002.866.

[22] Y. Kikukawa , T. Fukuda , A. Fuyuhiro , N. Ishikawa , and N. Kobayshi , “Facile One‐Pot Preparation of Thermally and Photochemically Convertible Soluble Precursors of Copper Phthalocyanine and Naphthalocyanine,” Chemical Communications 47 (2011): 8518, 10.1039/c1cc12970h. 21725534

[23] T. Fukuda , Y. Kikukawa , R. Tsuruya , N. Ishikawa , and N. Kobayshi , “Effect of Chain Length on Thermal Conversion of Alkoxy‐Substituted Copper Phthalocyanine Precursors,” Inorganic Chemistry 50 (2011): 11832–11837, 10.1021/ic202054t. 22032334

[24] F. Baumann , B. Bienert , G. Rosch , H. Vollmann , and W. Wolf , “Isoindolenine als Zwischenprodukte der Phthalocyanin‐Synthese,” Angewandte Chemie 68 (1956): 133–150, 10.1002/ange.19560680402.

[25] I. Chambrier and M. J. Cook , “Reaction of Phthalonitrile with Alkoxide Ions,” Journal of Chemical Research (1990): 322.

[26] E. N. Carrión , A. T. Tang , and S. M. Gorun , “Synthesis and X‐Ray Structure of a Fluorinated 1, 1‐dialkoxy‐3‐iminoisoindoline Acetal, an Elusive Phthalocyanine Precursor,” Tetrahedron 74 (2018): 3697–3700.

[27] M. A. Tyutina , T. V. Kudayarova , and E. A. Danilova , “Synthesis of АВВВ‐Type Macroheterocyclic Compounds Containing a 3‐alkyl‐1,3,4‐thidiazoline Fragment,” Russian Journal of General Chemistry 87 (2017): 1737–1741, 10.1134/S1070363217080175.

[28] A. P. Ingale , V. K. More , U. S. Gangarde , and S. V. Shinde , “Chemoselective N‐tert ‐butyloxycarbonylation of Amines in Glycerol,” New Journal of Chemistry 42 (2017): 10142–10147, 10.1039/C8NJ01585F.

[29] N. J. Tom , W. M. Simon , H. N. Frost , and M. Ewing , “Deprotection of a Primary Boc Group Under Basic Conditions,” Tetrahedron Letters 45 (2004): 905–906, 10.1016/j.tetlet.2003.11.108.

[30] B. N. Alanazi , A. O. Alsahli , S. Remiro‐Buenamañana , et al., “A Family of Symmetrical and Unsymmetrical Aza‐Dipyrromethenes and Aza‐BODIPYs With Through‐Bond and Face‐To‐Face π‐Interaction Between Termini,” Chemistry—A European Journal (2025): e202501862, 10.1002/chem.202501862. PMC12415328 40632928

[31] A. Díaz‐Moscoso , E. Emond , D. L. Hughes , G. J. Tizzard , S. J. Coles , and A. N. Cammidge , “Synthesis of a Class of Core‐Modified Aza‐BODIPY Derivatives,” Journal of Organic Chemistry 79 (2014): 8932–8936, 10.1021/jo501863t. 25180453

[32] S. A. Mikhalenko , L. I. Solov'eva , and E. A. Luk'yanets , “Symmetrical, Sterically Hindered Phthalocyanines,” Journal of the Russian Chemical Society 58 (1988): 2618.

[33] T. Schareina , A. Zapf , W. M¨agerlein , N. M¨uller , and M. Beller , “A State‐of‐the‐Art Cyanation of Aryl Bromides: A Novel and Versatile Copper Catalyst System Inspired by Nature,” Chemistry—A European Journal 13 (2007): 6249–6254, 10.1002/chem.200700079. 17480048

[34] I. Rose , C. G. Bezzu , M. Carta , et al., “Polymer Ultrapermeability From the Inefficient Packing of 2D Chains,” Nature Materials 16 (2017): 932–937, 10.1038/nmat4939. 28759030

[35] M. Hellal and G. D. Cuny , “Microwave Assisted Copper‐Free Sonogashira Coupling/5‐exo‐dig Cycloisomerization Domino Reaction: Access to 3‐(phenylmethylene)isoindolin‐1‐Ones and Related Heterocycles,” Tetrahedron Letters 52 (2011): 5508–5511, 10.1016/j.tetlet.2011.08.070.

[36] A. I. Koptyaev , N. E. Galanin , and G. P. Shaposhnikov , “Synthesis and Association in Dichloromethane Solution of Low‐Symmetry Phenoxy(Fluoro)‐Substituted Nickel Phthalocyaninates,” Russian Journal of General Chemistry 86 (2016): 854–858, 10.1134/S1070363216040162.

