# Supplementary material for: Stepwise Synthesis of Tetrabenzotriazaporphyrins (TBTAPs) and Their Open 2‐ and 3‐Ring Fragments
Source: Chemistry. 2026 Jul 6;32(38):e71357. doi: 10.1002/chem.71357 (PMC13660487; doi:10.1002/chem.71357)
Supplement: Supplementary file 1 [file CHEM-32-e71357-s001.pdf]

Stepwise Synthesis of Tetrabenzotriazaporphyrins (TBTAPs) and Their Open 2- and 3-Ring Fragments

Jacob Gretton, Ala Alturk, Sonia Remiro-Buenamañana, Alejandro Díaz-Moscoso[†], Amirah Aldawsari, Maha S. Albayaa, Isabelle Chambrier, David L. Hughes, Simon J. Coles, Graham J. Tizzard and Andrew N. Cammidge*

Dr J. Gretton, Dr. Ala Alturk, Dr S. Remiro-Buenamañana, Dr A. Díaz-Moscoso, A. Aldawsari, M. S. Albayaa, Dr I. Chambrier, Dr D. L. Hughes, Prof. A. N. Cammidge, School of Chemistry, Pharmacy and Pharmacology University of East Anglia Norwich Research Park, Norwich E-mail: a.cammidge@uea.ac.uk

Prof. S. J. Coles, Dr G. J. Tizzard, UK National Crystallography Service, School of Chemistry, University of Southampton, Southampton, SO17 1BJ (UK)

[†]Current address: Instituto de Investigaciones Químicas (IIQ-CICIC), CSIC-US; Av. Americo Vespucio, 49; 41092, Seville, Spain

Supporting Information

Contents

General Methods **2**

Phthalonitriles and derivatives **3**

Aminoisoindolines **10**

AB Dimers **12**

ABA Trimers (tribenzodiazatripyrrins) **25**

MgTBTAPs from AB dimers **35**

Benzo substituted MgTBTAPs from AB dimers **37**

Pathway analysis for reactions (scrambling) of AB dimers **42**

X-ray crystal structures **43**

General Methods

Reagents and solvents were obtained from commercial sources and used without further purification unless otherwise stated. Phthalonitrile was recrystallised from hot xylene. THF was freshly distilled from sodium and benzophenone. Ether refers to diethyl ether. Petroleum ether (PE) refers to 40-60°C petroleum ether. Reactions and distillation were carried out under an inert atmosphere (argon or nitrogen gas), in most air-sensitive reactions argon was preferred. Brine is a saturated aqueous solution of sodium chloride. Organic layers were dried using anhydrous magnesium sulphate. Evaporating of solvent was performed using a Buchi rotary evaporator at reduced pressure. ^{1}H NMR spectra were recorded either at 400 MHz on Bruker Ultrashield Plus™ 400 spectrometer or 500 MHz on a Bruker Ascend™ 500 spectrometer in 5 mm diameter tubes. Signals are quoted in ppm as δ downfield from tetramethylsilane (δ=0.00) and coupling constants J given in Hertz. ^{13}C spectra were recorded at 100.6 MHz or 125.7 MHz on the same spectrometers. NMR spectra were performed in solution using deuterated chloroform, methanol, dichloromethane, dimethyl sulfoxide or tetrahydrofuran at room temperature unless otherwise stated. Ultraviolet-Visible absorption spectra were recorded on Hitachi U-3310 Spectrophotometer in solvent as stated. MALDI-TOF mass spectra were carried out using a Shimadzu Biotech Axima instrument. Characterization by mass spectrometry was achieved by comparison of isotopic distribution to theory. IR spectra were recorded using a Perkin-Elmer Spectrum BX FT-IR spectrometer. Thin layer chromatography (TLC) was performed using aluminium sheets coated with Alugram® Sil G/UV254 (Macherey-Nagel), and the compounds were visualised under short-wavelength UV-light at 245 nm or 366 nm. Column chromatography was carried out using silica gel 60Å mesh 70 – 230 (63 – 200 µm) under gravity or moderate pressure at ambient temperature. Solvent ratios are given as v:v. Melting points were taken on a Reichart Thermovar microscope with a thermopar based temperature control. Reactions using microwave irradiation were carried out in Biotage Initiator+ Microwave system.

Phthalonitriles and derivatives

1-Imino-3,3-dimethoxyisoindoline (6a)[11]

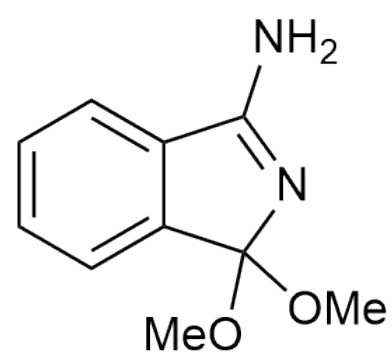

Following a modification of the reported procedure,[11b] sodium methoxide (2.02 g, 0.037 mol) was fully dissolved in methanol (8 ml) at room temperature. Phthalonitrile (1 g, 0.0078 mol) was added to the solution and the mixture was stirred for 2 h at room temperature. The resulting near-colourless solid was collected by vacuum filtration, washed with a minimum volume of ice-cold water and dried in air to yield **6a** (0.57 g, 38 %) as a pale green solid.

^{1}H NMR (500 MHz, $CDCl_3$) δ 7.54 (dt, J = 7.3, 1.1 Hz, 1H), 7.50 – 7.35 (m, 3H), 5.95 (br s, 2H), 3.38 (s, 6H). **^{13}C NMR** (125.7 MHz, $CDCl_3$) δ 163.50, 138.51, 132.93, 130.69, 129.63, 122.69, 119.94, 116.09, 51.31. **MP:** Slow decomp.

Phthalonitrile-ethane diol adduct (6b)

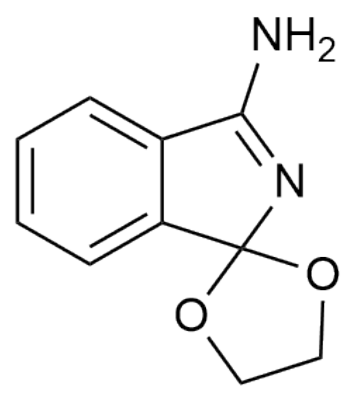

Sodium methoxide (2.02 g, 0.037 mol) was fully dissolved in ethane diol (8 ml) at room temperature. Phthalonitrile (1 g, 0.0078 mol) was added to the solution and the mixture was stirred overnight at room temperature and then left in a refrigerator at 5°C for 24h to complete the precipitation. The resulting near-colourless solid was collected by vacuum filtration, and recrystallised from ethyl acetate to yield **6b** (1.25 g, 83 %) as colourless crystals.

^{1}H NMR (500 MHz, Acetone-d_6) δ 7.66 – 7.61 (m, 1H), 7.49 – 7.42 (m, 3H), 6.55(br s, 2H), 4.23 – 4.14 (m, 4H). **^{13}C NMR** (125.7 MHz, DMSO-d_6) δ 162.56, 146.46, 134.75, 129.73, 129.50, 122.93, 121.93, 120.32, 64.48. **MP:** 148-149.5°C.

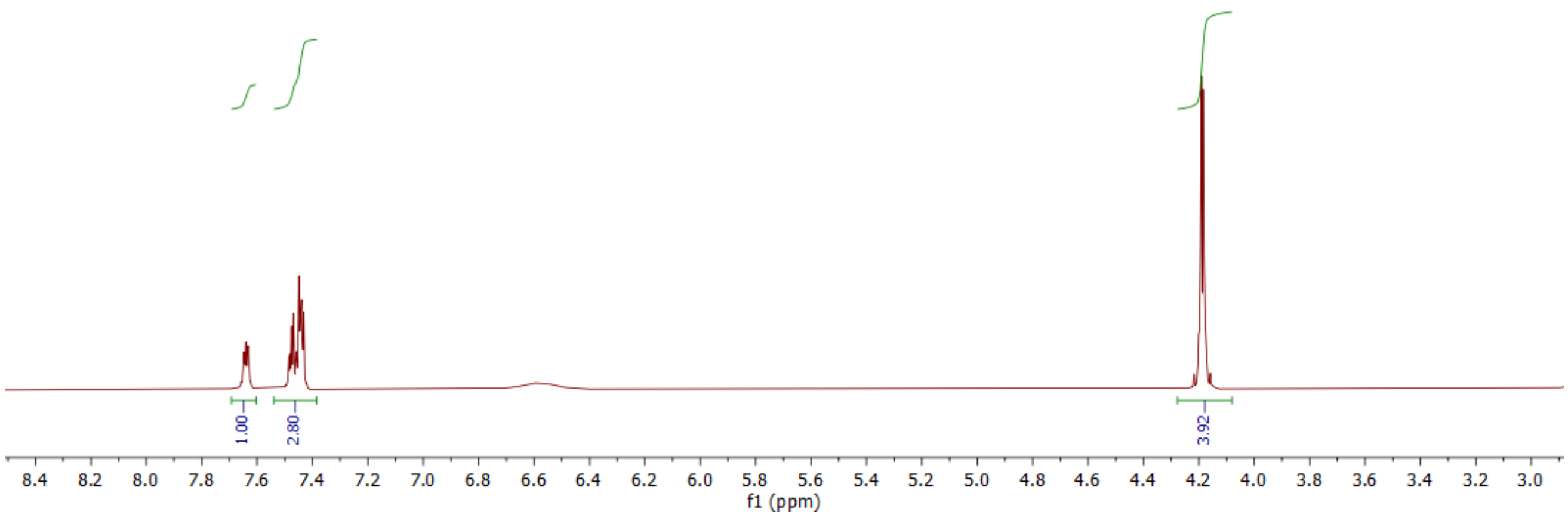

1.00
2.80
3.92
8.4 8.2 8.0 7.8 7.6 7.4 7.2 7.0 6.8 6.6 6.4 6.2 6.0 5.8 5.6 5.4 5.2 5.0 4.8 4.6 4.4 4.2 4.0 3.8 3.6 3.4 3.2 3.0
f1 (ppm)

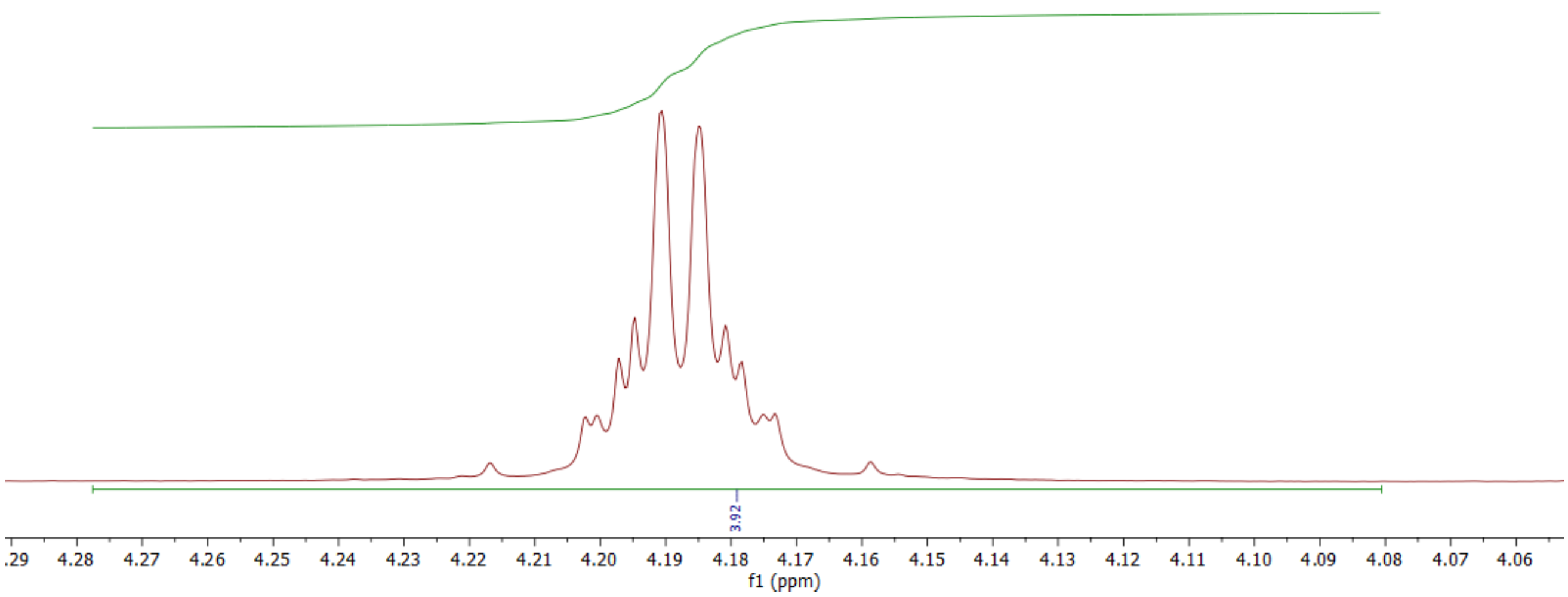

3.92
.29 4.28 4.27 4.26 4.25 4.24 4.23 4.22 4.21 4.20 4.19 4.18 4.17 4.16 4.15 4.14 4.13 4.12 4.11 4.10 4.09 4.08 4.07 4.06
f1 (ppm)

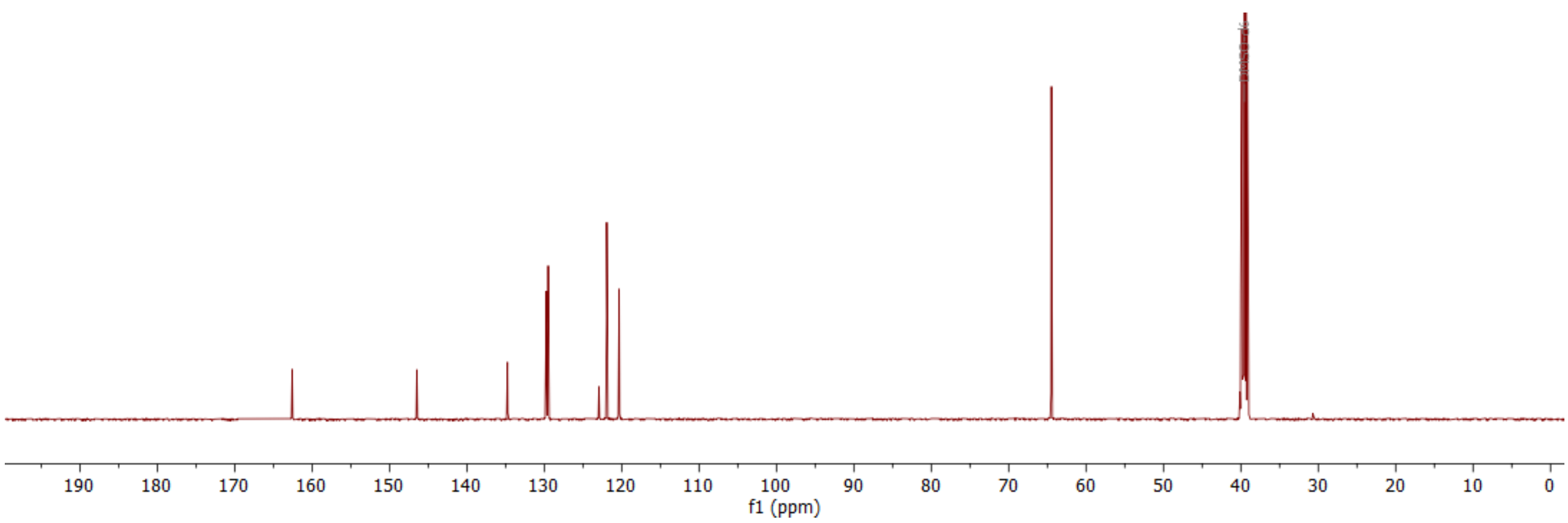

190 180 170 160 150 140 130 120 110 100 90 80 70 60 50 40 30 20 10 0
f1 (ppm)

Phthalonitrile-propane-1,3-diol adduct (6c)

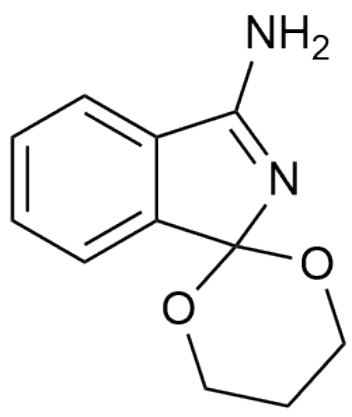

Sodium methoxide (2.02 g, 0.037 mol) was fully dissolved in 1,3-propane diol (8 ml) at room temperature. Phthalonitrile (1 g, 0.0078 mol) was added to the solution and the mixture was stirred overnight at room temperature and then left in a refrigerator at 5°C for 24h to complete the precipitation. The resulting near-colourless solid was collected by vacuum filtration and recrystallised from acetone to yield **6c** (1.20 g, 75 %) as colourless crystals.

^{1}H NMR (500 MHz, Acetone-d_6) δ 7.59 (ddd, J = 5.3, 3.1, 0.8 Hz, 1H), 7.48 (ddd, J = 5.2, 2.5, 0.7 Hz, 1H), 7.40 (dd, J = 5.5, 3.0 Hz, 2H), 6.34 (s, 2H), 4.55 (dddd, J = 12.7, 9.9, 2.8, 1.5 Hz, 2H), 3.85 – 3.79 (m, 2H), 2.28 – 2.19 (m, 1H), 1.51 (dtt, J = 13.2, 2.8, 1.4 Hz, 1H). **^{13}C NMR** (125.7 MHz, DMSO-d_6) δ 162.66, 148.78, 134.42, 130.01, 129.72, 122.05, 120.68, 115.63, 61.99, 25.82. **MP:** 183-186°C.

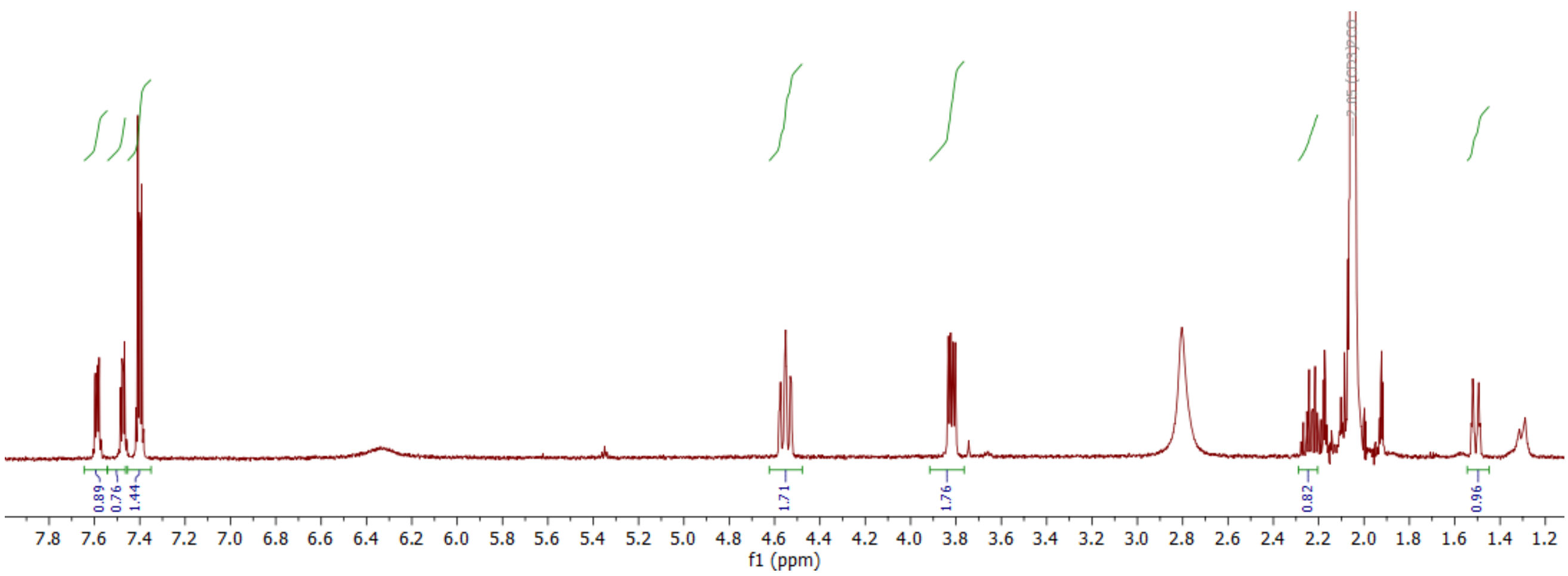

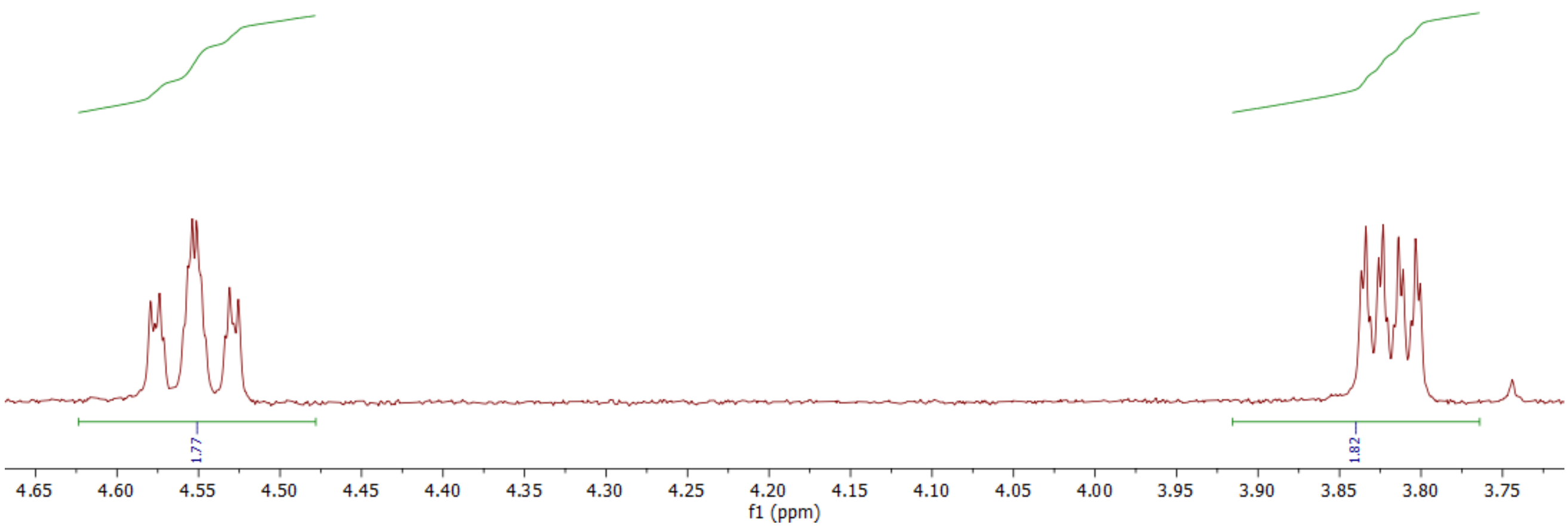
1.77
1.82
4.65 4.60 4.55 4.50 4.45 4.40 4.35 4.30 4.25 4.20 4.15 4.10 4.05 4.00 3.95 3.90 3.85 3.80 3.75
f1 (ppm)

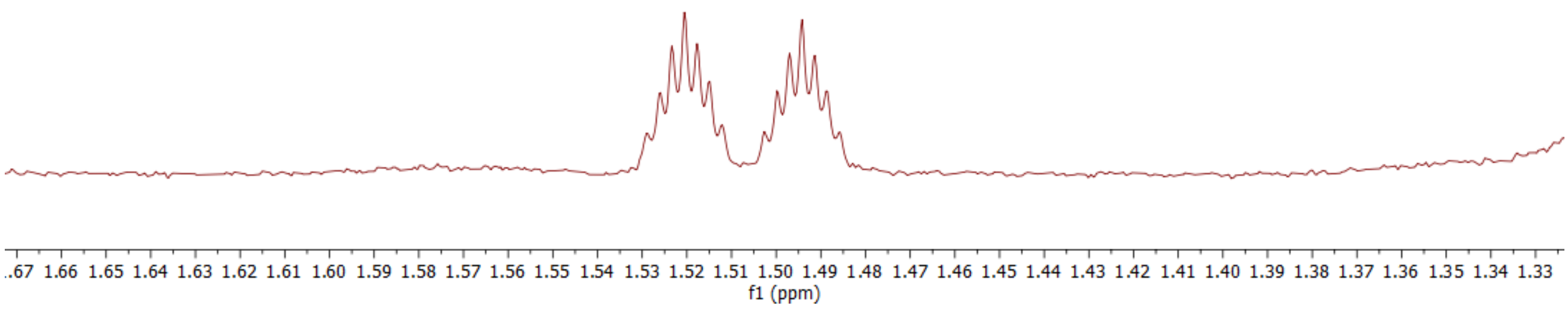
.67 1.66 1.65 1.64 1.63 1.62 1.61 1.60 1.59 1.58 1.57 1.56 1.55 1.54 1.53 1.52 1.51 1.50 1.49 1.48 1.47 1.46 1.45 1.44 1.43 1.42 1.41 1.40 1.39 1.38 1.37 1.36 1.35 1.34 1.33
f1 (ppm)

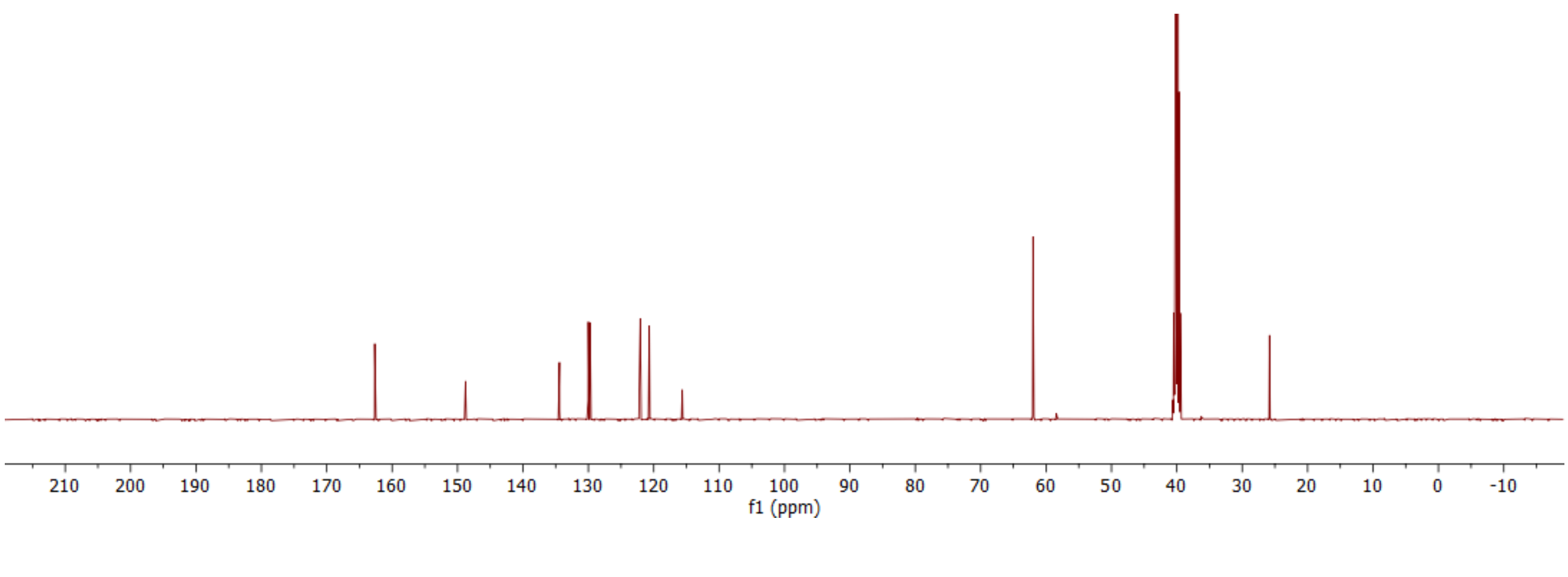
210 200 190 180 170 160 150 140 130 120 110 100 90 80 70 60 50 40 30 20 10 0 -10
f1 (ppm)

6,7-Dicyano-1,1,4,4-tetramethyl-1,2,3,4-tetrahydronaphthalene (9)[16]

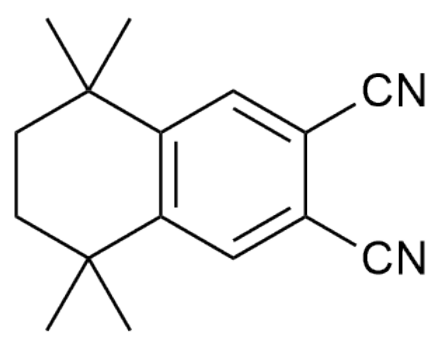

Using the Beller *et al.* cyanation procedure,[17] to a solution of 6,7-dibromo-1,1,4,4-tetramethyl-1,2,3,4-tetrahydronaphthalene[18] (5.5 g, 16 mmol) in anhydrous *p*-xylene (30 mL) was added finely ground and oven dried $K_3[Fe(CN)_6]$ (2.4 g, 7.3 mmol), CuI (304 mg, 1.6 mmol) and 1-butylimidazole (4.4 mL, 32 mmol). The suspension was heated at reflux for 72 hr then cooled to RT. Ether (100 mL) was added and the solution filtered. The solution was washed with water (3 x 50 mL) and brine (2 x 50 mL). The organic phase was collected and dried over $MgSO_4$, and the solvent was removed to yield an off-white solid. The crude product was subjected to silica gel chromatography eluting with PE/Et_2O, 7:1. Recrystallisation from MeOH yielded **9** as a colourless solid (2.9 g, 76 %).

^{1}H NMR (500 MHz, $CDCl_3$) δ 7.71 (s, 2H), 1.72 (s, 4H), 1.30 (s, 12H). **^{13}C NMR** (125.7 MHz, $CDCl_3$) δ 151.9, 132.7, 116.1, 112.6, 35.1, 34.0, 31.4. **MP:** 204-206 °C (lit. 206-208°C).[16]

Dimethoxyisoindoline (10)

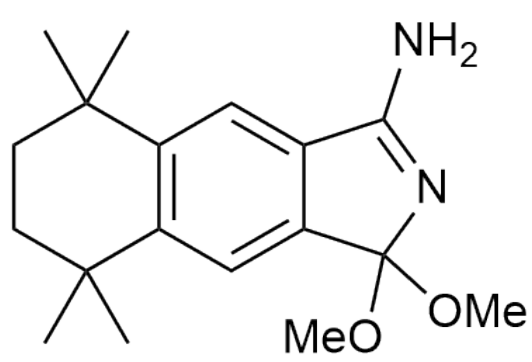

To a suspension of **9** (2.0 g, 8.4 mmol) in MeOH (10 mL) was added NaOMe (0.54 g, 10 mmol). The suspension was stirred at RT overnight and the precipitate filtered off and washed with water. The precipitate was dried under vacuum to yield **10** as an off-white solid (1.6 g, 63 %).

^{1}H NMR (500 MHz, $CDCl_3$) δ 7.49 (s, 1H), 7.32 (s, 1H), 5.61 (s, 2H), 3.40 (s, 6H), 1.73 (s, 4H), 1.33 (s, 12H). **^{13}C NMR** (125.7 MHz, $CDCl_3$) δ 169.06, 147.85, 146.78, 141.87, 131.08, 120.24, 119.05, 48.27, 34.57, 34.50, 34.47, 30.93, 30.87. **MP:** decomposes.

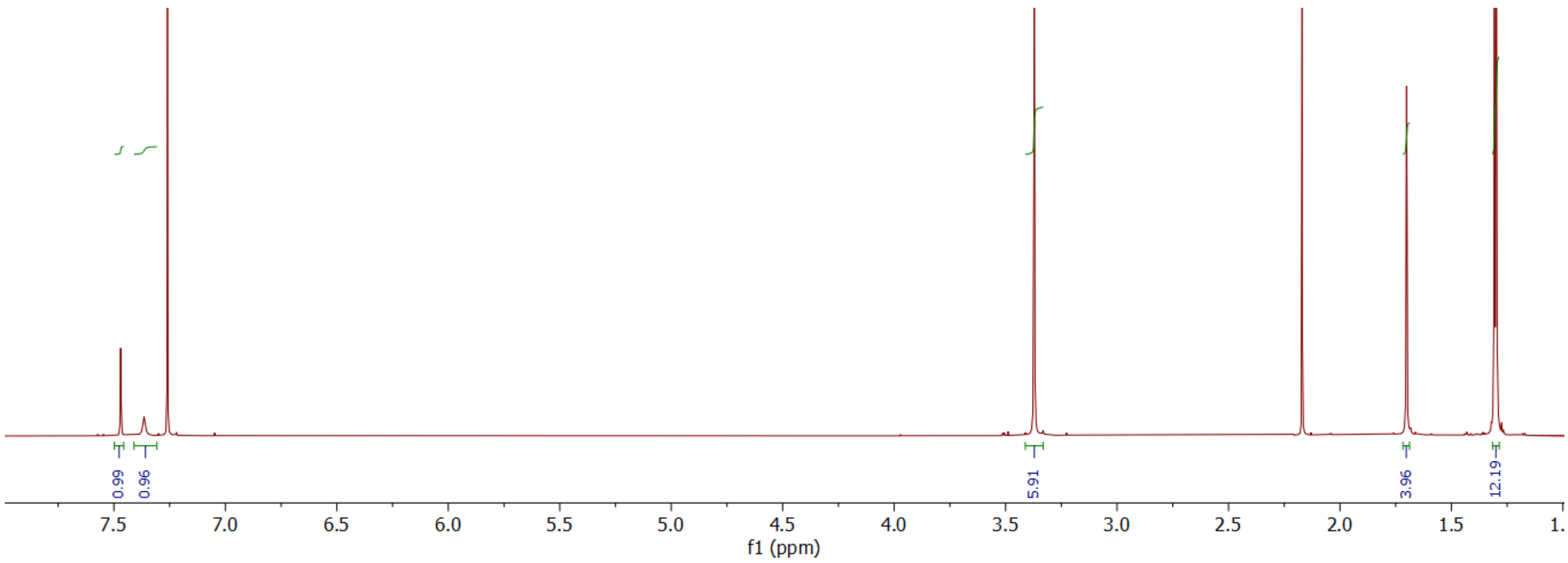
0.99
0.96
5.91
3.96
12.19
7.5 7.0 6.5 6.0 5.5 5.0 4.5 4.0 3.5 3.0 2.5 2.0 1.5 1.
f1 (ppm)

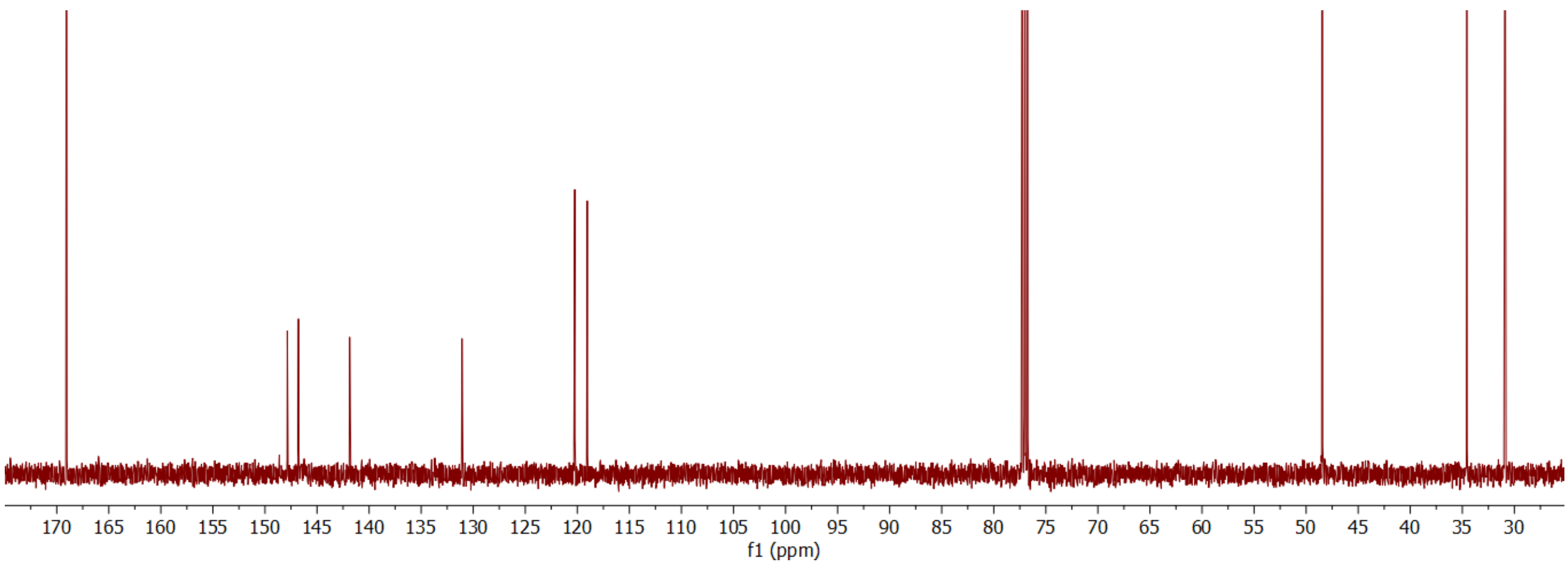
170 165 160 155 150 145 140 135 130 125 120 115 110 105 100 95 90 85 80 75 70 65 60 55 50 45 40 35 30
f1 (ppm)

NBoc dimethoxyisoindoline (11)

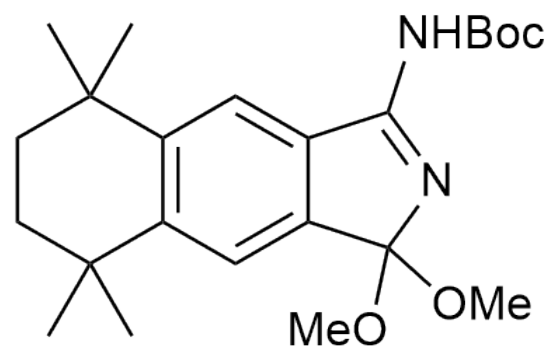

Using the reported procedure for Boc addition,[13] to a solution of **10** (1.5 g, 5 mmol) in glycerol (10 mL) was added di-tert-butyl-dicarbonate (1.2 g, 5.5 mmol). The solution was stirred at RT overnight, then extracted with hexane/EtOAc (9:1) (3 x 50 mL). The organics were collected and dried over $MgSO_4$ and the solvent removed to yield **11** as a colourless oil (1.8 g, 91 %).

^{1}H NMR (500 MHz, $CDCl_3$) δ 9.34 (s, 1H), 7.62 (s, 1H), 7.43 (s, 1H), 3.31 (s, 6H), 1.81 (s, 4H), 1.54 (s, 9H), 1.37 (s, 6H), 1.30 (s, 6H). **^{13}C NMR** (125.7 MHz, $CDCl_3$) δ 164.88, 164.11, 139.94, 134.04, 132.99, 130.73, 124.15, 122.47, 112.61, 80.53, 51.87, 35.27, 31.63, 31.58, 28.23. **MP:** < 25°C. **FT-IR: (ATR)**, ν (cm^{-1}): 1770 (C=O), 1085 (C-O, ether).

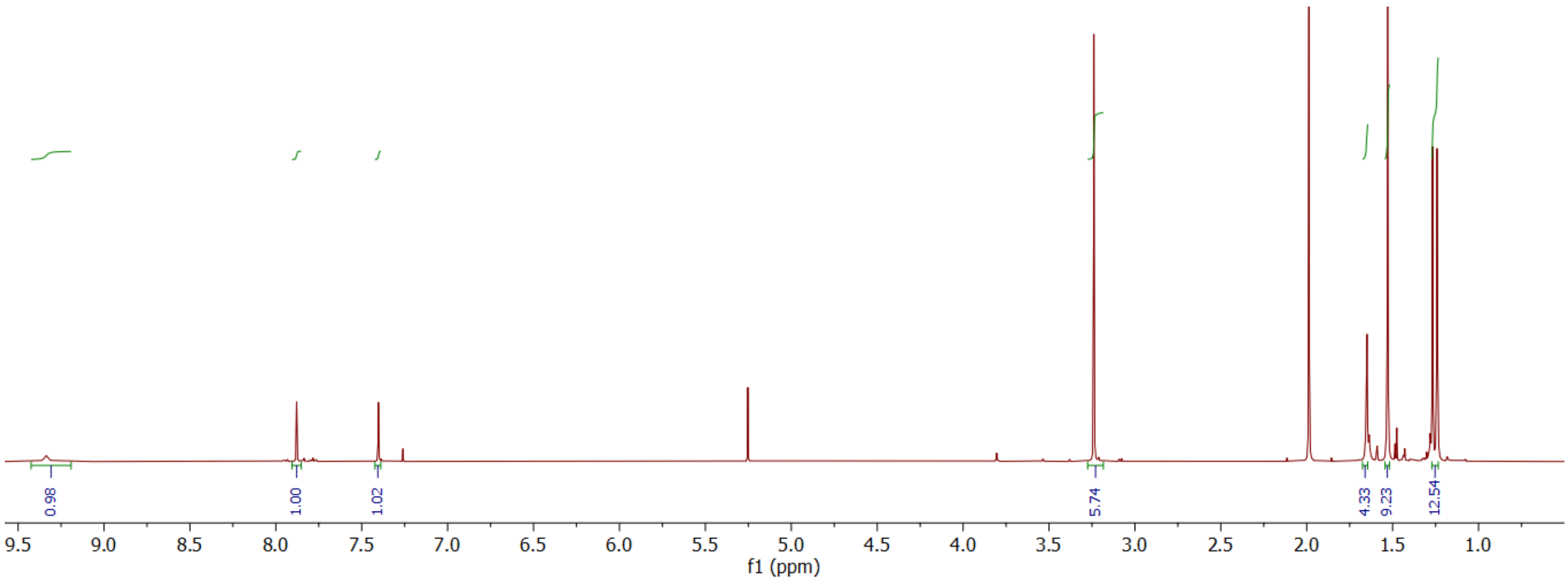

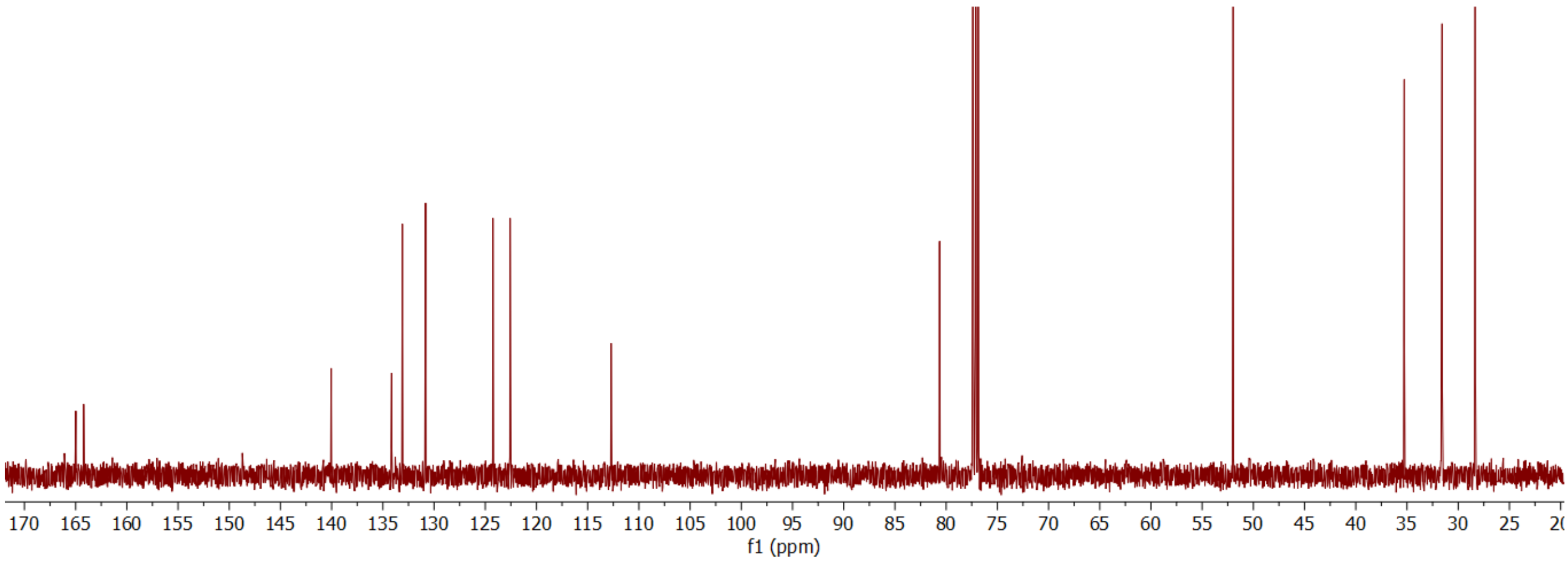

Aminoisoindolines

(Z)-1-[(4-*t*-Bu)benzylidene]-1*H*-isoindol-3-amine (1b)

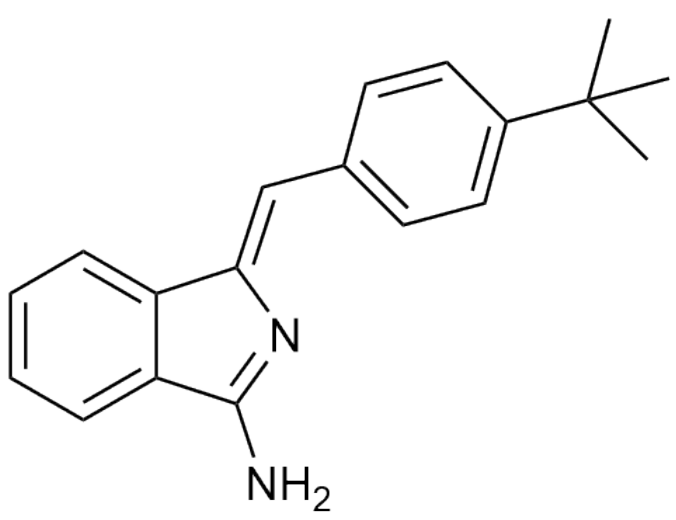

To a mixture of 2-bromobenzamidine (3.17 g, 13.5 mmol), BINAP (0.46 g, 0.74 mmol) and $PdCl_2(MeCN)_2$ (0.16 g, 0.67 mmol) in anhydrous DMF (50 mL) was added 1-(t-butyl)-4-ethynylbenzene (2.55 g, 16.2 mmol).[19] The solution was degassed with N_2 and DBU (5.13 g, 37.5 mmol) was added. The solution was heated to reflux for 6 hrs under N_2. Upon cooling EtOAc was added (250 mL) and the solution washed with sat $NaHCO_3$ (3 x 250 mL). The organics were collected, dried ($MgSO_4$), and the solvent removed *in vaccuo*. The dark brown residue was subjected to column chromatography (silica) eluting with DCM followed by EtOAc. The brown residue was recrystallised from DCM/hexane to yield **1b** as yellow needles (1.3 g, 34 %).

^{1}H NMR (400 MHz, $CDCl_3$) δ 7.97 (d, J = 8.8 Hz, 2H), 7.80 (d, J = 7.7 Hz, 1H), 7.59 (d, J = 7.5 Hz, 1H), 7.50 (td, J = 7.5, 1.0 Hz, 1H), 7.43 (d, J = 8.5 Hz, 2H), 7.39 (td, J = 7.4, 1.0 Hz, 1H), 6.78 (s, 1H), 5.67 (br s, 2H) 1.33 (s, 9H). **^{13}C NMR** (125.7 MHz, $CDCl_3$) δ 164.98, 150.43, 146.81, 143.00, 133.93, 131.03, 130.20, 129.04, 127.02, 125.42, 119.73, 118.82, 115.14, 34.68, 31.30. **MP:** 180°C. **MS (MALDI-TOF):** m/z = 276.2 $[M]^+$.

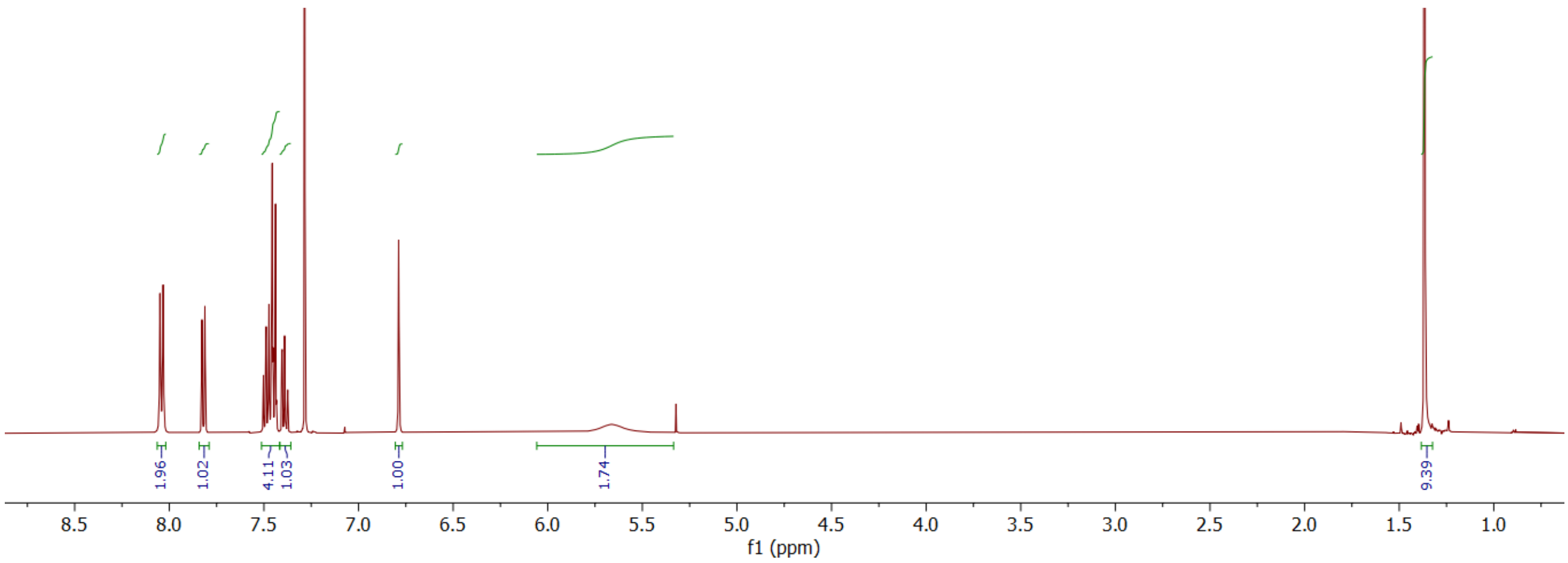
1.96
1.02
4.11
1.03
1.00
1.74
9.39
8.5
8.0
7.5
7.0
6.5
6.0
5.5
5.0
4.5
4.0
3.5
3.0
2.5
2.0
1.5
1.0
f1 (ppm)

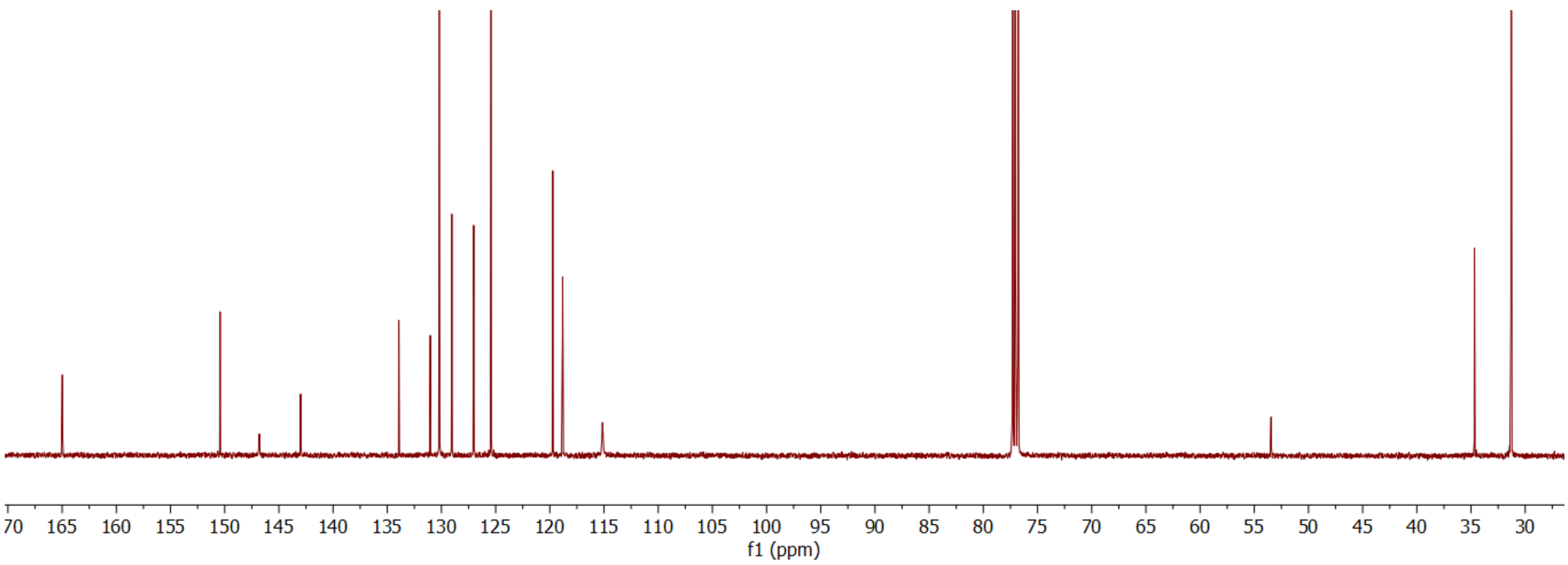
70
165
160
155
150
145
140
135
130
125
120
115
110
105
100
95
90
85
80
75
70
65
60
55
50
45
40
35
30
f1 (ppm)

AB Dimers

AB condensation product (7a)

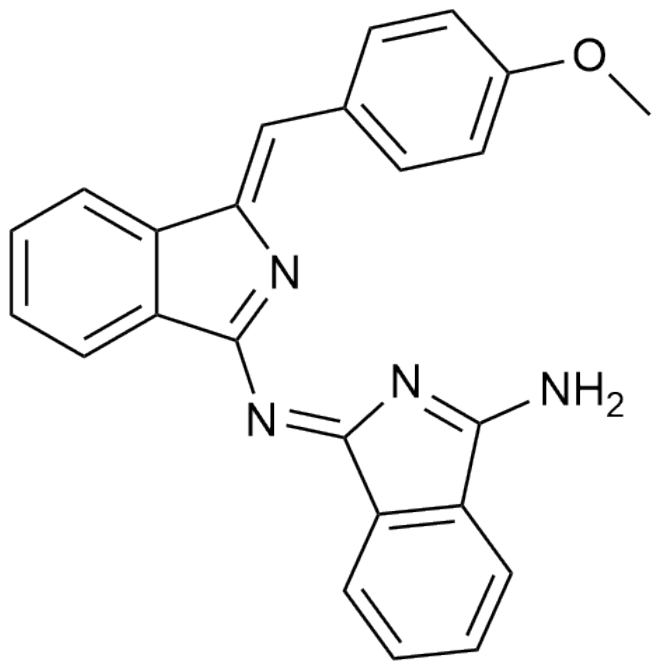

A solution of **1a**[7] (0.25 g, 1 mmol) and 1-imino-3,3-dimethoxyisoindoline **(6a)** (0.19 g, 1 mmol) in MeOH (5 mL) was stirred at RT overnight. Upon completion the precipitate was filtered and washed with cold methanol to yield **7a** as an orange solid (0.28 g, 74 %).

^{1}H NMR (500 MHz, DMSO-d_6) (peaks for major stereoisomer) δ 9.05 (br s, 2H), 8.26 (d, J = 8.9 Hz, 2H), 7.99 – 7.96 (m, 2H), 7.94 (s, 1H), 7.72 – 7.68 (m, 2H), 7.46 (t, J = 7.5 Hz, 1H), 7.40 – 7.31 (m, 2H), 7.27 (s, 1H), 7.01 (d, J = 8.7 Hz, 2H), 3.82 (s, 3H). **^{13}C NMR** (125.7 MHz, DMSO-d_6) δ 159.53, 154.14, 147.52, 146.36, 142.02, 134.27, 133.81, 132.97, 129.92, 129.67, 128.92, 128.25, 128.08, 126.99, 126.60, 126.59, 122.07, 121.47, 121.39, 119.31, 114.06, 55.20. **MP:** 168 - 172 °C. **MS (MALDI-TOF):** m/z = 378.94 $[M]^+$ **UV-Vis:** λ_{max} (nm) (ϵ ($dm^3.mol^{-1}.cm^{-1}$)) = 440 ($6.16x10^3$), 332 ($1.06x10^4$), 260 ($1.54x10^4$)

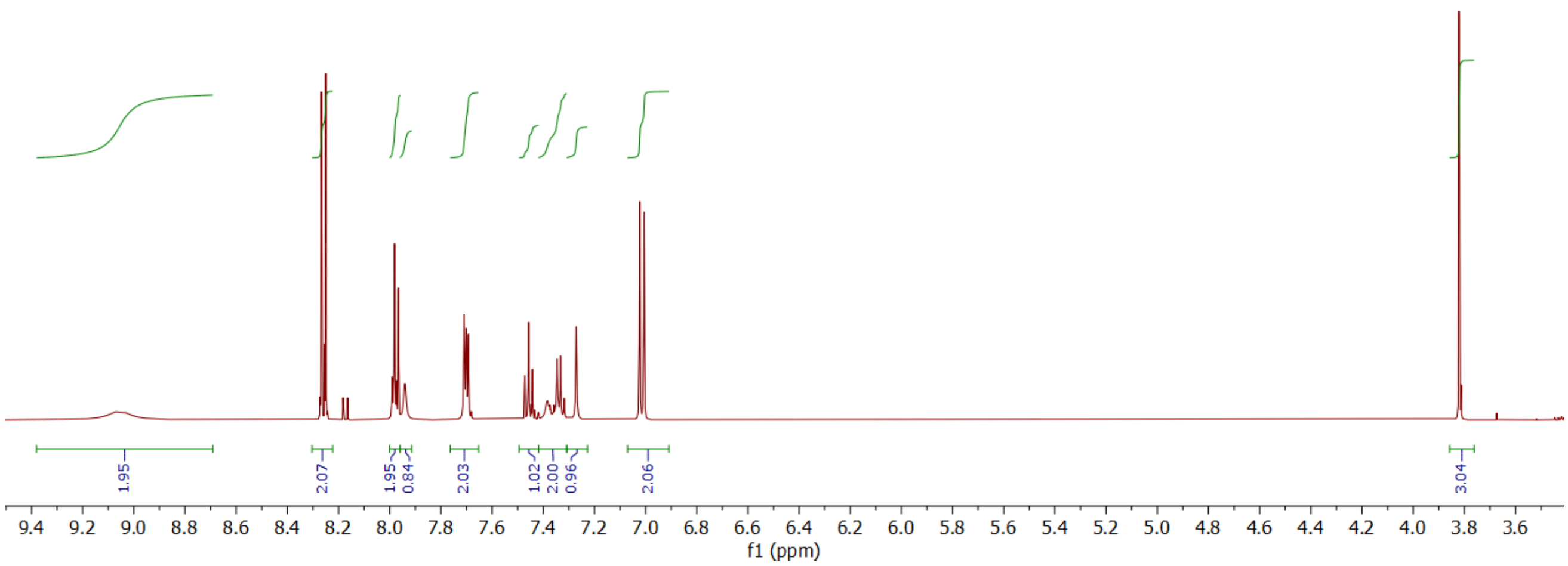

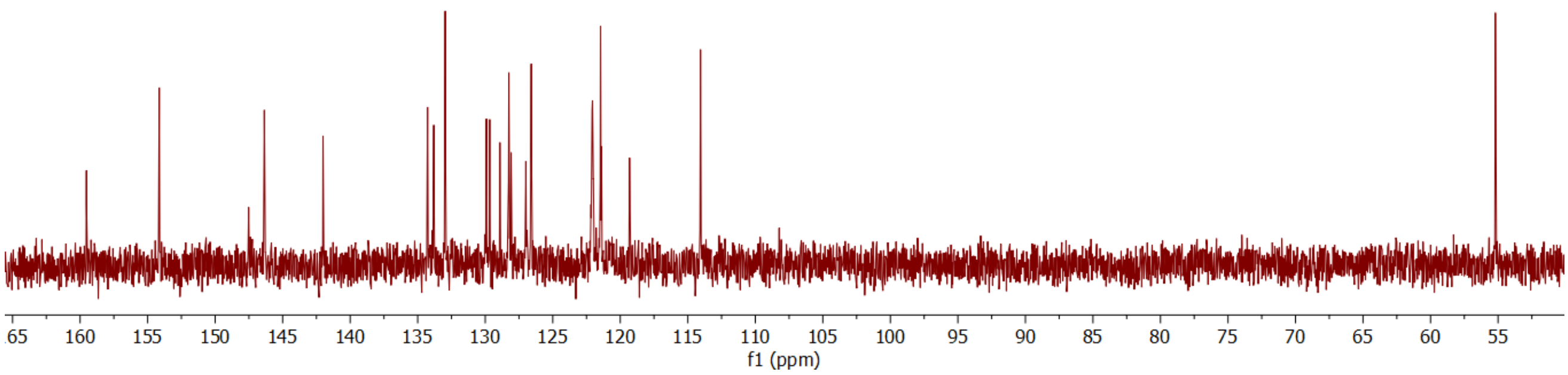

AB condensation product (7b)

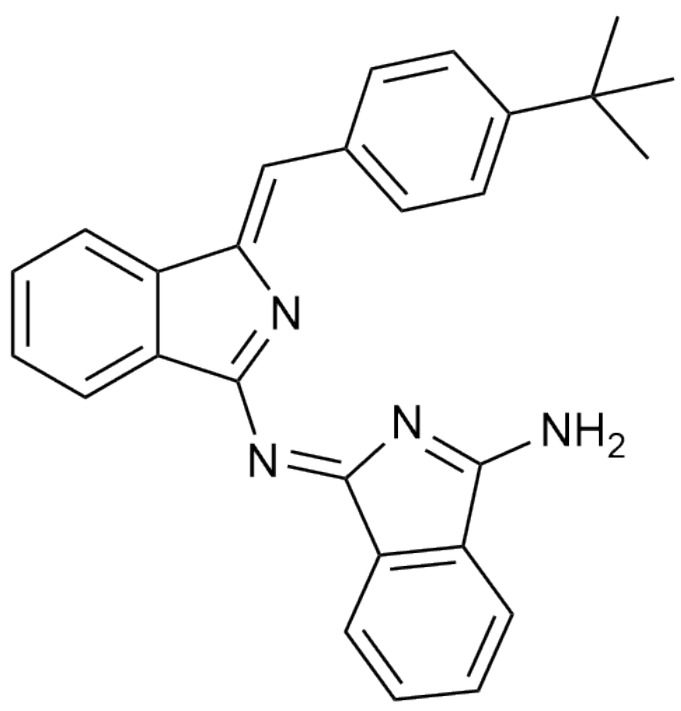

To a solution of **1b** (0.28 g, 1.05 mmol) and phthalonitrile, (0.13 g, 1.05 mmol) in MeOH (10 mL) was added NaOMe (65 mg, 1.2 mmol). The reaction was stirred at RT overnight to yield an orange precipitate. The precipitate was filtered to yield **7b** as an orange solid (0.16 g, 38 %).

^{1}H NMR (500 MHz, CD_2Cl_2) δ 8.15 – 8.13 (m, 1H), 8.05 (d, J = 8.3 Hz, 2H), 7.99 (d, J = 8.4 Hz, 1H), 7.91 (d, J = 2.7 Hz, 1H), 7.89 (d, J = 6.5 Hz, 1H), 7.75 – 7.69 (m, 2H), 7.55 (d, J = 8.5 Hz, 2H), 7.53 (dd, J = 7.4, 1.3 Hz, 1H), 7.49 (td, J = 7.3, 1.1 Hz, 1H), 7.22 (s, 1H), 1.39 (s, 9H). **^{13}C NMR** (125.7 MHz, CD_2Cl_2) δ 152.53, 141.39, 135.98, 133.23, 132.21, 131.98, 130.91, 129.25, 127.79, 125.85, 122.82, 121.85, 121.38, 119.37, 34.59, 30.97. **MP:** 160 - 163 °C. **MS (MALDI-TOF):** m/z = 404.4 $[M]^+$. **UV-Vis:** λ_{max} (nm) (ϵ ($dm^3.mol^{-1}.cm^{-1}$)) = 463 ($1.26x10^4$), 438 ($1.70x10^4$), 322 ($2.26x10^4$), 292 ($2.38x10^4$), 267 ($2.24x10^4$).

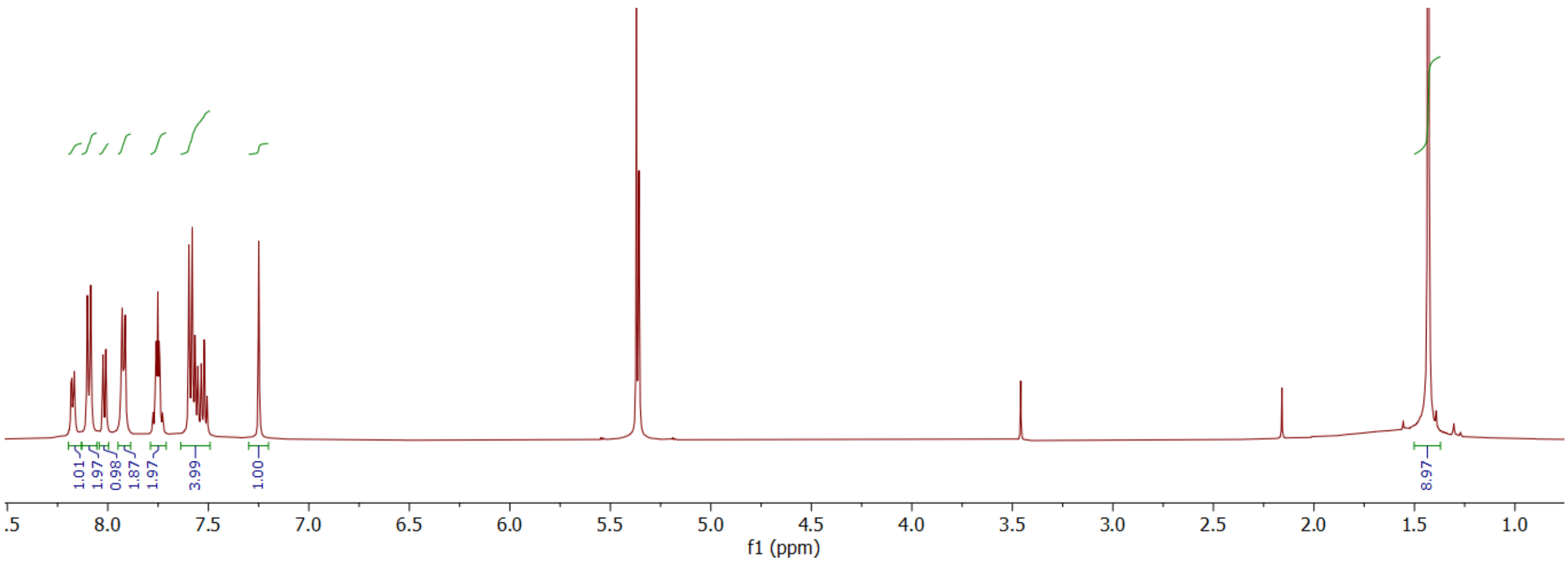
1.01
1.97
0.98
1.87
1.97
3.99
1.00
8.97
.5 8.0 7.5 7.0 6.5 6.0 5.5 5.0 4.5 4.0 3.5 3.0 2.5 2.0 1.5 1.0
f1 (ppm)

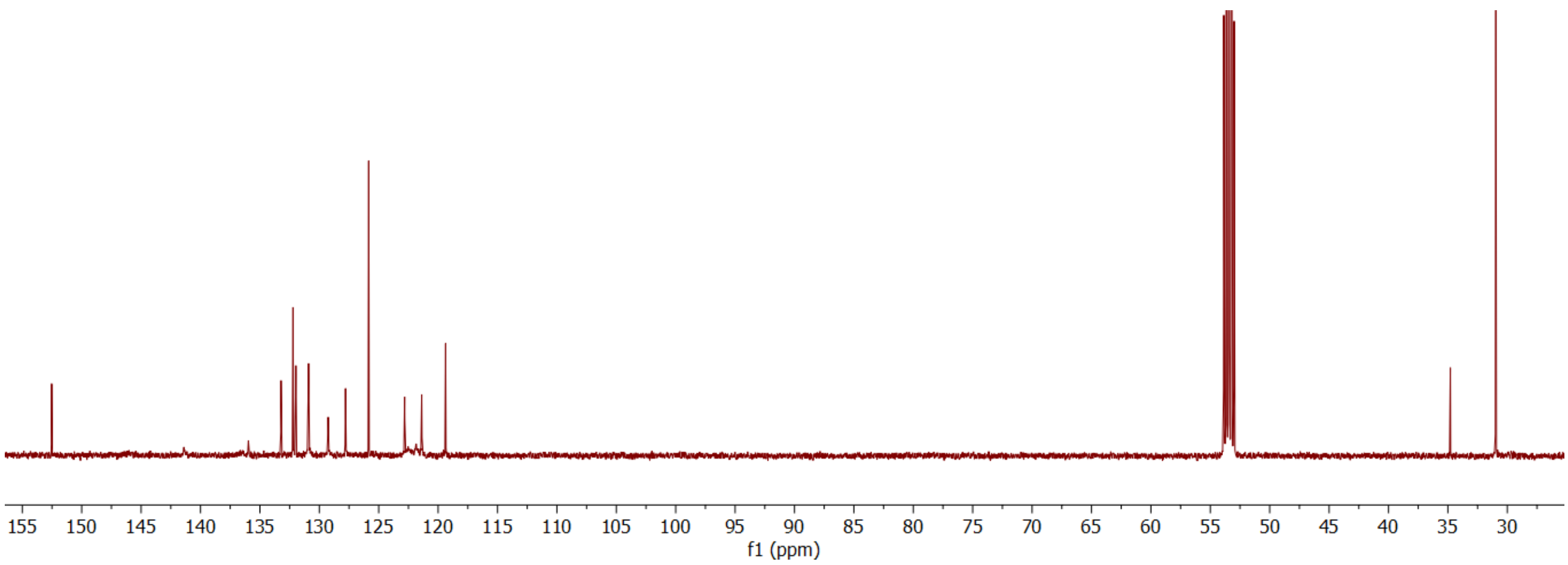
155 150 145 140 135 130 125 120 115 110 105 100 95 90 85 80 75 70 65 60 55 50 45 40 35 30
f1 (ppm)

AB condensation product (7c)

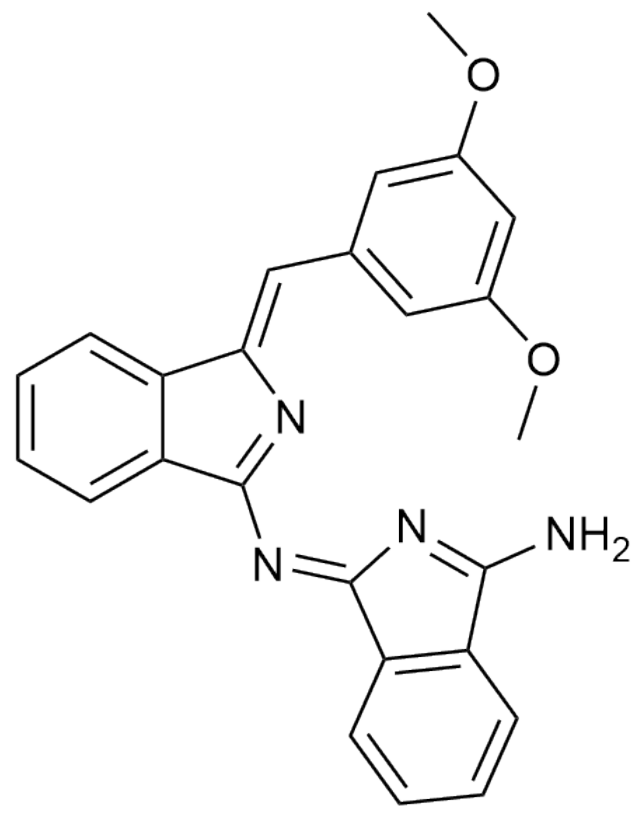

To a solution of **1c**[7b] (1.0 g, 3.6 mmol) in MeOH (20 mL) was added 1-imino-3,3-dimethoxyisoindoline **6a** (0.685 g, 3.6 mmol). The solution was stirred at 50°C overnight. Upon completion the precipitate was filtered off and purified by column chromatography eluting with distilled THF/DCM (1:10). The orange second fraction was collected, the solvents were removed and recrystallised from MeOH to yield **7c** as an orange solid (0.25 g, 17 %).

^{1}H NMR (400 MHz, $CDCl_3$) δ 8.64 (s, 1H), 8.12 (s, 1H), 7.99 (d, J = 7.3 Hz, 1H), 7.82 (dd, J = 6.4, 1.2 Hz, 1H), 7.70 – 7.64 (m, 2H), 7.53 – 7.43 (m, 2H), 7.06 (s, 1H), 6.51 (t, J = 2.3 Hz, 1H), 3.91 (s, 6H). **^{13}C NMR** (101 MHz, $CDCl_3$) δ 160.90, 137.60, 132.19, 131.90, 129.50, 128.10, 123.03, 121.79, 119.54, 109.03, 55.65. **MP:** 163-165 °C **MS (MALDI-TOF):** m/z = 409.16 $[M]^+$ **UV-Vis:** λ_{max} (nm) (ϵ ($dm^3.mol^{-1}.cm^{-1}$)) = 427 ($4.0x10^4$), 329 ($5.0x10^4$), 263 ($1.0x10^5$).

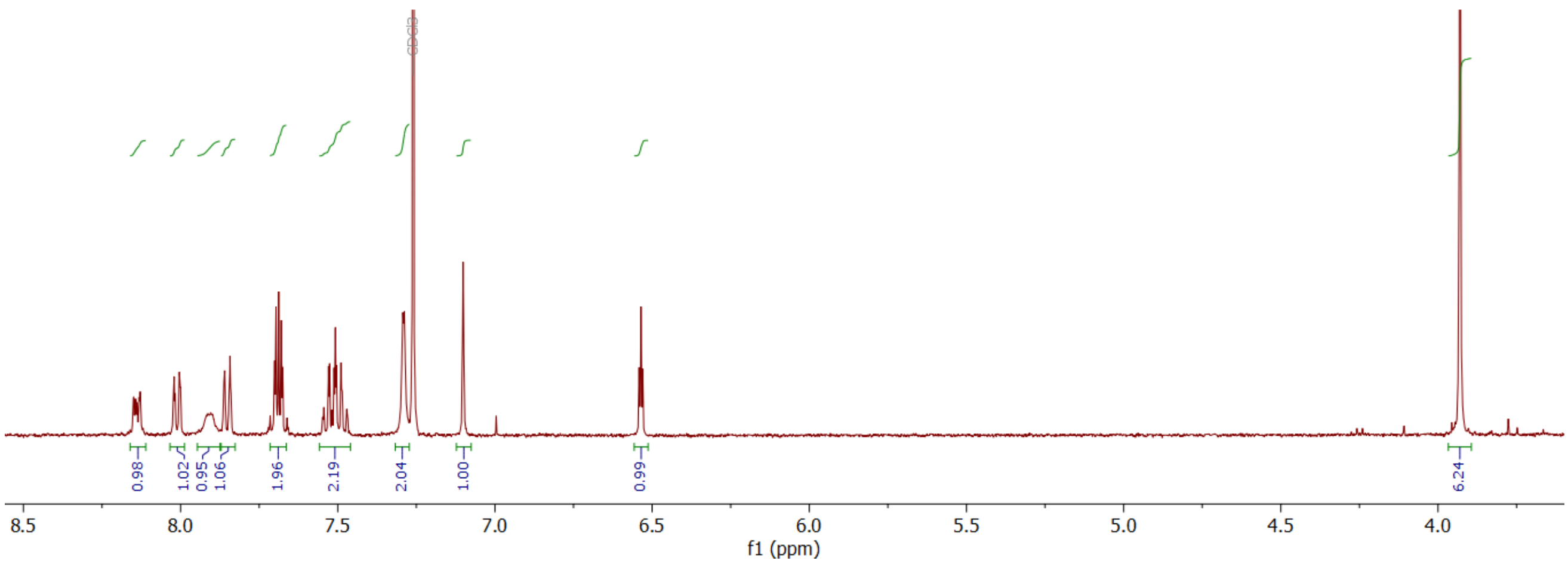

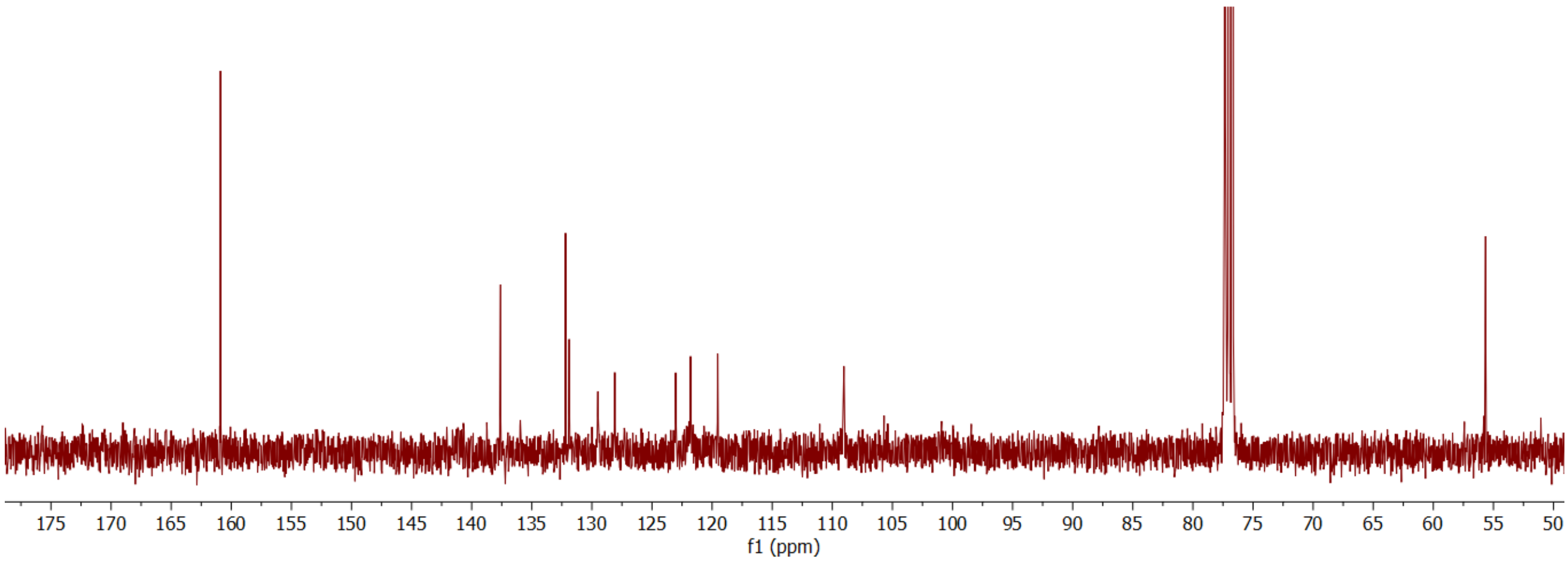

AB condensation product (12)

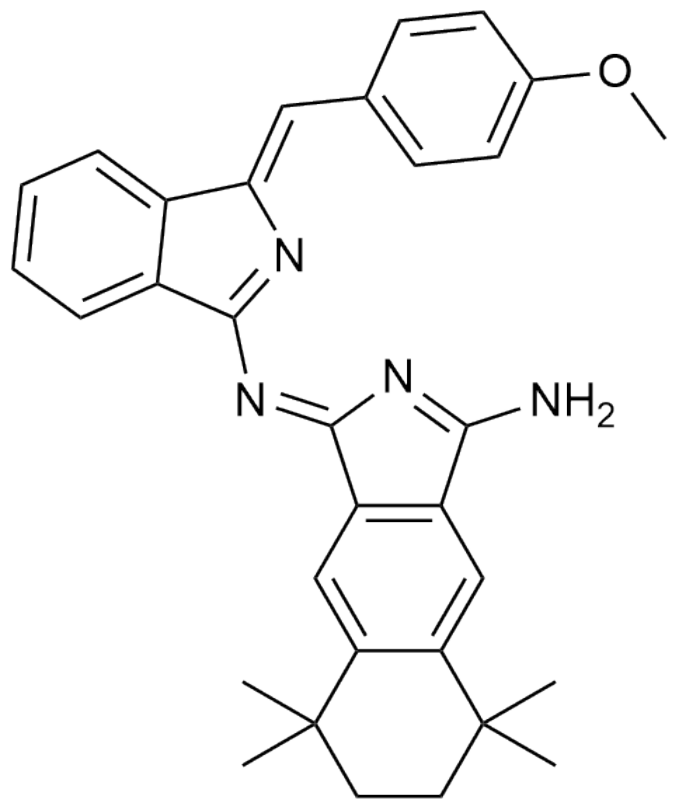

A mixture of **1a**[7] (0.25 g, 1 mmol), dimethoxyisoindoline **10** (0.30 g, 1 mmol) and oven dried 4Å molecular sieves was heated to 40°C overnight. Upon completion the dark red solution was decanted and the sieves rinsed several times with DCM. The organics were combined and the solvent removed. The crude solid was recrystallised from EtOAc to yield **12** as an orange solid (0.24 g, 48 %).

^{1}H NMR (500 MHz, $CDCl_3$) δ 8.08 – 7.99 (m, 3H), 7.98 – 7.90 (d, J = 7.5 Hz, 1H), 7.82 (s, 1H), 7.77 (d, J = 7.4 Hz, 1H), 7.40 (m, 2H), 7.17 (s, 1H), 6.98 (d, J = 8.8 Hz, 2H), 3.84 (s, 3H), 1.70 (s, 4H), 1.36 (s, 6H), 1.30 (s, 6H). **^{13}C NMR** (125.7 MHz, $CDCl_3$) δ 170.14, 169.13, 160.76, 156.81, 151.85, 151.11, 145.69, 141.80, 135.73, 134.09, 133.18, 129.00, 128.58, 128.18, 127.40, 125.10, 122.66, 121.61, 121.51, 119.13, 114.60, 55.49, 35.49, 35.43, 34.69, 34.61, 31.83. **MP:** 148 - 154 °C. **MS (MALDI-TOF):** m/z = 488.65 $[M]^{+}$. **UV-Vis:** λ_{max} (nm) (ϵ ($dm^3.mol^{-1}.cm^{-1}$)) = 442 ($6.88x10^3$), 334 ($1.19x10^4$), 260 ($1.68x10^4$).

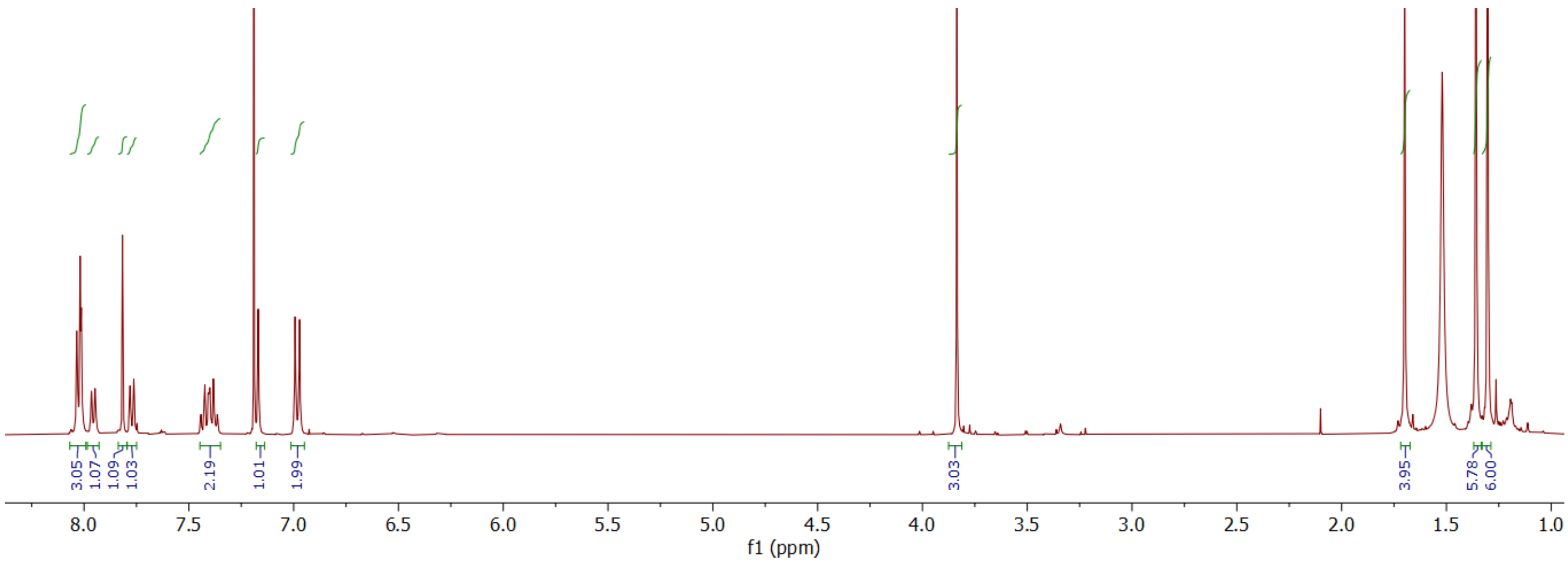
3.05
1.07
1.09
1.03
2.19
1.01
1.99
3.03
3.95
5.78
6.00
8.0 7.5 7.0 6.5 6.0 5.5 5.0 4.5 4.0 3.5 3.0 2.5 2.0 1.5 1.0
f1 (ppm)

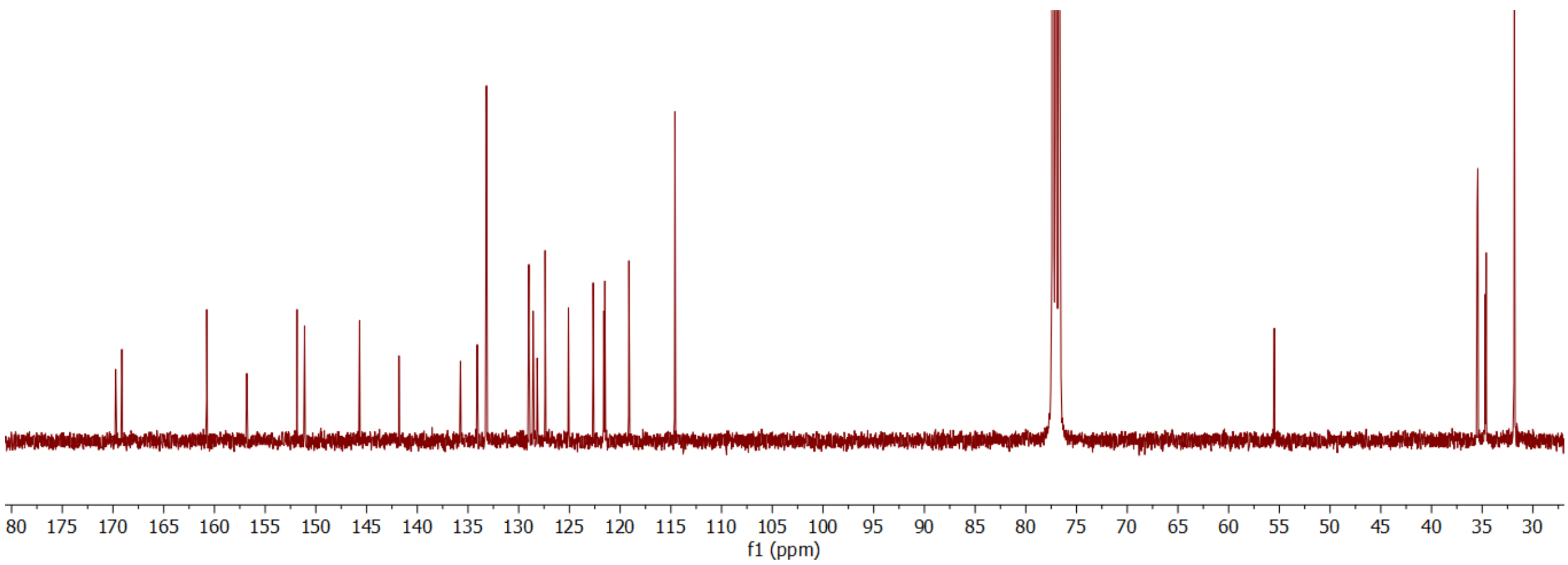
80 175 170 165 160 155 150 145 140 135 130 125 120 115 110 105 100 95 90 85 80 75 70 65 60 55 50 45 40 35 30
f1 (ppm)

AB condensation product (16)

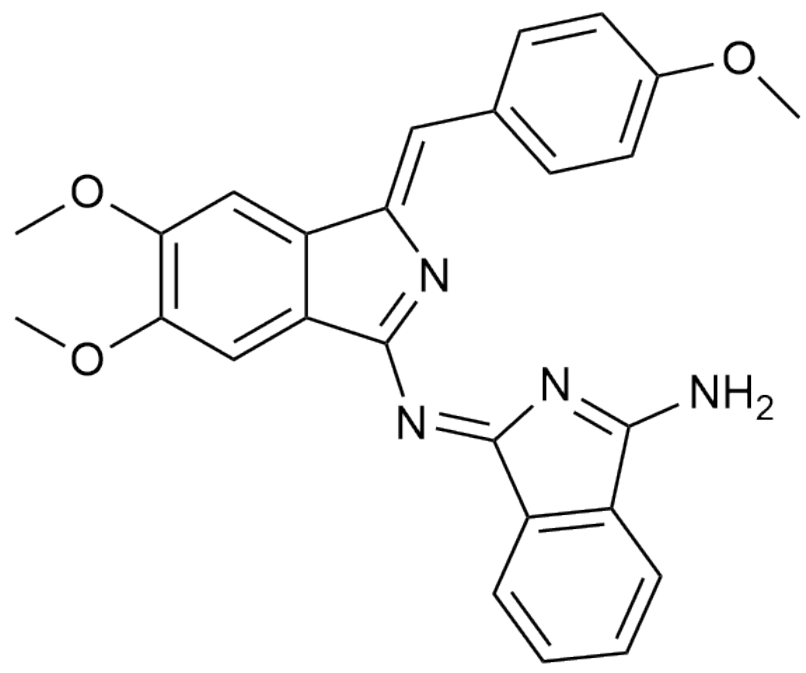

To a solution of **1d**[9] (0.31 g, 1 mmol) and 1-imino-3,3-dimethoxyisoindoline **6a** (0.19 g, 1 mmol) in dry DCM (10 mL) was added oven dried 4Å molecular sieves. The solution was heated to reflux overnight. Upon cooling the dark red solution was decanted and the molecular sieves washed several times with DCM. The organics were combined, the solvent removed, and the crude product recrystallised from EtOAc to yield **16** as an orange solid (0.19 g, 43 %).

^{1}H NMR (500 MHz, $CDCl_3$) (peaks for major stereoisomer) δ 8.35 – 8.31 (m, 1H), 7.98 – 7.93 (m, 3H), 7.69 – 7.63 (m, 3H), 7.45 (s, 1H), 7.16 (s, 1H), 7.03 (d, J = 8.8 Hz, 2H), 4.16 (s, 3H), 3.94 (s, 3H), 3.89 (s, 3H). **^{13}C NMR** (125.7 MHz, $CDCl_3$) δ 163.33, 157.30, 151.84, 136.92, 135.63, 134.65, 133.84, 133.43, 132.86, 128.81, 128.24, 125.98, 125.88, 124.45, 118.44, 117.77, 115.57, 115.32, 103.65, 101.60, 56.89, 56.49, 55.64. **MP:** 195 - 199°C. **MS (MALDI-TOF):** m/z = 437.28 $[M]^+$. **UV-Vis:** λ_{max} (nm) (ϵ ($dm^3.mol^{-1}.cm^{-1}$)) = 437 ($5.96x10^3$), 330 ($1.02x10^4$), 263 ($1.37x10^4$)

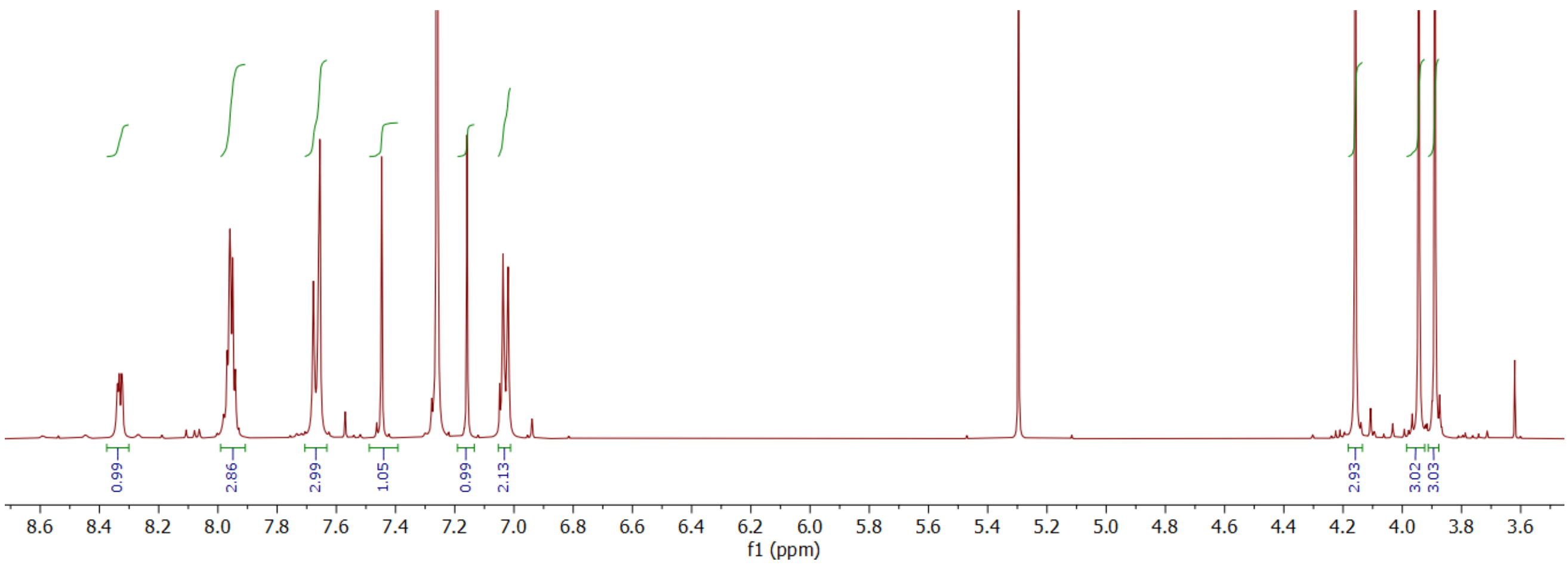

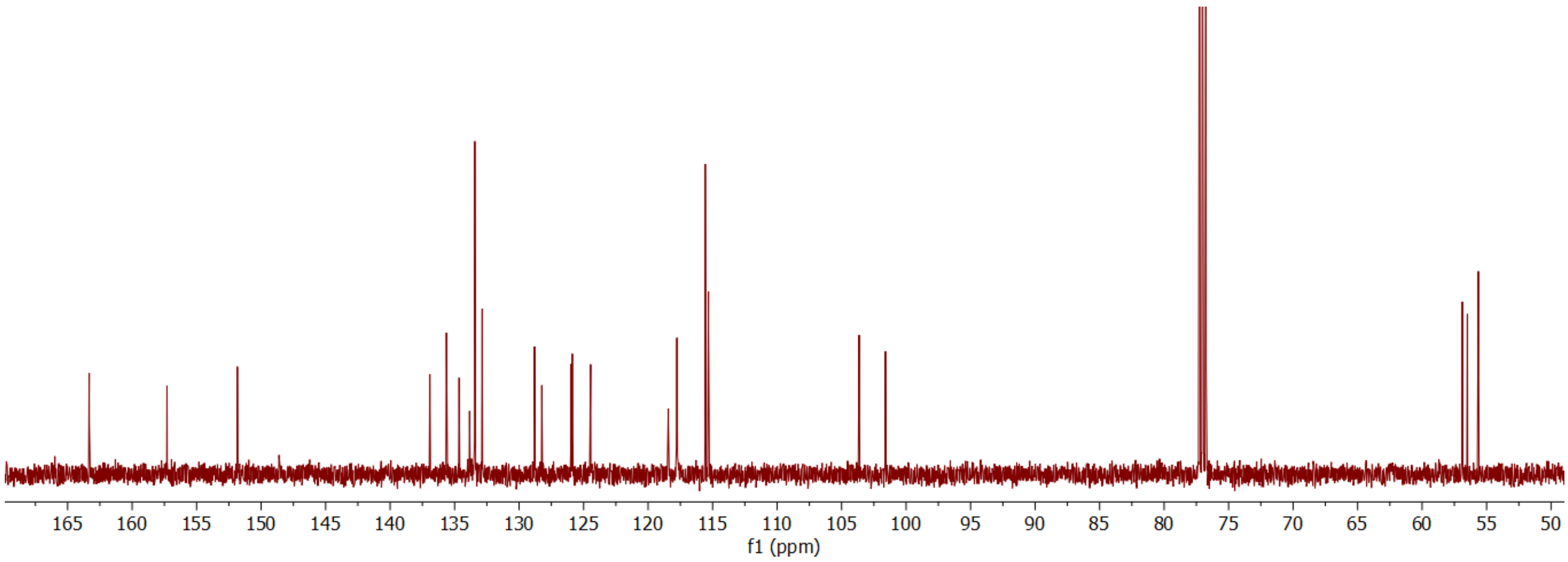

AB condensation product (15)

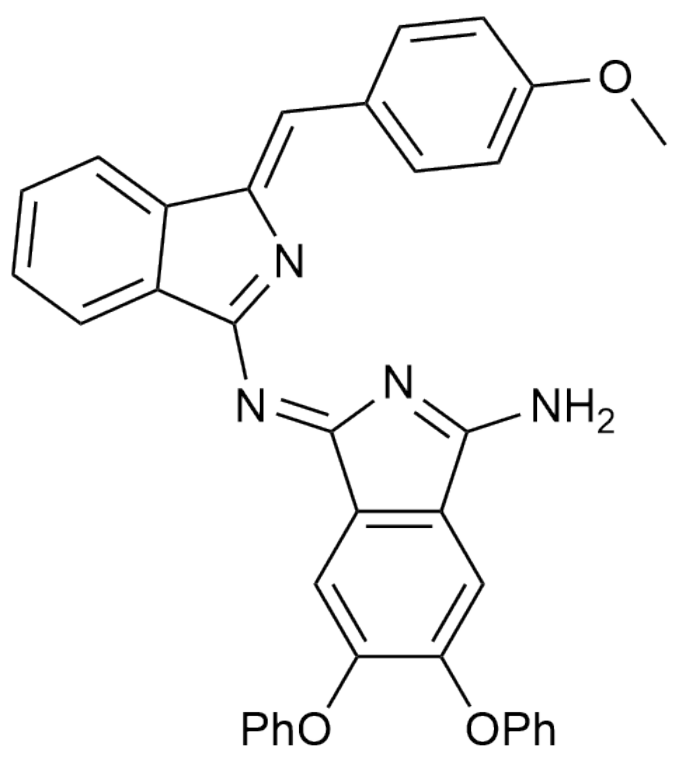

To a solution of **1a**[7] (0.25 g, 1 mmol) and 4,5-diphenoxy-phthalonitrile[20] (0.31 g, 1 mmol) in dry MeOH (10 mL) was added NaOMe (65 mg, 1.2 mmol). The solution was stirred at RT overnight. Once complete the precipitate was filtered and washed with cold methanol to yield **15** as an orange solid (0.25 g, 44 %).

^{1}H NMR (500 MHz, DMSO-d_6) (peaks for major stereoisomer) δ 9.07 (br s, 2H), 8.24 (d, J = 6.8 Hz, 2H), 7.95 (d, J = 7.6 Hz, 1H), 7.78 (s, 1H), 7.50 – 7.28 (m, 8H), 7.25 – 7.08 (m, 7H), 7.00 (d, J = 8.9 Hz, 2H), 3.82 (s, 3H). **^{13}C NMR** (125.7 MHz, DMSO-d_6) δ 160.11, 156.95, 156.43, 150.98, 142.37, 133.49, 130.68, 130.63, 129.27, 128.77, 127.46, 124.65, 124.23, 121.86, 119.81, 119.00, 118.33, 114.78, 114.55, 113.86, 55.69. **MP:** 173 - 178°C. **MS (MALDI-TOF):** m/z = 563.71 $[M]^+$. **UV-Vis:** λ_{max} (nm) (ϵ ($dm^3.mol^{-1}.cm^{-1}$)) = 446 ($6.34x10^3$), 336 ($1.13x10^4$), 262 ($2.41x10^4$).

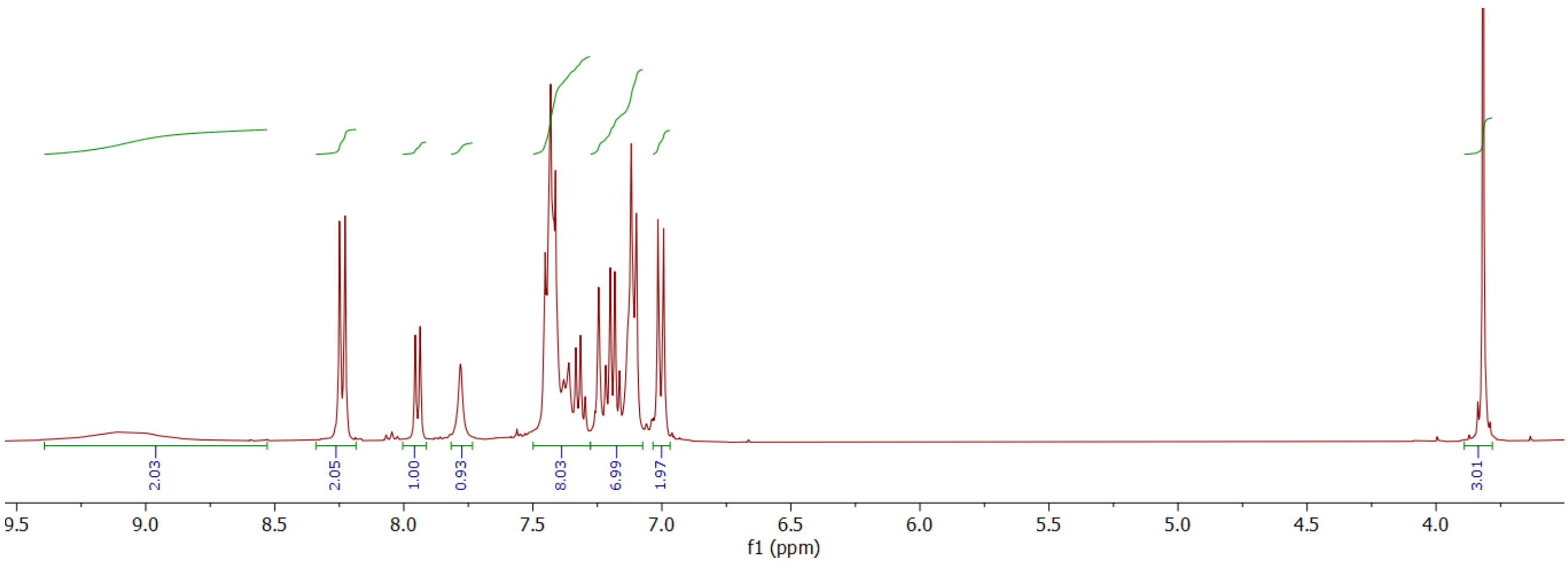
2.03
2.05
1.00
0.93
8.03
6.99
1.97
3.01
9.5
9.0
8.5
8.0
7.5
7.0
6.5
6.0
5.5
5.0
4.5
4.0
f1 (ppm)

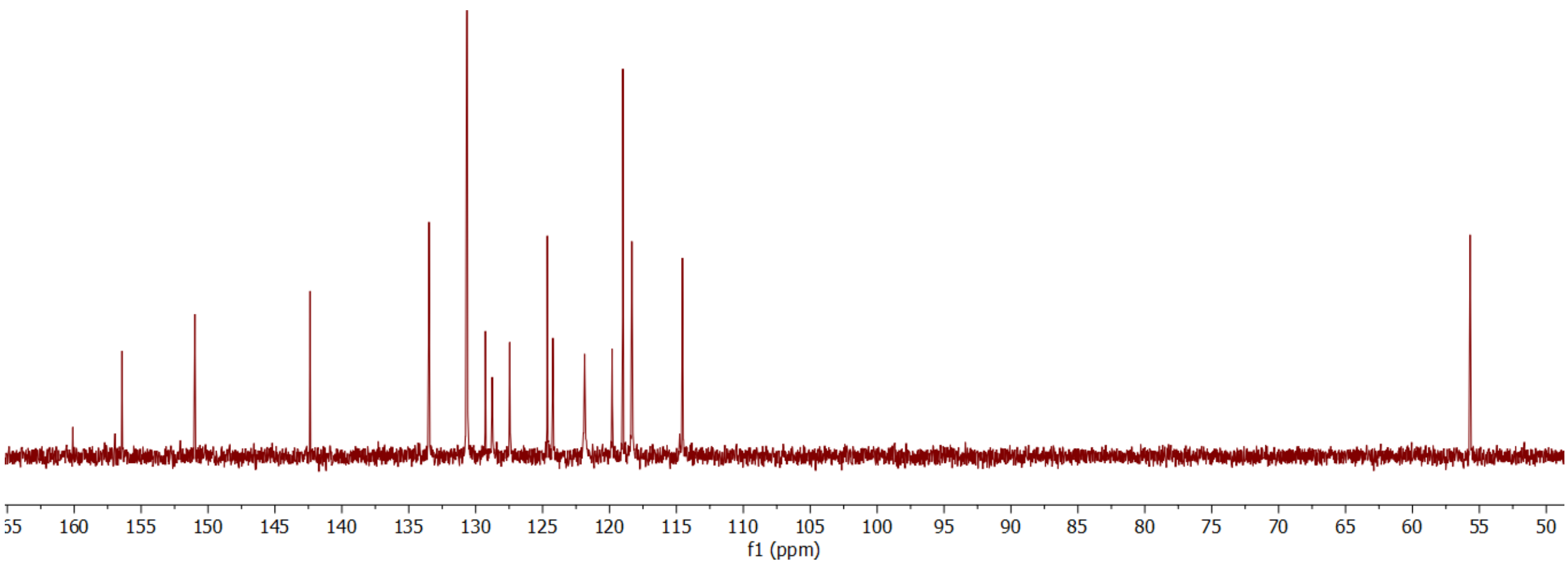
160
155
150
145
140
135
130
125
120
115
110
105
100
95
90
85
80
75
70
65
60
55
50
f1 (ppm)

NBoc protected condensation product (14)

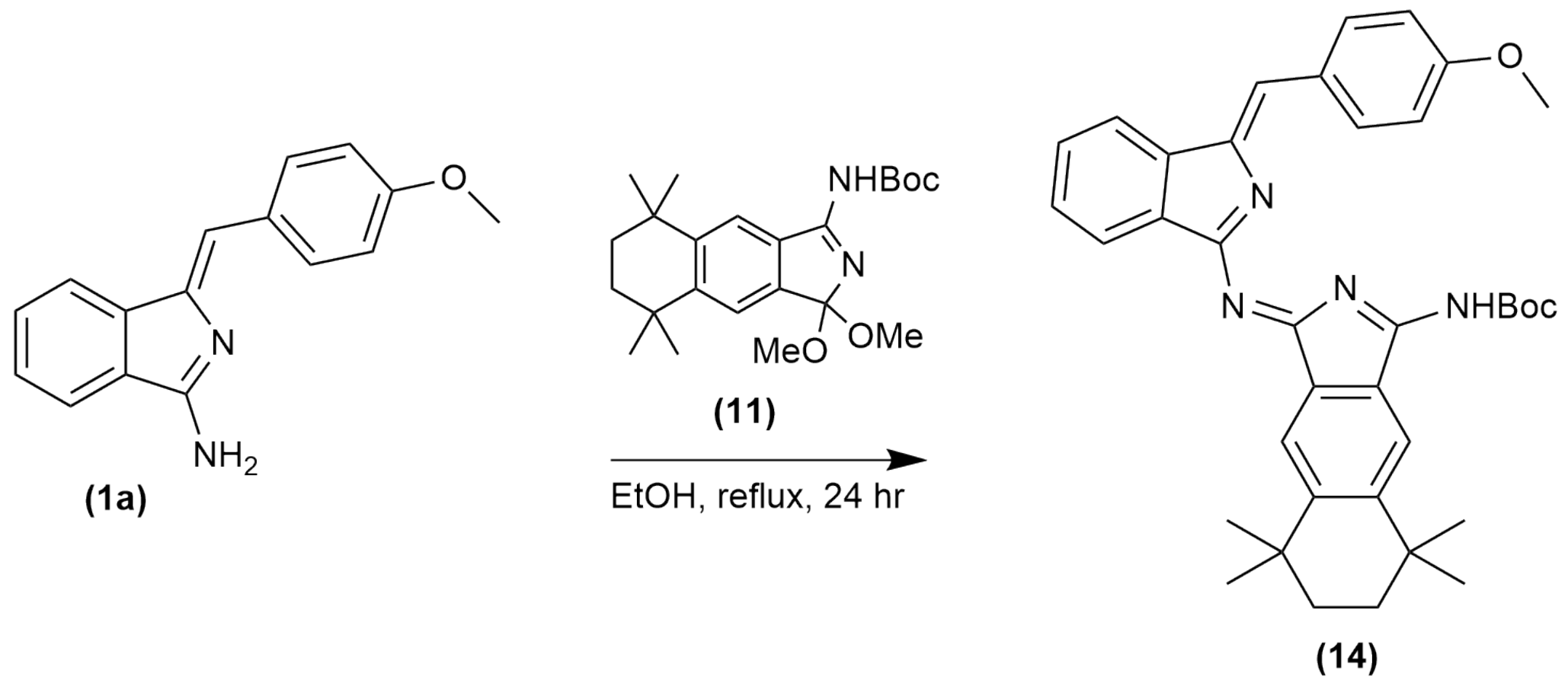

A solution of **1a**[7] (0.16 g, 0.64 mmol) and **11** (0.24 g, 0.64 mmol) in EtOH (10 mL) was heated to reflux overnight. The solution was cooled to RT and the solvent removed. The crude residue was subject to silica gel chromatography eluting with DCM/PE (3:2). The dark red fraction was collected and the solvent removed to yield **14** as a dark red solid (0.24 g, 64 %).

^{1}H NMR (500 MHz, $CDCl_3$) δ 8.35 (d, J = 8.4 Hz, 2H), 8.14 (s, 1H), 8.09 (s, 1H), 8.04 (d, J = 7.4 Hz, 1H), 7.94 (s, 1H), 7.90 – 7.83 (m, 1H), 7.54 – 7.43 (m, 1H), 7.31 (s, 1H), 7.14 (d, J = 8.4 Hz, 2H), 3.90 (s, 3H), 1.78 (s, 4H), 1.60 (s, 9H), 1.38 (s, 12H). **^{13}C NMR** (125.7 MHz, $CDCl_3$) 170.97, 170.35, 162.47, 155.34, 153.05, 152.30, 147.54, 143.19, 136.90, 135.43, 134.12, 131.21, 130.15, 130.06, 129.33, 126.24, 123.79, 122.75, 122.19, 120.27, 115.18, 81.00, 55.19, 37.21, 36.47, 33.61, 28.60. **MP:** 128 - 134 °C. **MS (MALDI-TOF):** m/z = 589.24 $[M]^{+}$. **FT-IR:** (ATR), ν (cm^{-1}): 1782 (C=O).

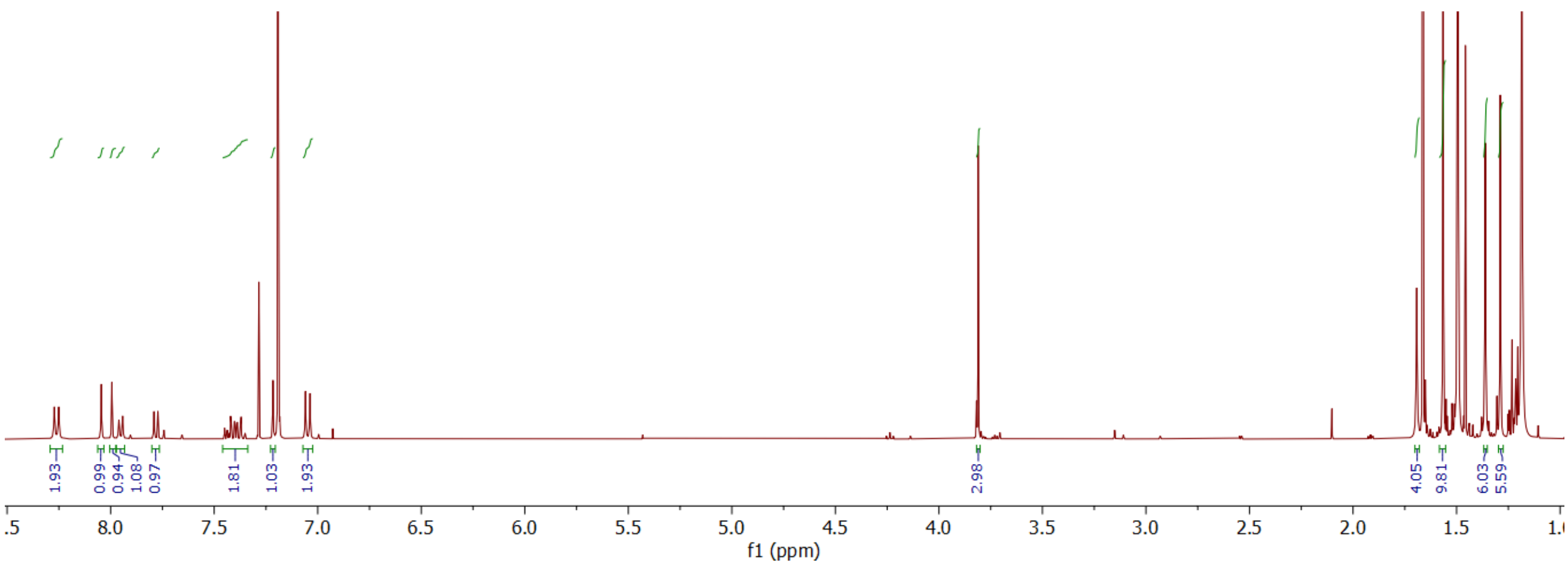

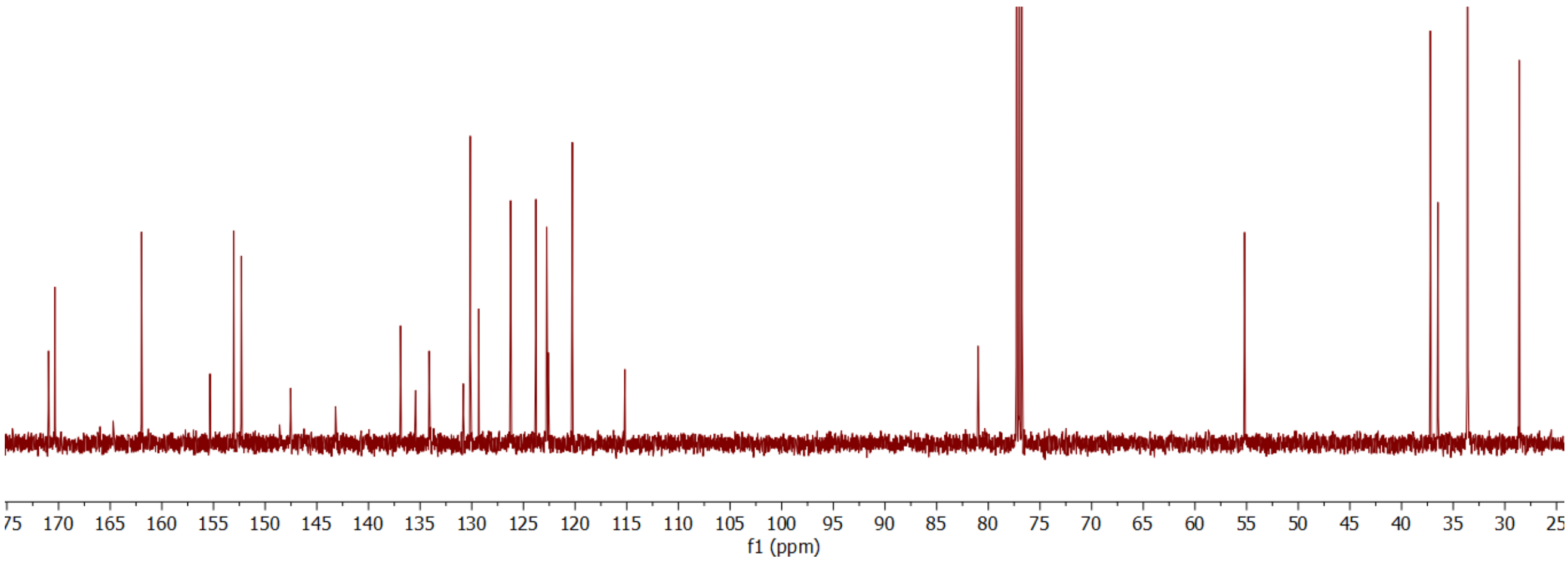

Deprotection of (14)

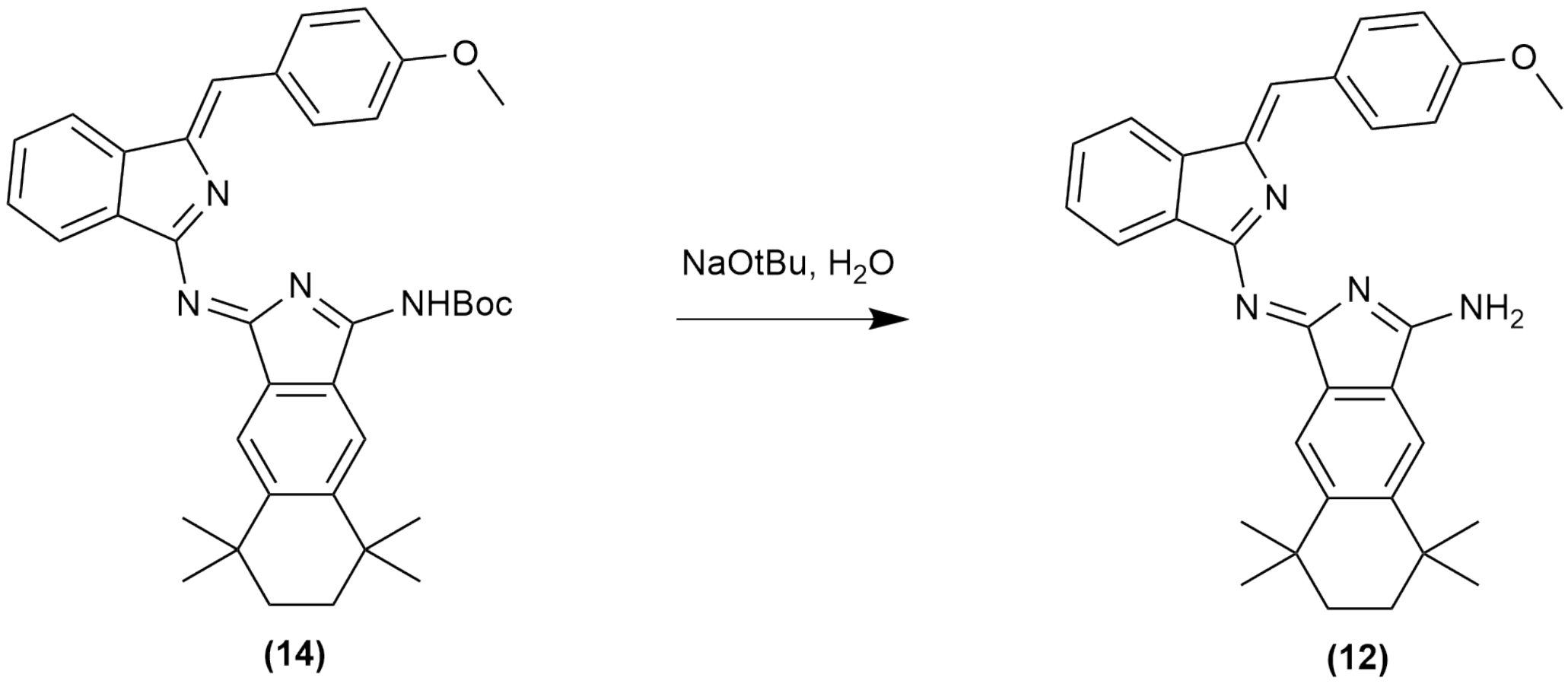

To a solution of NBoc protected AB **14** (1 mmol, 0.59 g) in THF (5 mL) was added NaO-*t*-Bu (3 eq, 0.29 g) and H_2O (1 eq, 0.02 mL).[14] The solution was heated to reflux for 6 hrs, cooled, and recrystallised from DCM/hexane to yield **12** as an orange solid (0.32 g, 65 %).

AB condensation product (22a)

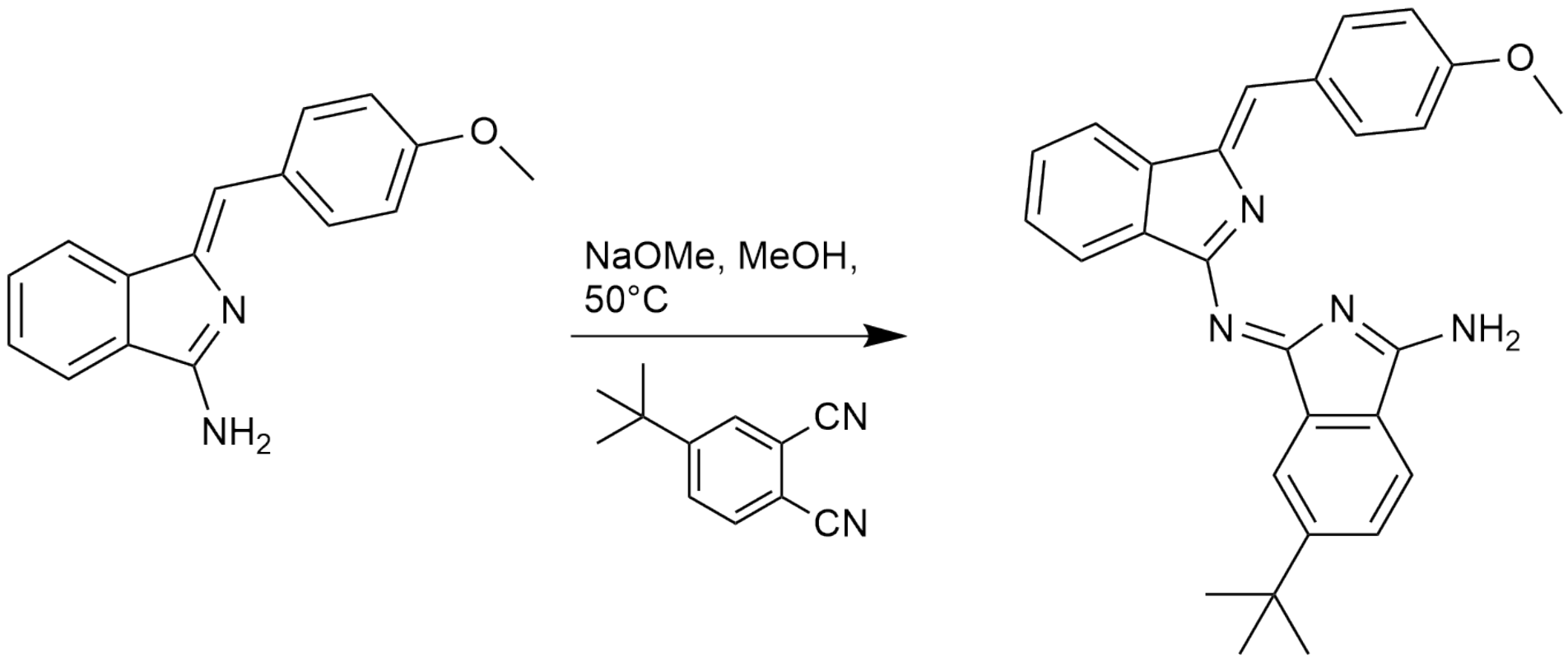

A solution of **1a**[7] (0.3 g, 1.2 mmol), 4-*tert*-butyl-phthalonitrile (0.27 g, 1.44 mmol), and NaOMe (0.01 g, 1.8 mmol) was heated to 50°C in methanol (20 mL) overnight. Upon cooling to RT the precipitated product was filtered and washed with cold methanol to yield **22a** as an orange material that was poorly soluble (0.31 g, 59 %).

^{1}**H NMR** (400 MHz, DMSO-d_6) (peaks for major stereoisomer) δ 8.89 (s, 2H), 8.25 (d, J = 8.6 Hz, 2H), 7.96 (d, J = 7.5 Hz, 1H), 7.89 (d, J = 11.9 Hz, 2H), 7.74 (s, 1H), 7.44 (d, J = 7.6 Hz, 1H), 7.32 (s, 2H), 7.25 (s, 1H), 7.00 (d, J = 8.4 Hz, 2H), 3.81 (s, 3H), 1.38 (s, 9H). **MP:** 249°C. **MS (MALDI-TOF):** m/z = 434.21 $[M]^+$. **UV-Vis:** λ_{max} (nm) (ϵ ($dm^3.mol^{-1}.cm^{-1}$)) = 441 ($1.5x10^3$), 274 ($1.05x10^4$).

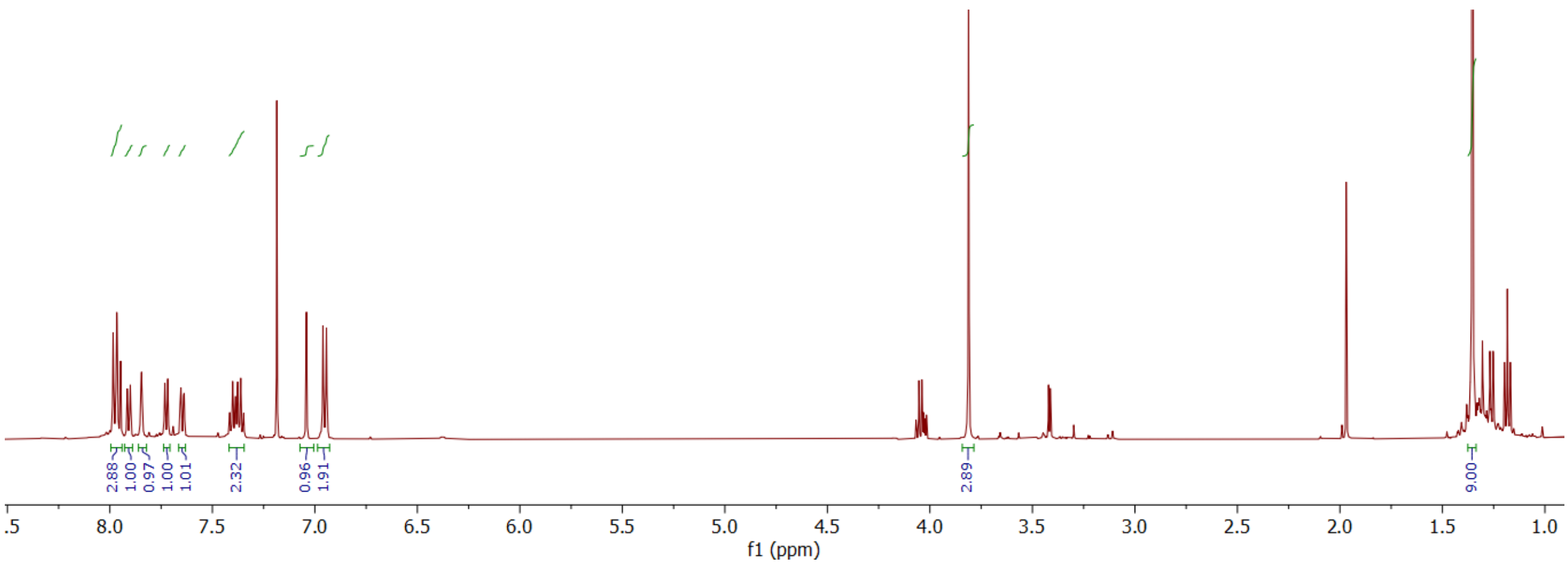

AB condensation product (22b)

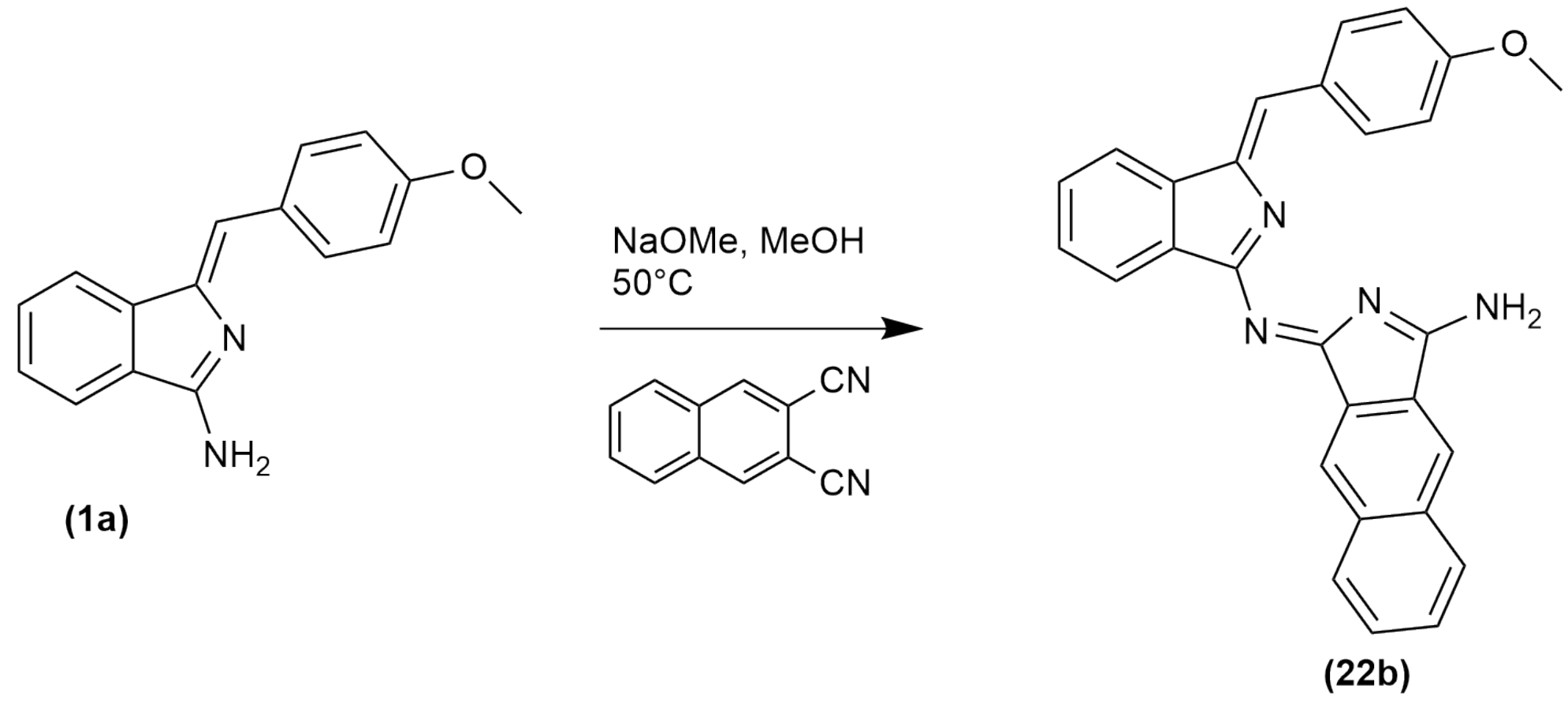

A solution of **1a**[7] (0.3 g, 1.2 mmol), naphthalonitrile (0.26 g, 1.44 mmol), and NaOMe (0.01 g, 1.8 mmol) was heated to 50°C in methanol (20 mL) overnight. Upon cooling to RT the precipitated product was filtered and washed with cold methanol to yield **22b** as a dark orange material (0.35 g, 68 %).

^{1}H NMR (400 MHz, DMSO-d_6) (peaks for major stereoisomer) δ 8.95 (s, 2H), 8.48 (d, J = 14.7 Hz, 2H), 8.30 – 8.21 (m, 3H), 8.14 – 8.08 (m, 1H), 7.96 (d, J = 7.6 Hz, 1H), 7.77 – 7.68 (m, 2H), 7.44 (t, J = 7.4 Hz, 1H), 7.37 (d, J = 7.6 Hz, 1H), 7.32 (t, J = 7.3 Hz, 1H), 7.24 (s, 1H), 7.00 (d, J = 9.1 Hz, 2H), 3.81 (s, 3H). **^{13}C NMR** (101 MHz, DMSO-d_6) δ 159.55, 146.35, 142.03, 134.28, 133.92, 133.82, 132.98, 131.69, 129.92, 129.68, 128.92, 128.26, 128.09, 127.00, 122.08, 121.51, 121.40, 120.84, 119.33, 114.07, 55.20. **MP:** 221°C. **MS (MALDI-TOF):** m/z = 428.16 $[M]^+$.

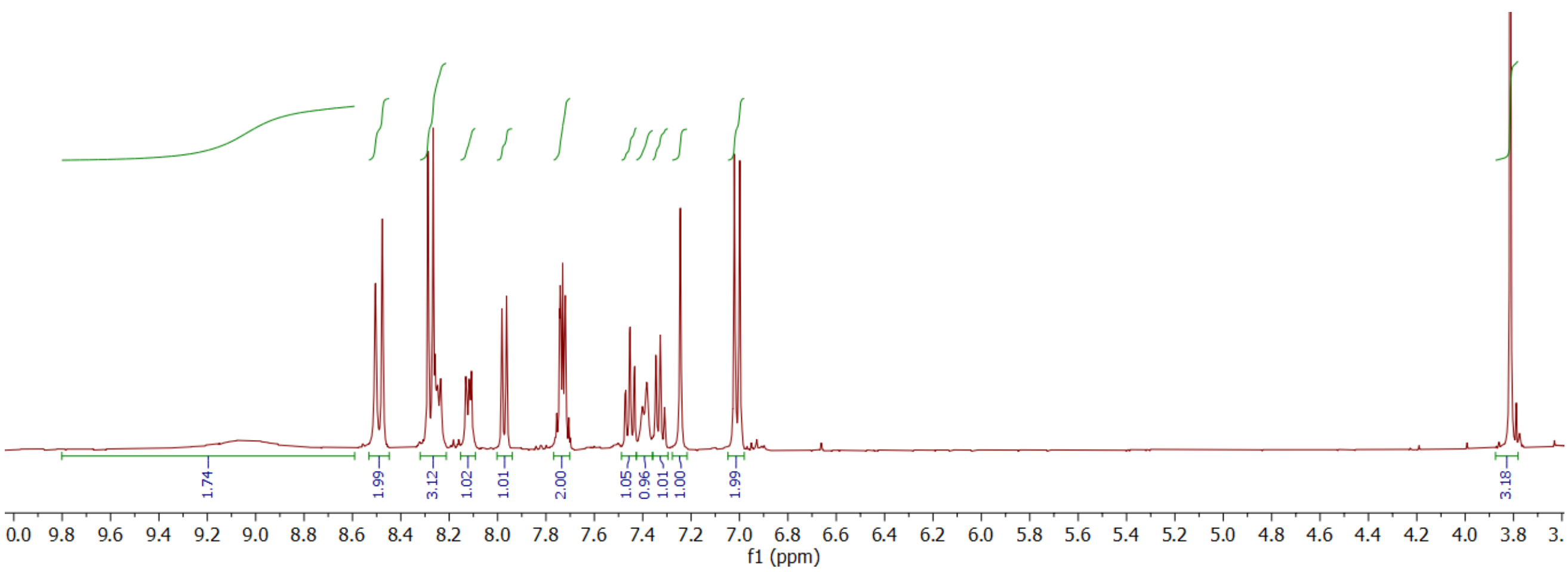

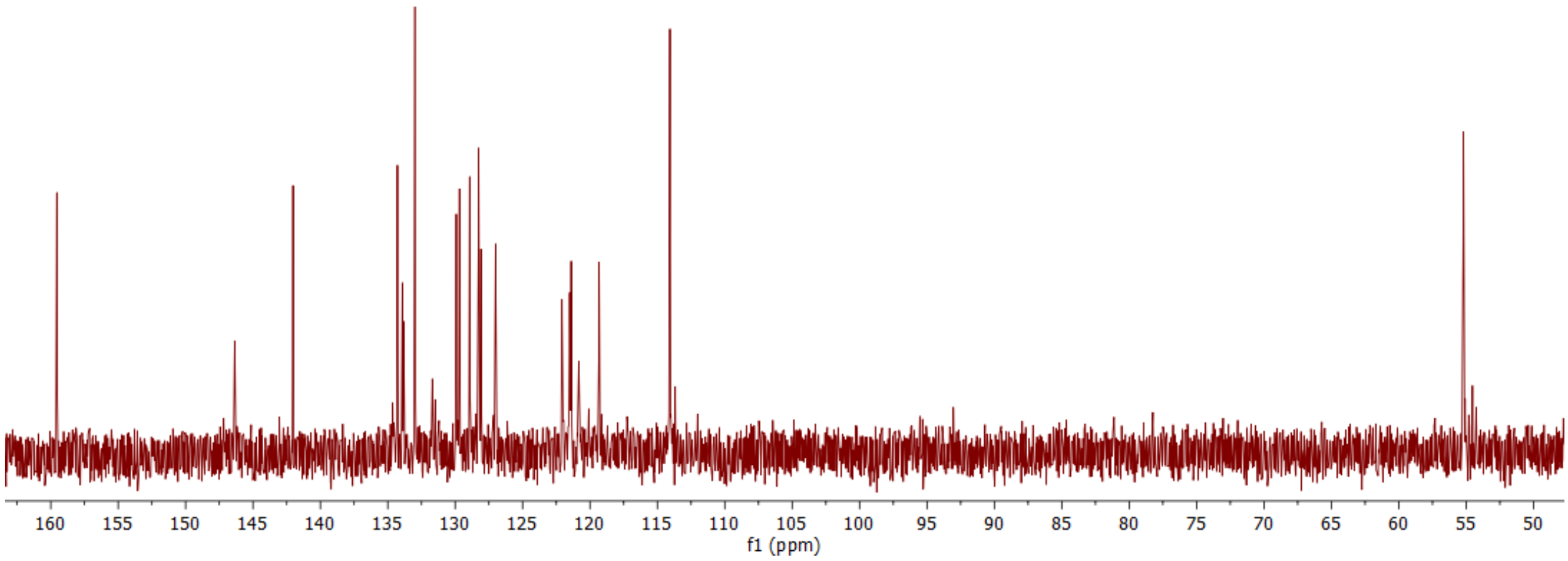

ABA Trimers (tribenzodiazatripyrrins)

ABA trimer (8b)

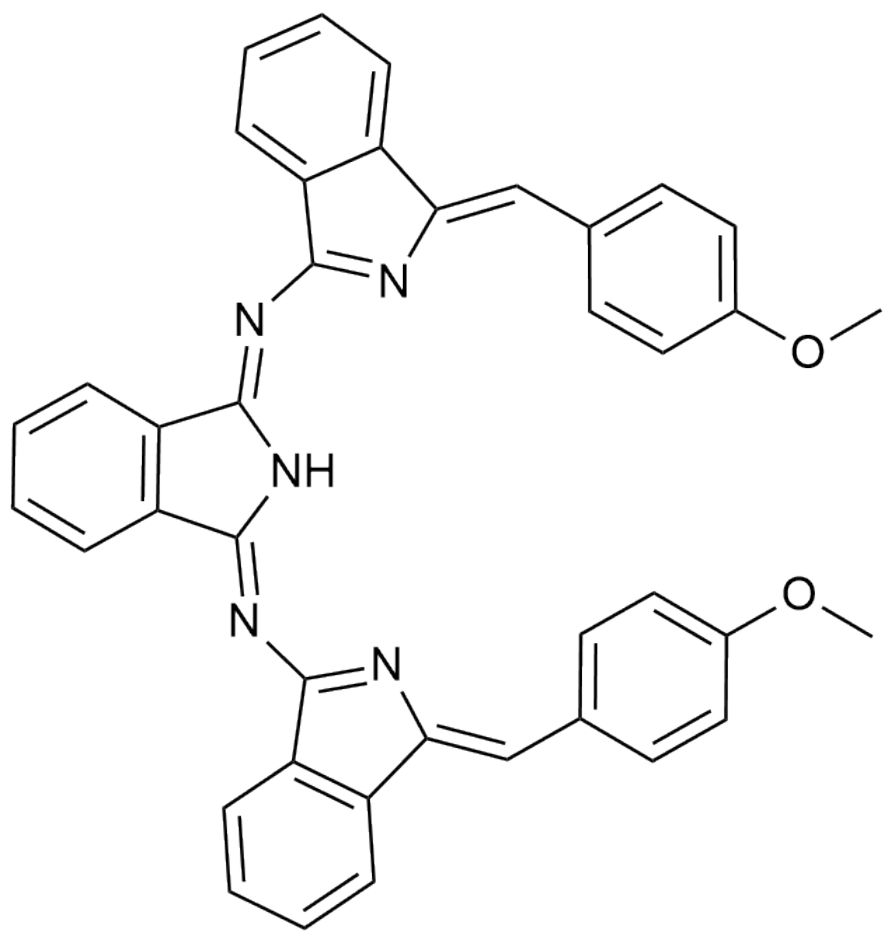

A solution of **7a** (0.20 g, 0.53 mmol) in toluene (10 mL) was heated to 100°C overnight. Upon cooling the solvent was removed and the crude residue purified by silica gel chromatography eluting with EtOAc/hexane (2:3). The first brown fraction was collected, the solvent removed, and the residue recrystallised from DCM/hexane to yield **8b** as a dark brown solid (65 mg, 40 %).

^{1}H NMR (500 MHz, $CDCl_3$) δ 8.22 – 8.14 (m, 2H), 8.00 – 7.95 (m, 2H), 7.95 – 7.89 (m, 4H), 7.74 – 7.66 (m, 4H), 7.55 – 7.41 (m, 4H), 6.62 (s, 2H), 6.40 (d, J = 8.3 Hz, 4H), 3.43 (s, 6H). **^{13}C NMR** (125.7 MHz, $CDCl_3$) δ 169.17, 159.85, 156.68, 145.77, 142.41, 135.33, 134.33, 133.20, 132.30, 128.79, 128.60, 127.08, 126.17, 123.24, 121.17, 118.82, 113.54, 54.93. **MP:** 200 - 203°C. **MS (MALDI-TOF):** m/z = 610.78 $[M]^+$. **UV-Vis:** λ_{max} (nm) (ϵ ($dm^3.mol^{-1}.cm^{-1}$)) = 475 (4.93x10^3), 400 (9.57x10^3), 347 (1.29x10^4), 264 (2.01x10^4).

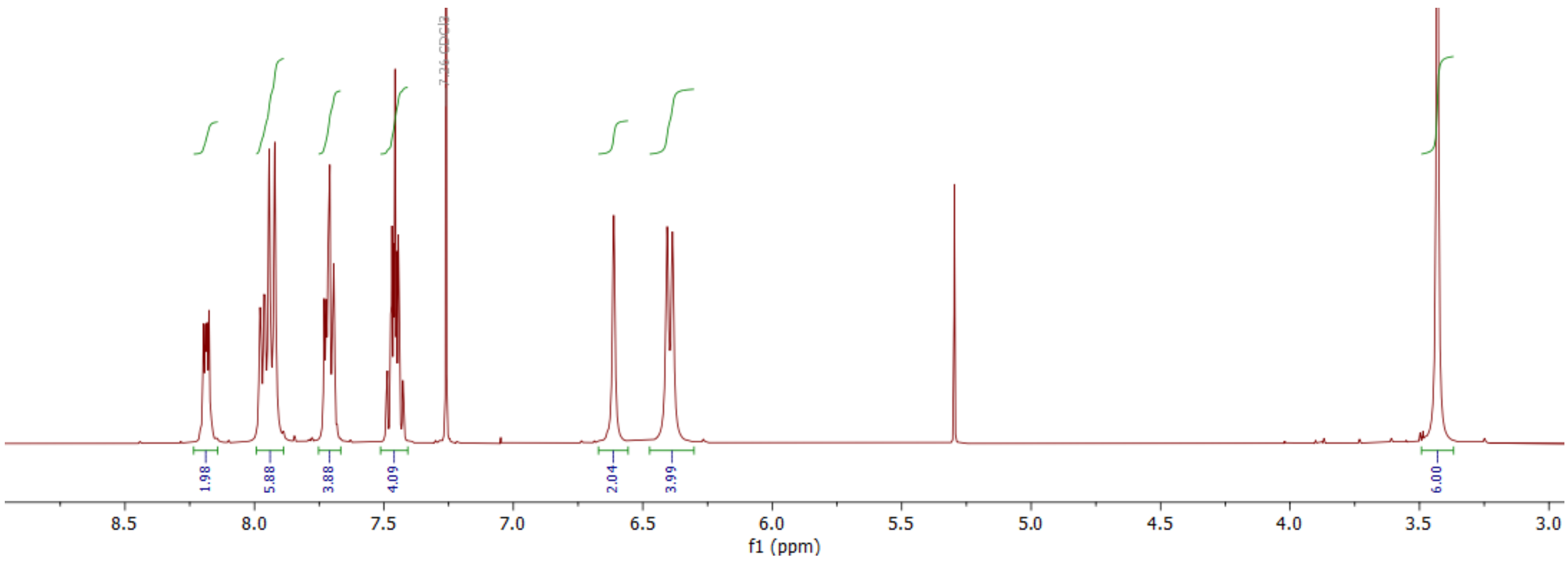
7.26 CDCl3
1.98
5.88
3.88
4.09
2.04
3.99
6.00
f1 (ppm)

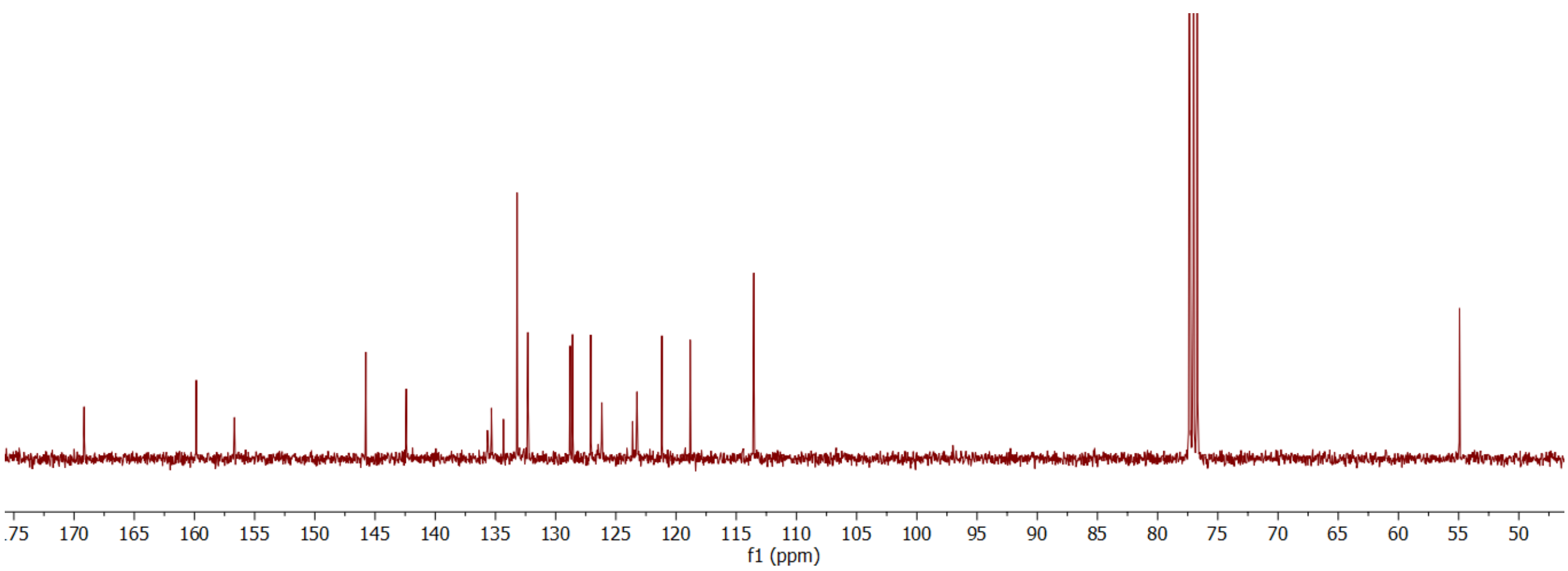
f1 (ppm)

ABA trimer (8c)

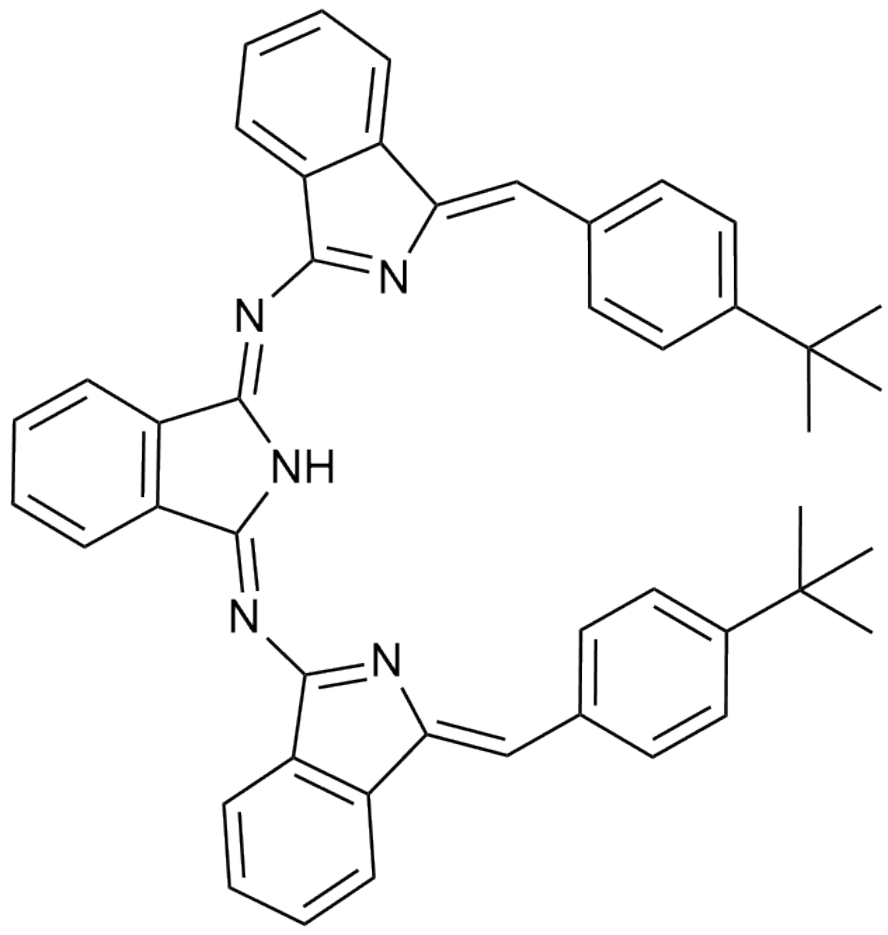

ABA trimeric product **8c** was isolated from the reaction synthesising **7b** (trace) and in the synthesis of TBTAP **2c** from **7b**.

^{1}H NMR (400 MHz, $CDCl_3$) (peaks for major stereoisomer) δ 13.85 (s, 1H), 8.23 – 8.18 (m, 2H), 8.02 – 7.98 (m, 2H), 7.91 (d, J = 8.2 Hz, 4H), 7.75 – 7.71 (m, 4H), 7.51 – 7.47 (m, 4H), 6.94 (d, J = 8.0 Hz, 4H), 6.69 (s, 2H), 1.14 (s, 18H). **^{13}C NMR** (101 MHz, $CDCl_3$) δ 169.56, 156.58, 151.43, 146.83, 142.27, 135.78, 132.90, 132.38, 131.19, 128.71, 127.39, 126.72, 125.93, 125.14, 123.29, 121.28, 119.16, 30.86. **MP:** 194 - 200°C. **MS (MALDI-TOF):** m/z = 664.34 $[M]^{+}$. **UV-Vis:** λ_{max} (nm) (ϵ ($dm^3.mol^{-1}.cm^{-1}$)) = 463 ($1.01x10^4$), 397 ($2.09x10^4$), 341 ($3.6x10^4$), 271 ($4.76x10^4$).

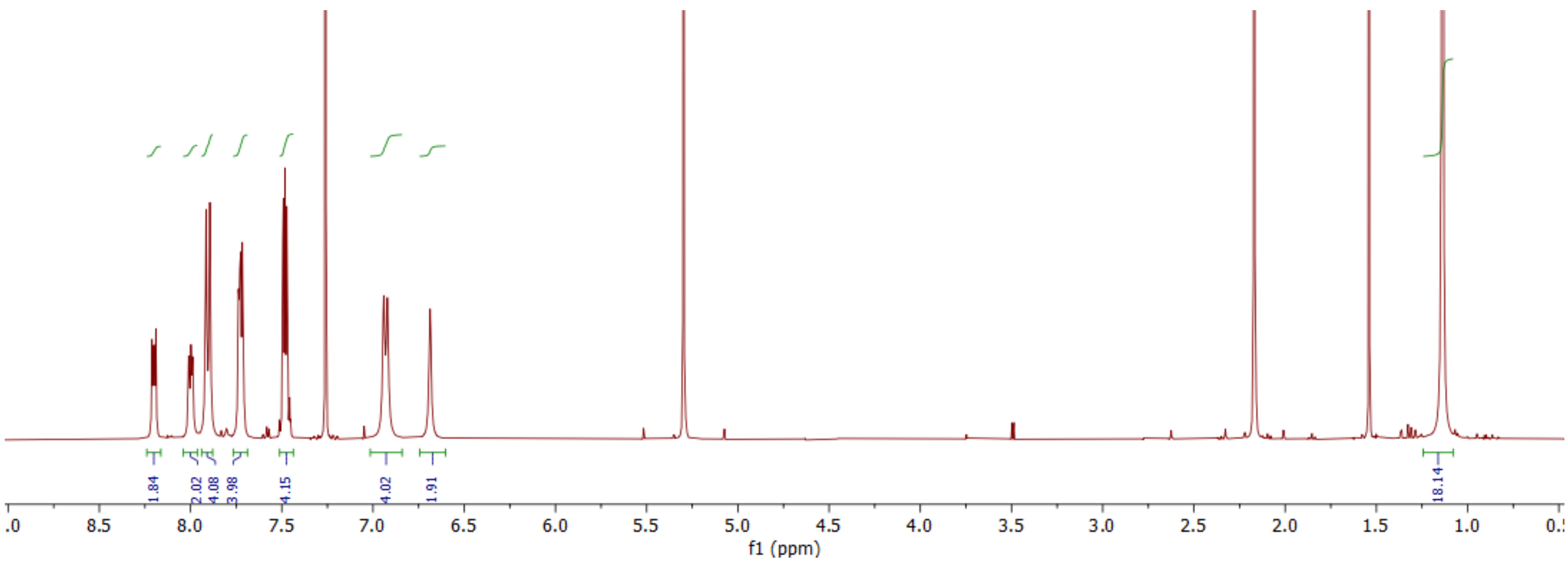

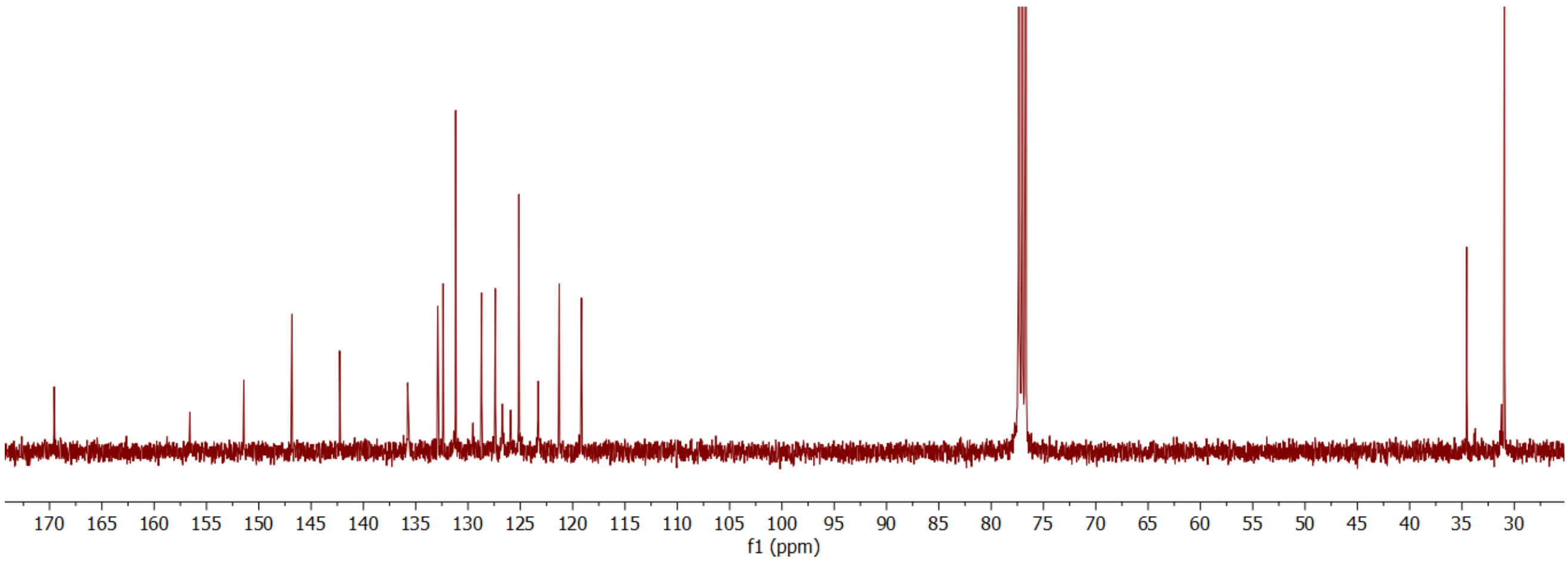

ABA trimer (8d)

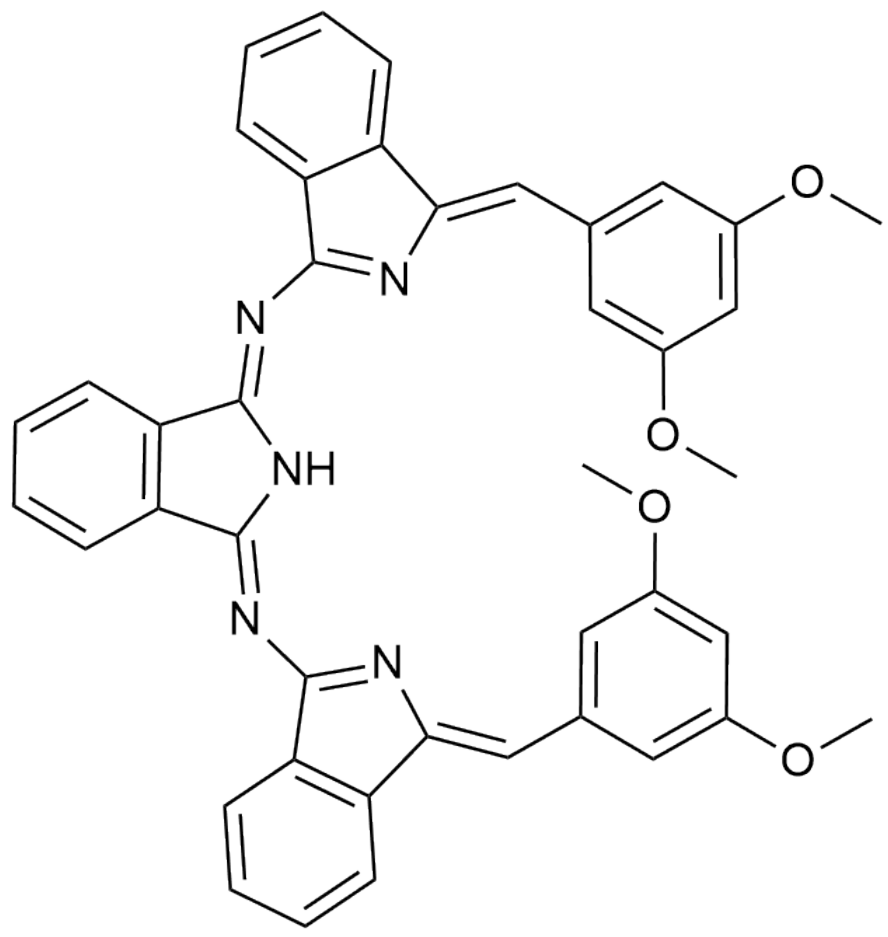

ABA trimeric product **8d** was isolated from the reaction synthesising **7c** (trace) and in the synthesis of TBTAP **2d** from **7c**.

^{1}H NMR (400 MHz, $CDCl_3$) (peaks for major stereoisomer) δ 14.30 (s, 1H), 8.21 – 8.15 (m, 2H), 7.97 – 7.91 (m, 2H), 7.76 – 7.68 (m, 4H), 7.50 – 7.43 (m, 4H), 7.15 (s, 4H), 6.51 (s, 2H), 6.13 (t, J = 2.3 Hz, 2H), 3.39 (s, 12H). **^{13}C NMR** (101 MHz, $CDCl_3$) δ 170.22, 160.19, 157.07, 147.58, 142.71, 137.26, 135.69, 132.46, 128.80, 127.42, 126.08, 123.34, 121.11, 119.07, 109.31, 100.97, 54.71, 53.43 **MP:** 180 - 190 °C. **MS (MALDI-TOF):** m/z = 672.76 $[M]^{+}$. **UV-Vis:** λ_{max} (nm) (ϵ ($dm^3.mol^{-1}.cm^{-1}$)) = 459 ($2.20x10^4$) 389 ($5.10x10^4$) 338 ($7.38x10^4$) 269 ($7.88x10^4$).

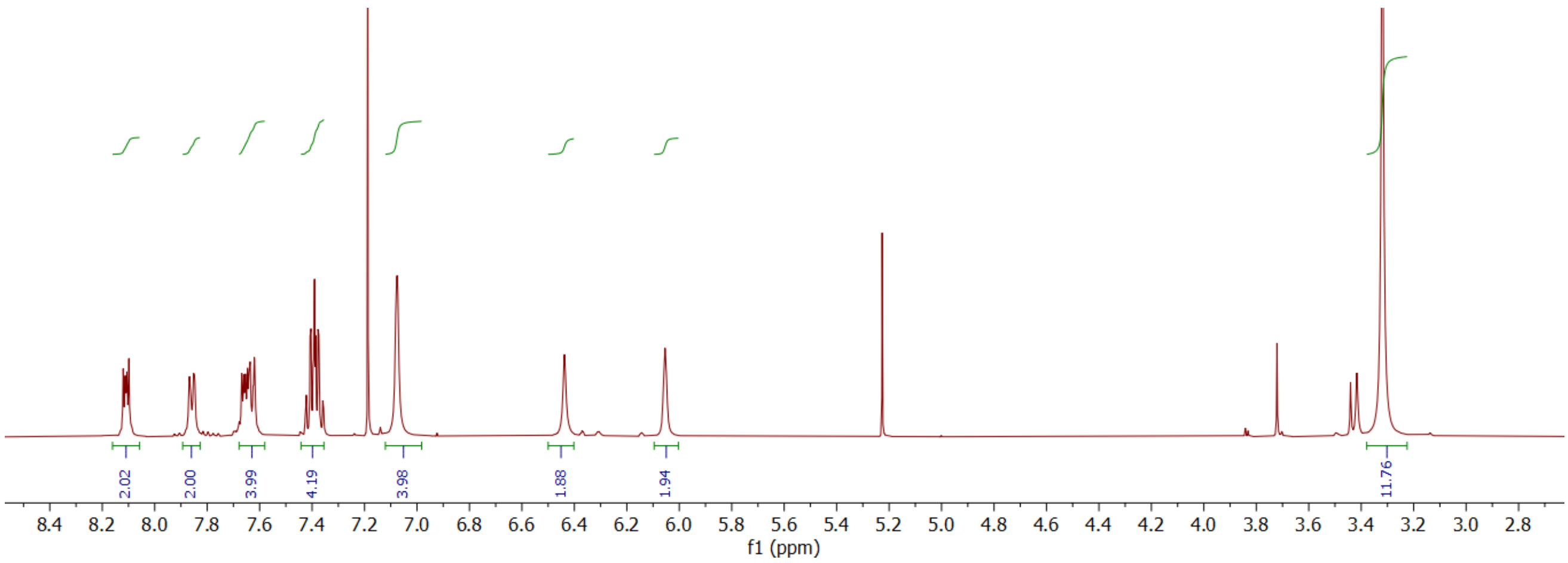
2.02
2.00
3.99
4.19
3.98
1.88
1.94
11.76
8.4 8.2 8.0 7.8 7.6 7.4 7.2 7.0 6.8 6.6 6.4 6.2 6.0 5.8 5.6 5.4 5.2 5.0 4.8 4.6 4.4 4.2 4.0 3.8 3.6 3.4 3.2 3.0 2.8
f1 (ppm)

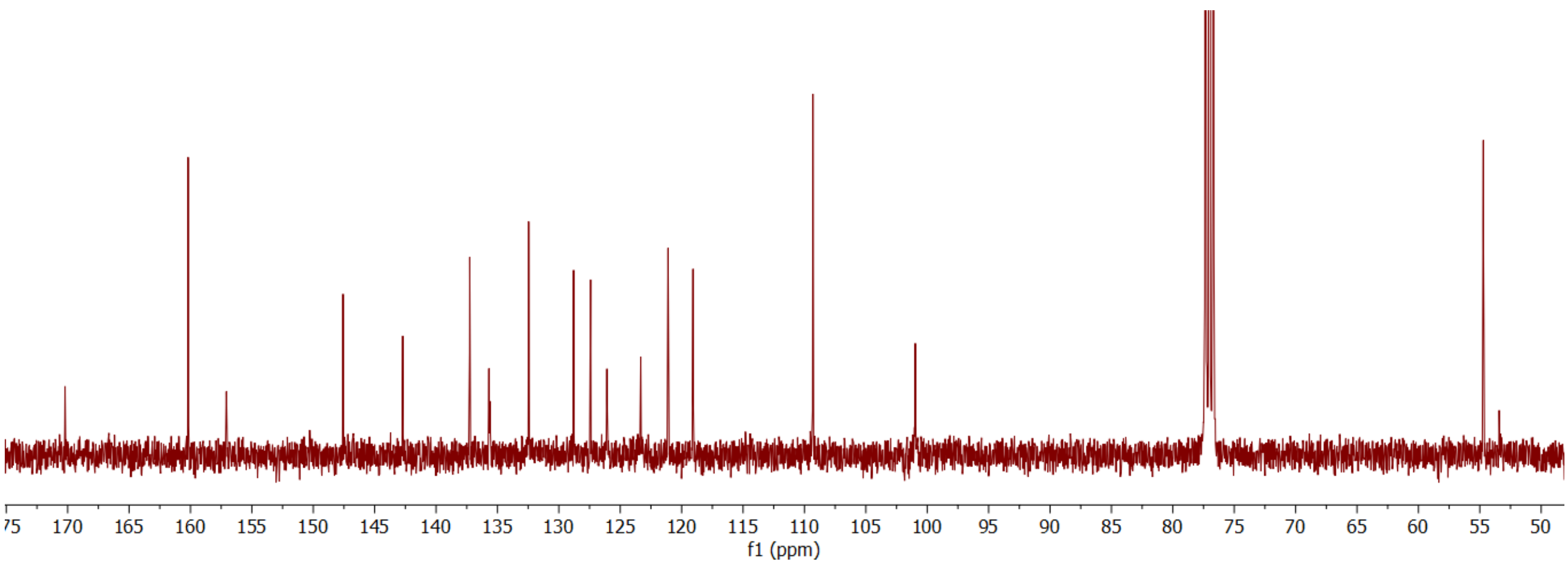
75 170 165 160 155 150 145 140 135 130 125 120 115 110 105 100 95 90 85 80 75 70 65 60 55 50
f1 (ppm)

ABA trimer (13)

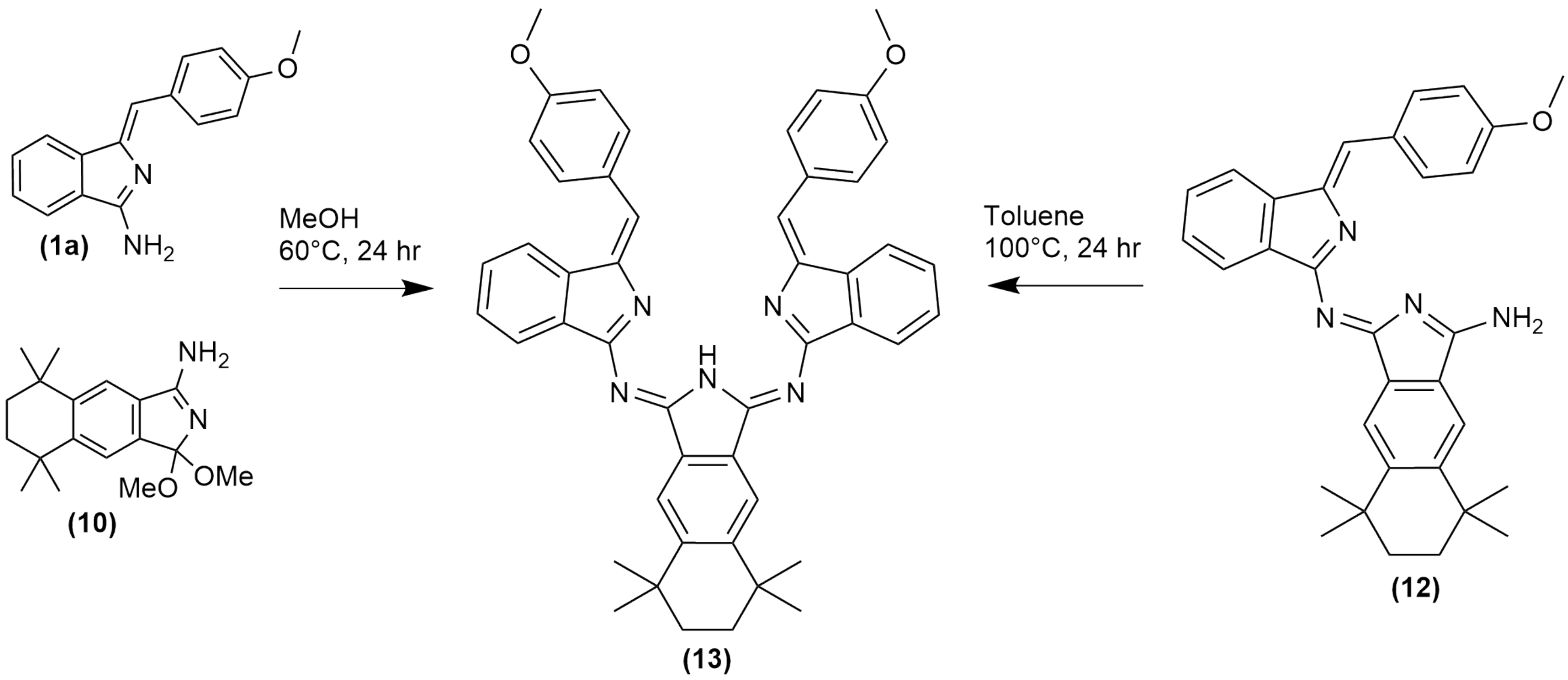

A solution of **1a**[7] (0.5 g, 2 mmol) and **10** (0.30 g, 1 mmol) in MeOH (10 mL) was heated to reflux overnight. Upon cooling the solvent was removed and the crude residue purified by silica gel chromatography eluting with DCM/hexane (3:2). The first brown fraction was collected and recrystallised from DCM/hexane to yield **13** as a dark brown solid (0.52 g, 72 %).

Via (12) A solution of **12** (0.26 g, 0.53 mmol) in toluene (10 mL) was heated to 100°C overnight. Upon cooling the solvent was removed and the crude reside purified by silica gel chromatography eluting with EtOAc/hexane (2:3). The first brown fraction was collected, the solvent removed, and the residue recrystallised from DCM/hexane to yield **13** as a dark brown solid (55 mg, 29 %).

^{1}H NMR (500 MHz, $CDCl_3$) δ 8.12 (s, 2H), 8.00 (d, J = 6.8 Hz, 2H), 7.89 (d, J = 8.3 Hz, 4H), 7.69 (d, J = 6.8 Hz, 2H), 7.47 – 7.41 (m, 4H), 6.58 (s, 2H), 6.36 (d, J = 8.3 Hz, 4H), 3.40 (s, 6H), 1.79 (s, 4H), 1.44 (s, 12H). **^{13}C NMR** (125.7 MHz, $CDCl_3$) δ 169.26, 159.75, 150.60, 145.78, 135.31, 133.15, 128.81, 128.52, 127.00, 125.46, 124.61, 121.73, 121.25, 118.82, 114.52, 113.47, 112.76, 54.90, 35.46, 34.84, 31.97. **MP:** 217 - 220°C. **MS (MALDI-TOF):** m/z = 720.99 $[M]^{+}$. **UV-Vis:** λ_{max} (nm) (ϵ ($dm^3.mol^{-1}.cm^{-1}$)) = 478 ($4.80x10^3$), 402 ($8.87x10^3$), 348 ($1.21x10^4$), 265 ($1.86x10^4$).

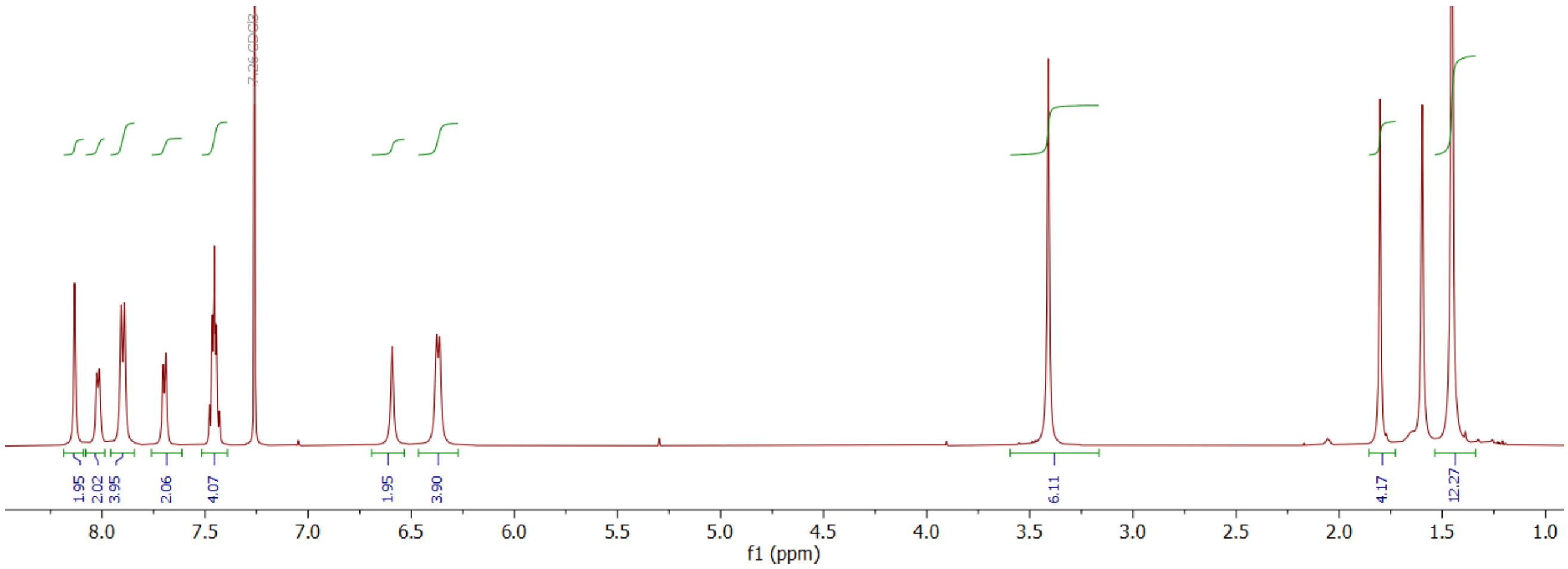
7.26 CDCl3
1.95
2.02
3.95
2.06
4.07
1.95
3.90
6.11
4.17
12.27
8.0
7.5
7.0
6.5
6.0
5.5
5.0
4.5
4.0
3.5
3.0
2.5
2.0
1.5
1.0
f1 (ppm)

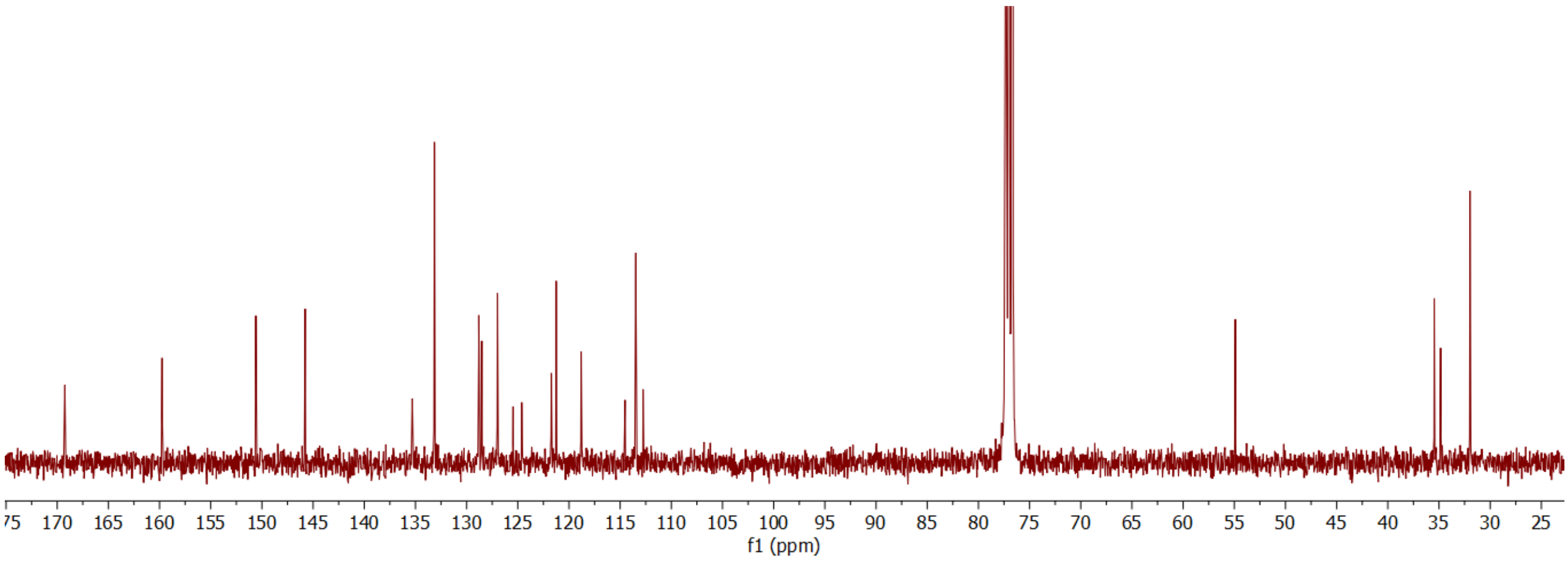
75 170 165 160 155 150 145 140 135 130 125 120 115 110 105 100 95 90 85 80 75 70 65 60 55 50 45 40 35 30 25
f1 (ppm)

ABA trimer (24)

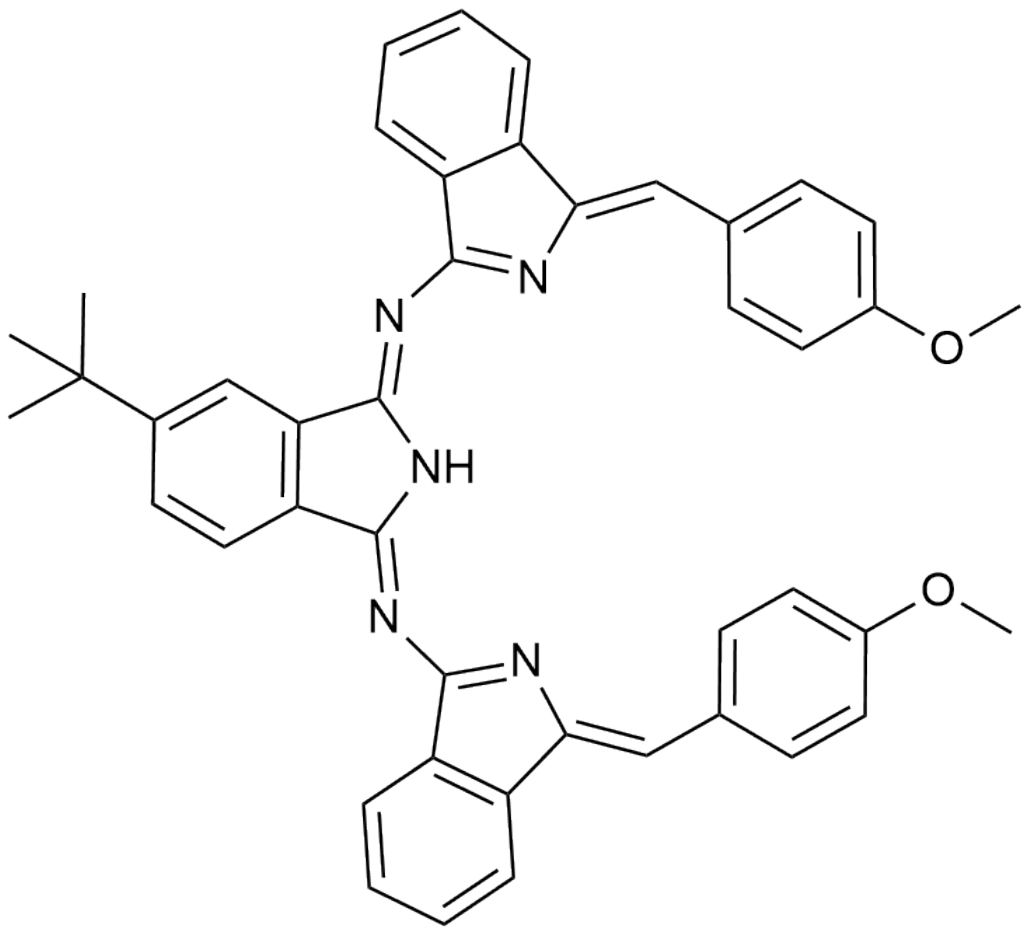

AB condensation product **22a** (100 mg, 0.23 mmol) and aminoisoindoline **1a[7]** (58 mg, 0.23 mmol) were added to a sealed tube and dissolved in 10 mL of *p*-xylene. After purging with nitrogen, the reaction mixture was heated to 180°C in an oil bath overnight. The solvent was removed *in vaccuo* and the crude residue purified by column chromatography (silica gel) eluting with DCM. The brown fractions were collected and recrystallized from DCM/hexane to yield **24** as a brown powder (85 mg, 55 %).

^{1}H NMR (400 MHz, $CDCl_3$) (peaks for major stereoisomer) δ 14.13 (s, 1H), 8.20 (d, J = 1.8 Hz, 1H), 8.09 (d, J = 8.1 Hz, 1H), 8.06 – 8.00 (m, 1H), 8.00 – 7.95 (m, 1H), 7.92 (d, J = 8.4 Hz, 4H), 7.76 (dd, J = 8.0, 1.7 Hz, 1H), 7.73 – 7.67 (m, 2H), 7.52 – 7.40 (m, 4H), 6.61 (s, 2H), 6.39 (d, J = 8.3 Hz, 4H), 3.43 (s, 6H), 1.49 (s, 9H). **^{13}C NMR** (101 MHz, $CDCl_3$) δ 169.39, 159.94, 156.82, 145.94, 145.92, 142.57, 135.47, 133.31, 129.92, 128.95, 128.93, 128.70, 127.18, 126.09, 123.13, 121.37, 121.33, 120.24, 118.95, 113.65, 55.05, 35.86, 31.54. **MP:** 249°C. **MS (MALDI-TOF):** m/z = 667.29 $[M]^{+}$. **MP:** 187-189°C **UV-Vis:** λ_{max} (nm) (ϵ ($dm^3.mol^{-1}.cm^{-1}$)) = 536 ($1.19x10^4$), 343 ($1.78x10^4$), 290 ($4.96x10^4$), 274 ($4.81x10^4$).

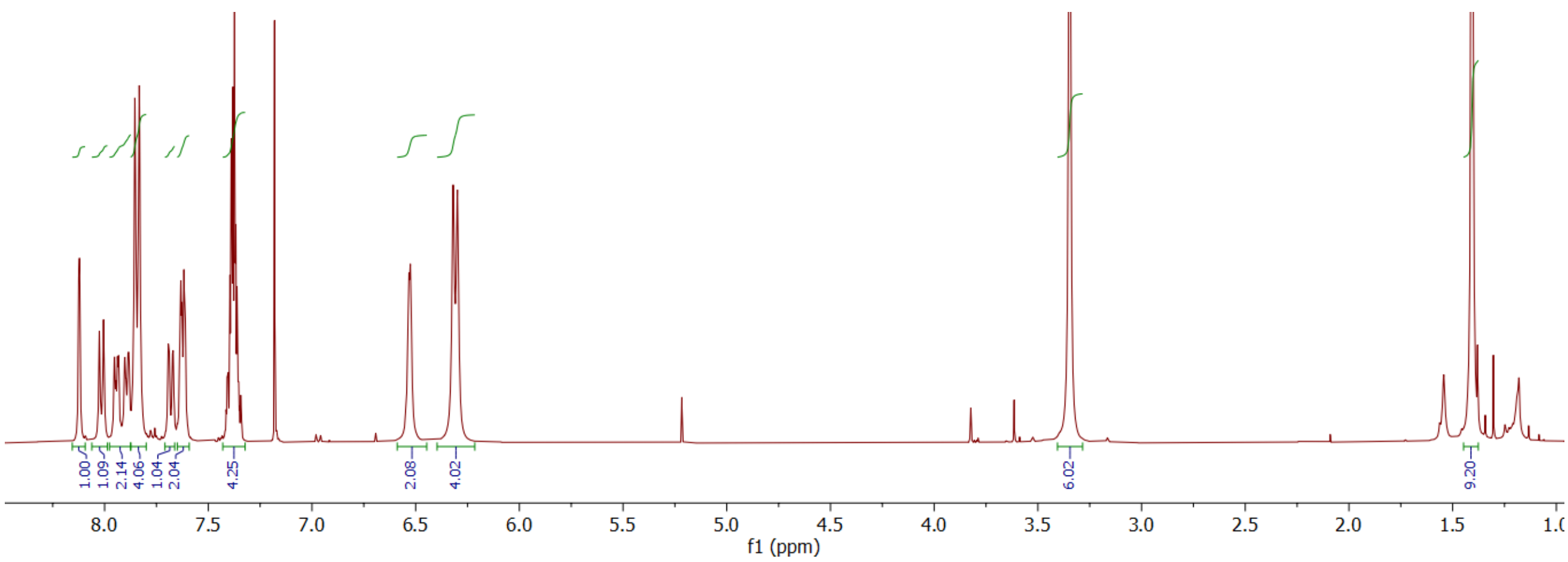

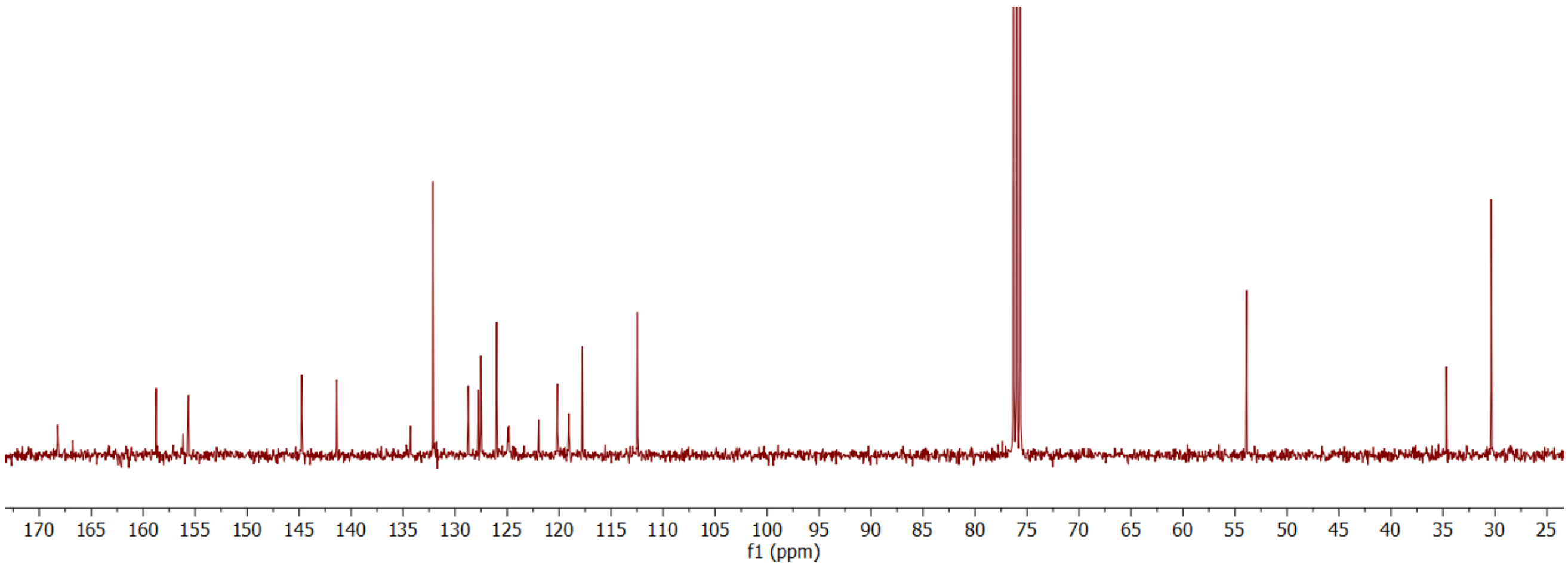

ABA trimer (25)

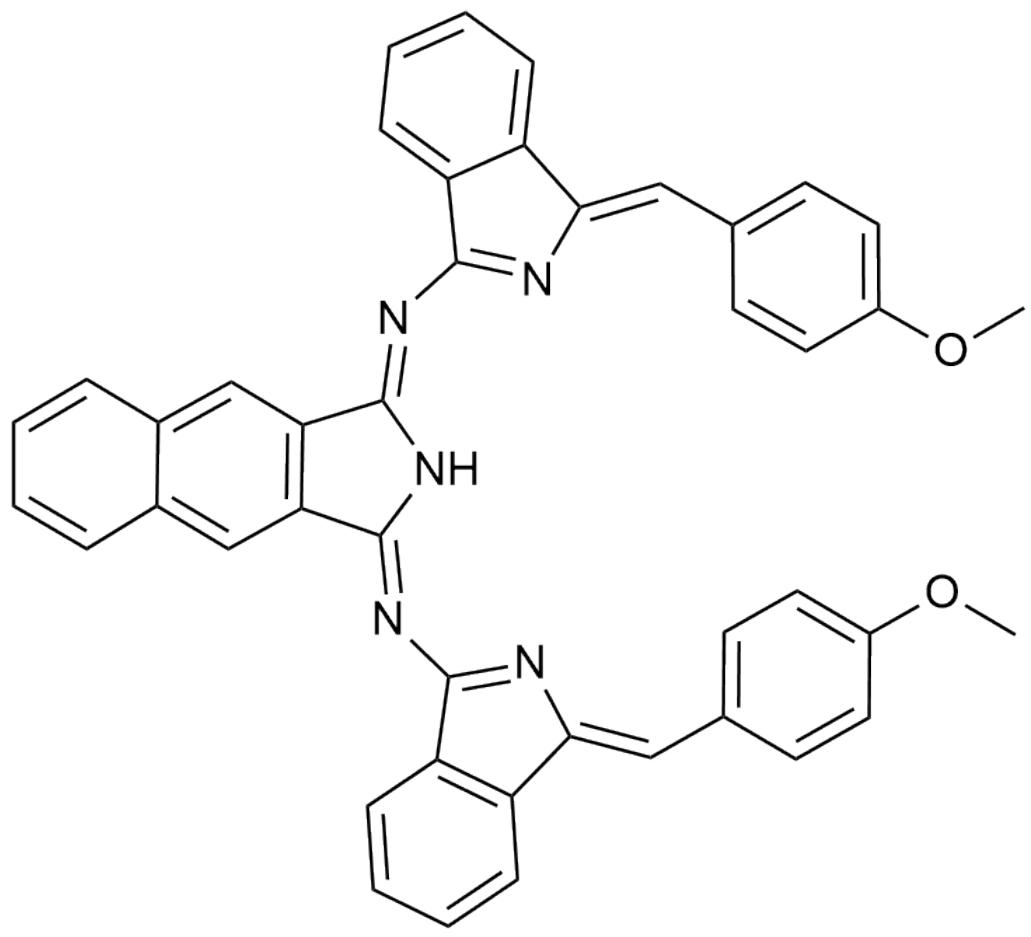

AB condensation product **22b** (100 mg, 0.23 mmol) and aminoisoindoline **1a**[7] (59 mg, 0.24 mmol) were added to a sealed tube and dissolved in 10 mL of *p*-xylene. After purging with nitrogen, the reaction mixture was heated to 180°C in an oil bath overnight. The solvent was removed *in vaccuo* and the crude residue purified by column chromatography (silica gel) eluting with DCM. The brown fractions were collected and recrystallized from DCM/hexane to yield **25** as a brown powder (92 mg, 60 %).

^{1}H NMR (400 MHz, $CDCl_3$) δ 14.44 (s, 1H), 8.72 (s, 2H), 8.14 (dd, J = 6.2, 3.4 Hz, 2H), 8.06 – 8.01 (m, 2H), 7.93 (d, J = 8.4 Hz, 4H), 7.77 – 7.67 (m, 4H), 7.52 – 7.44 (m, 4H), 6.63 (s, 2H), 6.39 (d, J = 8.3 Hz, 4H), 3.42 (s, 6H). **^{13}C NMR** (101 MHz, $CDCl_3$) δ 169.18, 159.81, 156.66, 145.73, 142.45, 135.37, 135.25, 133.19, 131.59, 130.08, 128.80, 128.59, 128.39, 127.07, 125.69, 124.10, 121.20, 118.85, 113.51, 54.91. **MP:** 184-186°C. **MS (MALDI-TOF):** m/z = 661.25 $[M]^+$. **UV-Vis:** λ_{max} (nm) (ϵ ($dm^3.mol^{-1}.cm^{-1}$)) = 536 ($0.46x10^4$), 343 ($0.69x10^4$), 290 ($1.91x10^4$), 274 ($1.86x10^4$).

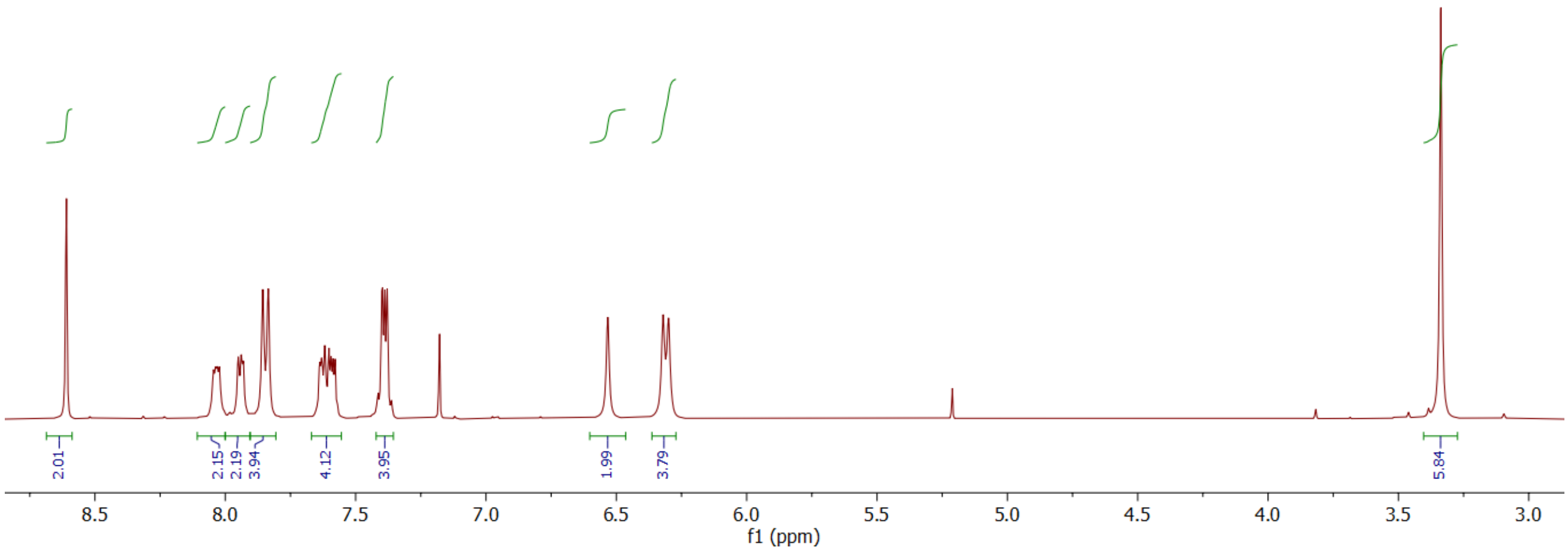

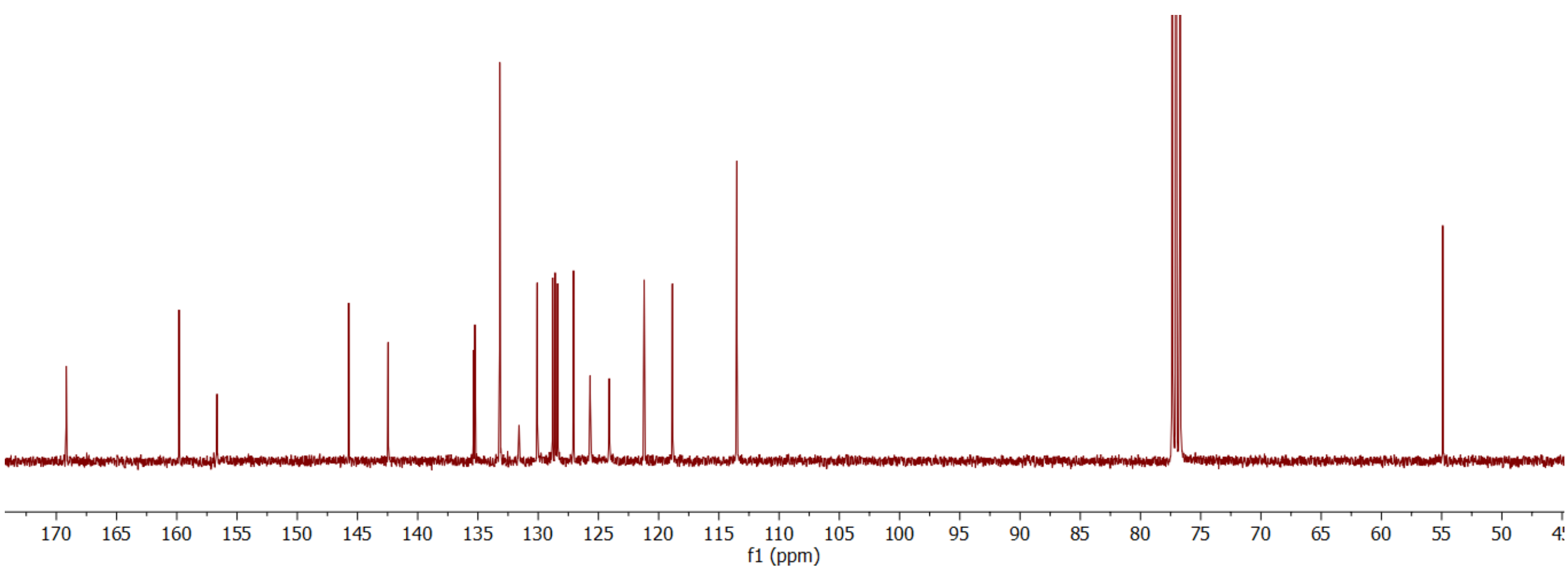

MgTBTAPs from AB dimers

MgTBTAP (2b)[7]

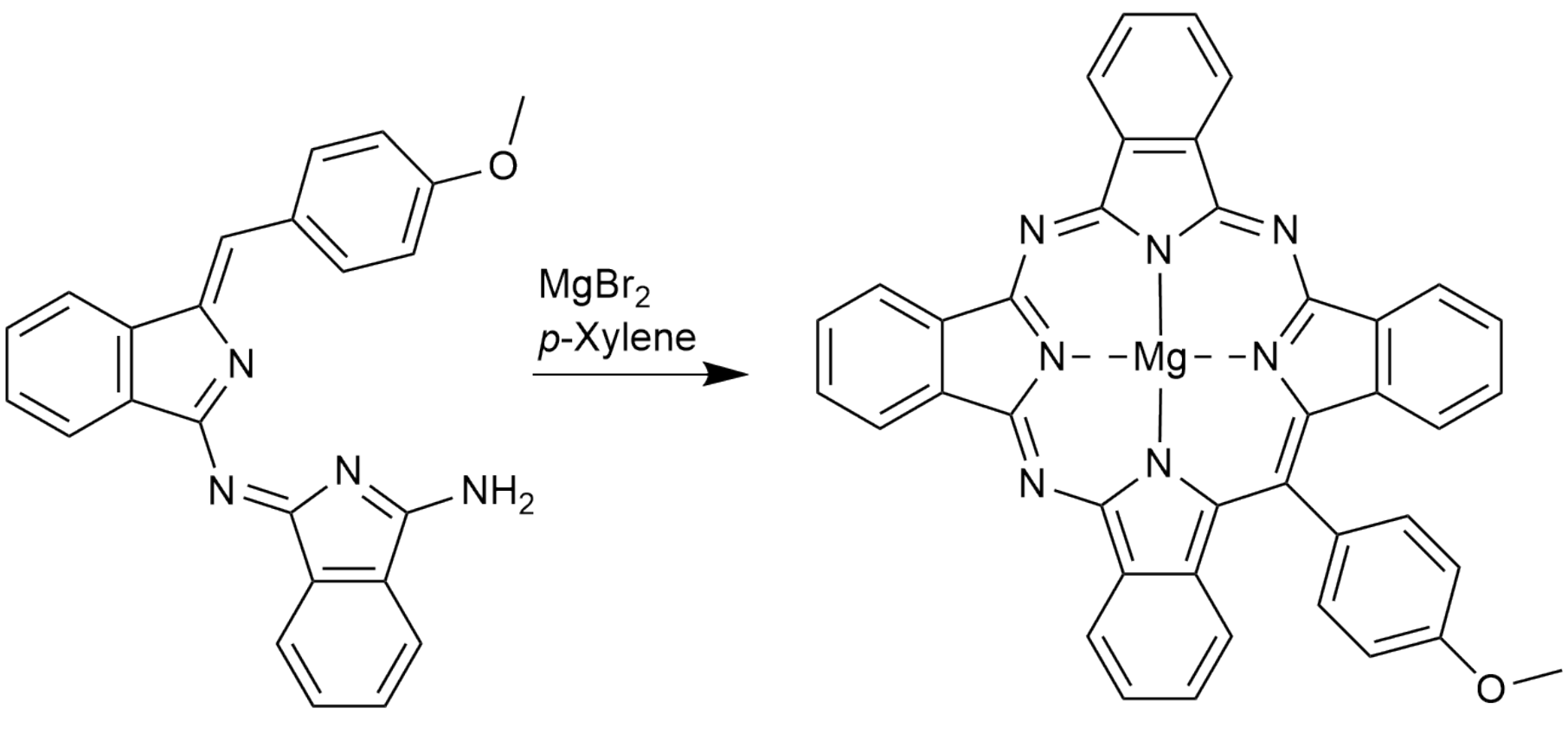

(7a) **(2b)**

Condensation product **7a** (180 mg, 0.48 mmol, 2 eq) and $MgBr_2$ (44 mg, 0.24 mmol, 1 eq) in *p*-xylene (3 mL) were heated to reflux for 3 hrs. After the reaction was complete the solvent was removed *in vaccuo*, and the crude mixture dissolved in DCM/MeOH (50 mL, 1:1) with the aid of sonication. The crude mixture was loaded on silica and purified by silica gel chromatography utilizing DCM/NEt_3/THF (10:1:4). The green fractions were collected and subjected to a second column chromatography eluting with PE/THF/MeOH (10:3:1). The green fractions were then recrystallised from DCM/MeOH to yield **2b** as a dark green solid (62 mg, 40 %).

MgTBTAP (2c)

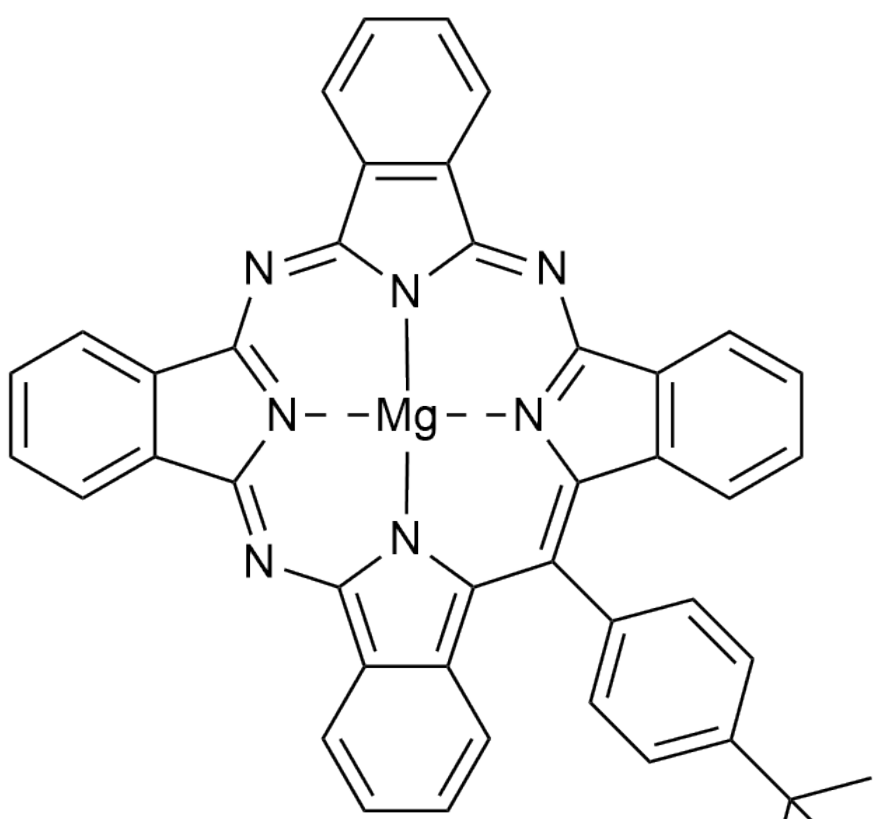

Condensation product **7b** (80 mg, 0.2 mmol, 2 eq) and $MgBr_2$ (18 mg, 0.1 mmol, 1 eq) in p-xylene (3 mL) was heated to reflux for 3 hrs. Following the completion of the reaction, the solvent was removed *in vaccuo.* Then, using sonication, the crude mixture was dissolved in 50 mL of DCM/MeOH (1:1). The crude residue was

purified using silica gel chromatography eluting with DCM and then DCM/THF (10:1). The green fractions were collected, the solvent removed *in vaccuo*, and the residue recrystallized from DCM/MeOH, to obtain **2c** as a dark green solid (30 mg, 45%).

The brown fractions were collected during the workup of **2c** and recrystallised from MeOH/DCM to yield **8c** as brown needles (13 mg, 19 %).

^{1}H NMR (500 MHz, Acetone-d_6) δ 9.60 (d, J = 7.6 Hz, 2H), 9.55 – 9.51 (m, 4H), 8.28 – 8.23 (m, 4H), 8.07 (s, 4H), 7.98 (t, J = 7.2 Hz, 2H), 7.62 (t, 2H), 7.11 (d, J = 8.1 Hz, 2H), 1.76 (s, 9H). **^{13}C NMR** (101 MHz, THF-d_8) δ 155.52, 152.57, 152.41,151.48, 142.41, 140.05, 139.95, 139.82, 139.08,131.68, 128.92, 128.63, 126.83, 125.75, 125.77, 125.60, 124.68, 122.74, 122.52, 122.45, 56.67, 34.66, 31.20. **MP:** 216 - 220°C. **UV-Vis:** λ_{max} (nm) (ϵ ($dm^3.mol^{-1}.cm^{-1}$)) = 678 ($1.64x10^5$), 654 ($1.0x10^5$), 600 ($3.27x10^4$), 450 ($2.83x10^4$), 398 ($6.77x10^4$). **MALDI-TOF:** m/z = 667.42 $[M]^+$ (100%)

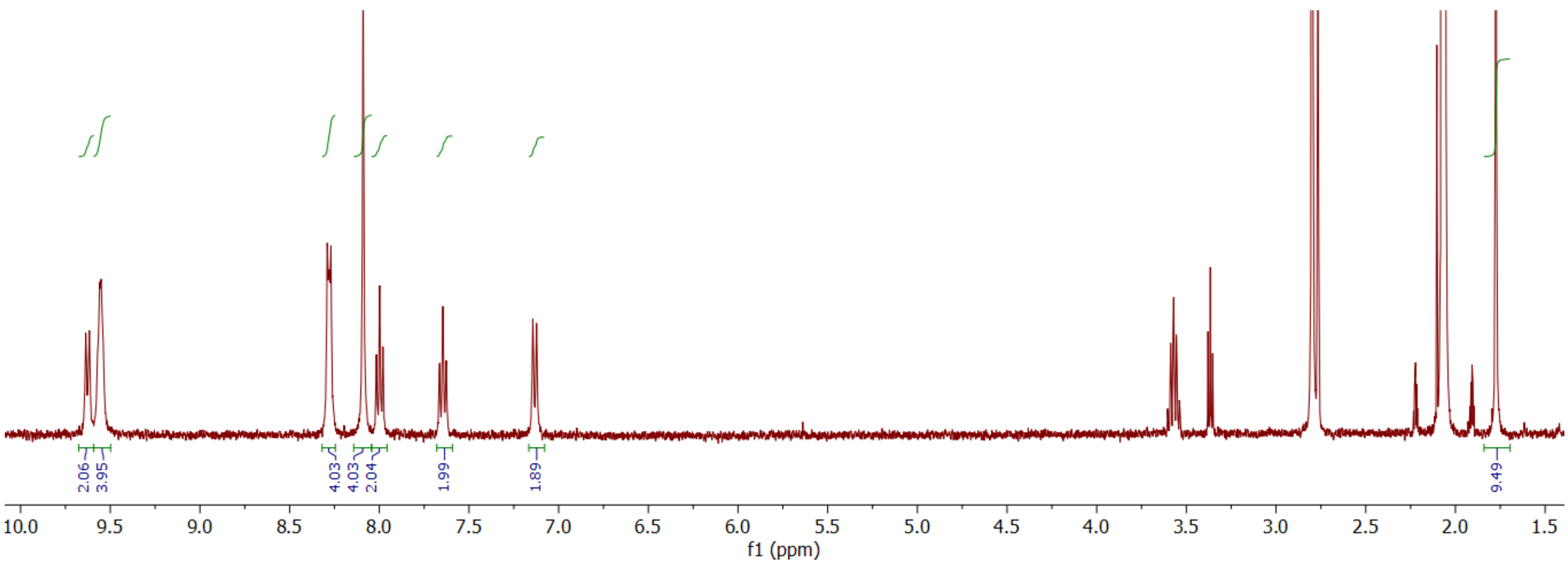

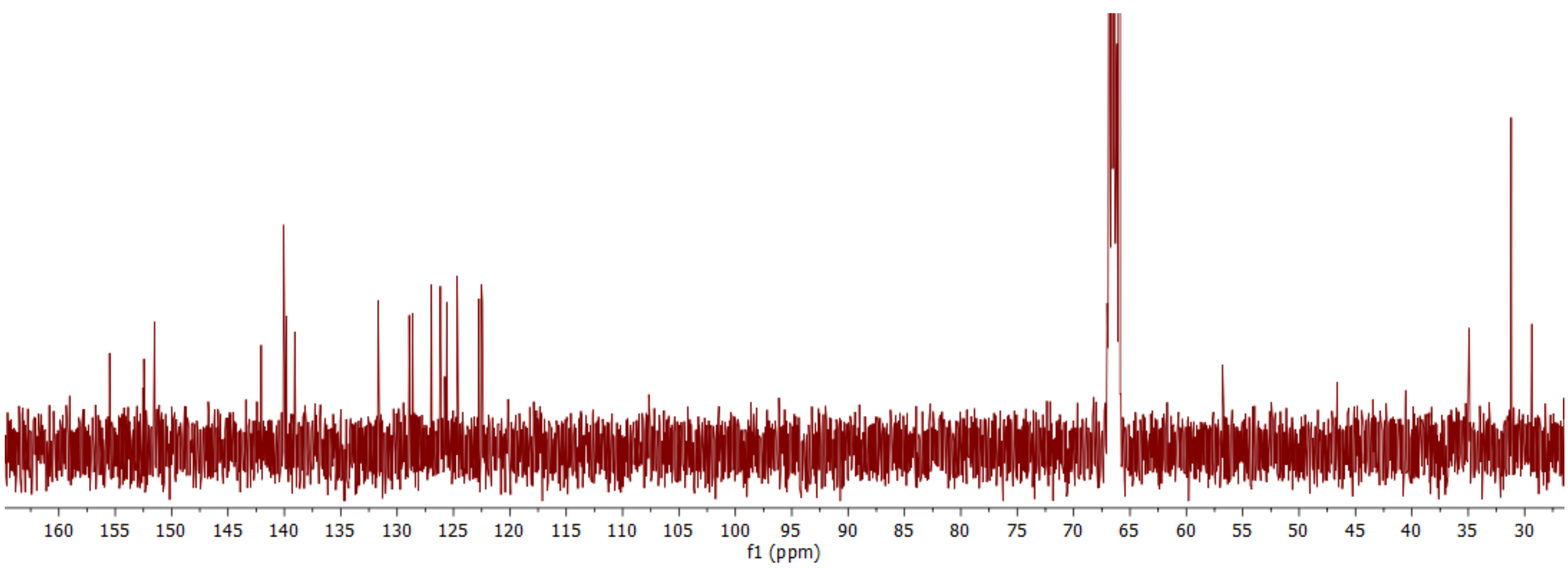

MgTBTAP (2d)[20]

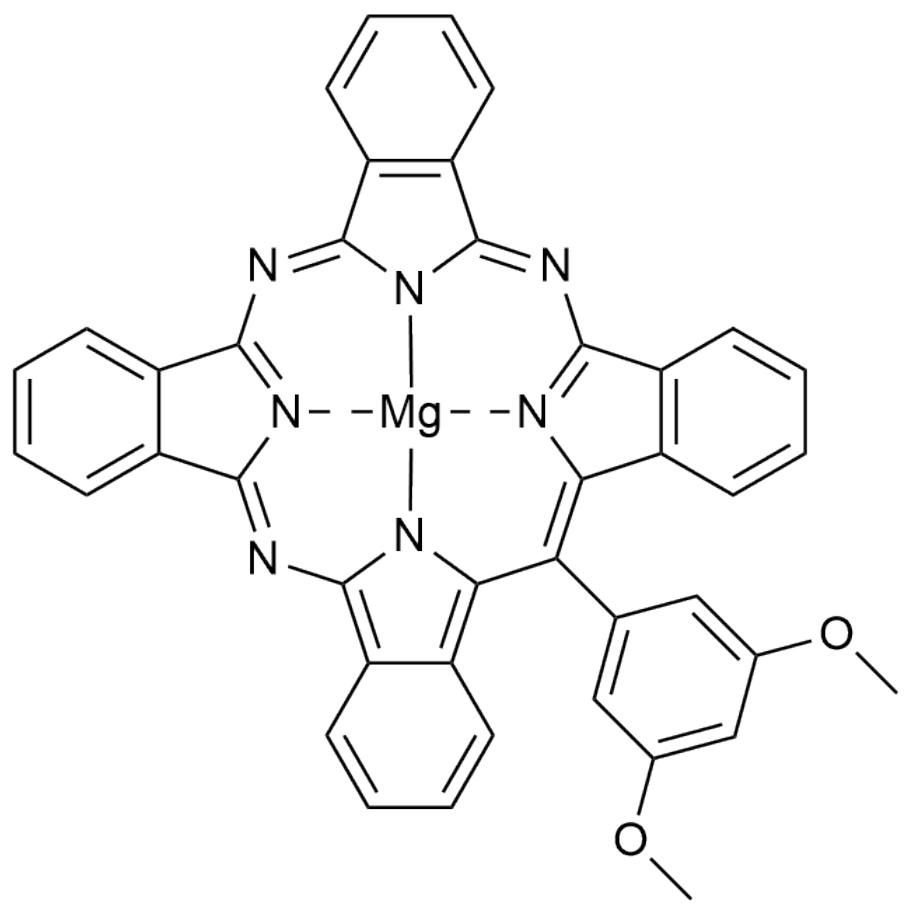

Condensation product **7c** (0.50 g, 1.22 mmol, 1 eq), **6a** (0.47 g, 2.45 mmol, 2 eq), and $MgBr_2$ (0.45 g, 2.45 mmol, 2 eq) in *p*-xylene (20ml) was heated to reflux for 6 hrs. Upon completion the solvent was removed *in vaccuo* and the crude residue dissolved in DCM with the aid of sonication. The crude residue was purified by column chromatography eluting with DCM followed by DCM/THF (10:1). The green fractions were collected and recrystallised from DCM/MeOH to yield **2d** as a dark green solid (80 mg, 20 %).

The brown fractions were collected during the workup of **2d** and recrystallised from MeOH/DCM to yield **8d** as brown needles (20 mg, 4.9 %).

Benzo substituted MgTBTAPs from AB dimers

3:1 MgTBTAP (18)[9]

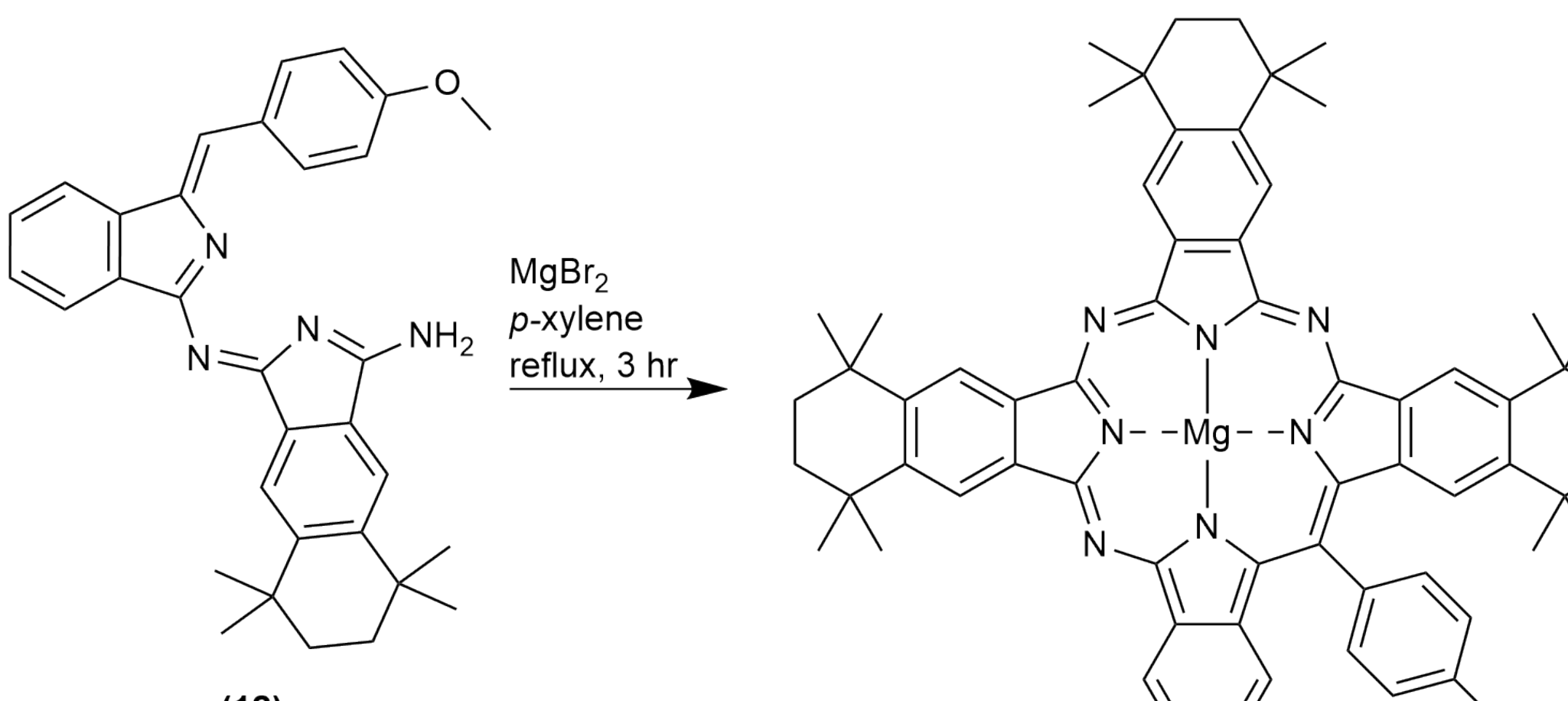

(12) **(18)**

Condensation product **12** (233 mg, 0.48 mmol, 2 eq) was added to $MgBr_2$ (44 mg, 0.24 mmol, 1 eq) in *p*-xylene (3 mL) and the mixture heated to reflux for 3 hr. After the reaction was complete the solvent was removed *in vaccuo*, and the crude mixture dissolved in DCM/MeOH (50 mL, 1:1) with the aid of sonication. The crude material was purified by silica gel chromatography utilizing DCM/NEt_3/THF (10:1:4). The green fractions were collected and subjected to a second column chromatography eluting with PE/THF/MeOH (10:3:1). The green fractions were then recrystallised from DCM/MeOH to yield **18** as a dark green solid (70 mg, 45 %).

3:1 MgTBTAP (19)

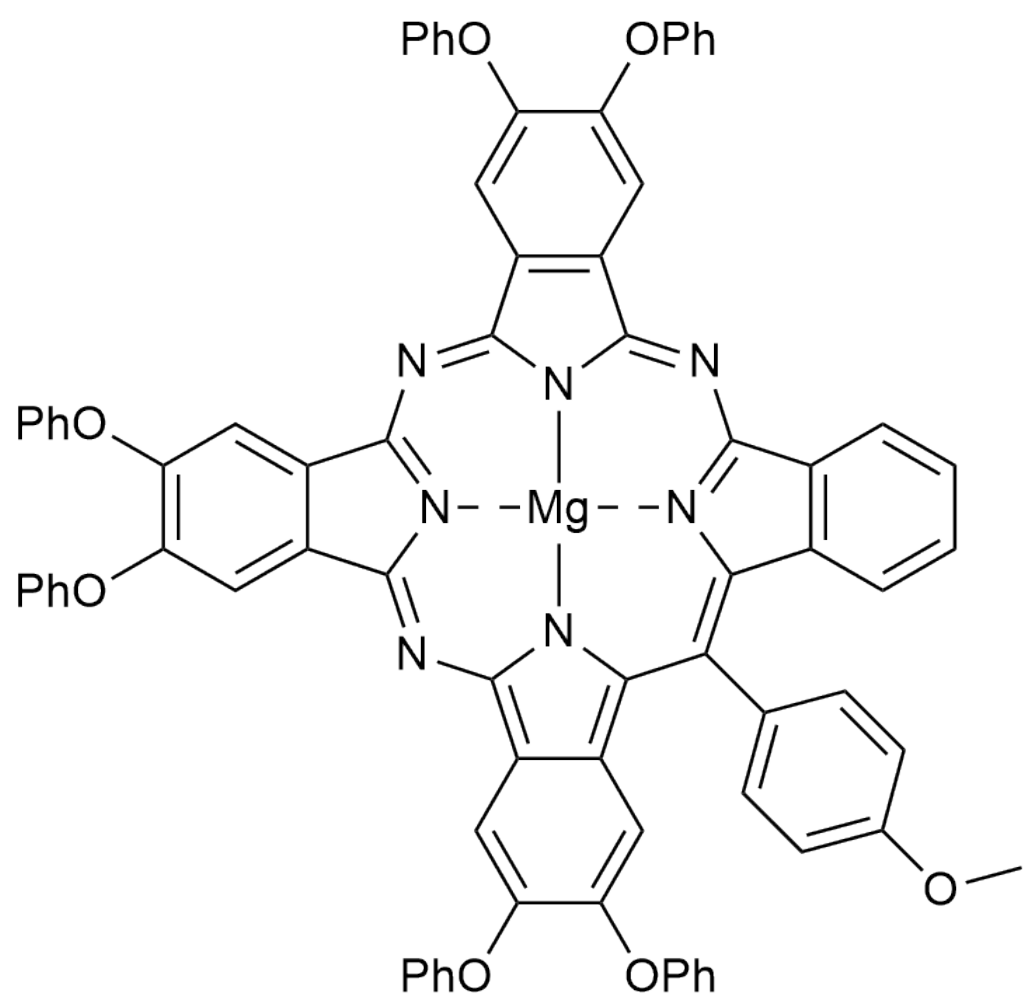

Condensation product **15** (270 mg, 0.48 mmol, 2 eq) was added to a mixture of $MgBr_2$ (44 mg, 0.24 mmol, 1 eq) in *p*-xylene (3 mL) and heated to reflux for 3 hr. After the reaction was complete the solvent was removed *in vaccuo*, and the crude mixture dissolved in DCM/MeOH (50 mL, 1:1) with the aid of sonication. The crude mixture was loaded on silica and purified by silica gel chromatography utilizing DCM/NEt_3/THF (10:1:4). The green fractions were collected and subjected to a second column chromatography eluting with PE/THF/MeOH (10:3:1). The green fractions were then recrystallised from DCM/MeOH to yield **19** as a dark green solid (96 mg, 50 %).

^{1}H NMR (500 MHz, THF-d_8) δ 9.60 (d, J = 7.5 Hz, 1H), 9.56 – 9.47 (m, 5H), 8.08 – 8.01 (m, 2H), 7.90 (t, J = 7.2 Hz, 1H), 7.63 – 7.58 (m, 1H), 7.58 – 7.55 (m, 2H), 7.53 – 7.49 (m, 4H), 7.48 – 7.43 (m, 8H), 7.40 – 7.32 (m, 3H), 7.31 – 7.27 (m, 4H), 7.18 (s, 1H), 7.16 – 7.11 (m, 4H), 7.08 (dd, J = 8.1, 1.2 Hz, 8H), 4.20 (s, 3H). **^{13}C NMR** (125.7 MHz, THF-d_8) δ 160.60, 158.58, 158.19, 158.13, 158.05, 157.95, 157.93, 157.11, 150.20, 150.07, 149.92, 149.50, 149.41, 146.89, 132.53, 129.68, 129.66, 129.61, 129.57, 129.43, 129.38, 123.37, 123.18, 123.15, 123.09, 123.03, 122.93, 122.90, 122.44, 118.79, 118.11, 118.04, 117.96, 117.85, 117.77, 117.72, 117.67, 117.16, 114.09, 54.72. **MP:** > 300°C. **UV-Vis:** λ_{max} (nm) (ϵ ($dm^3.mol^{-1}.cm^{-1}$)) = 682 (9.81x10^4), 655 (5.04x10^4), 443 (1.92x10^4), 389 (4.30x10^4). **MALDI-TOF:** m/z = 1193.42 $[M]^+$ (100%).

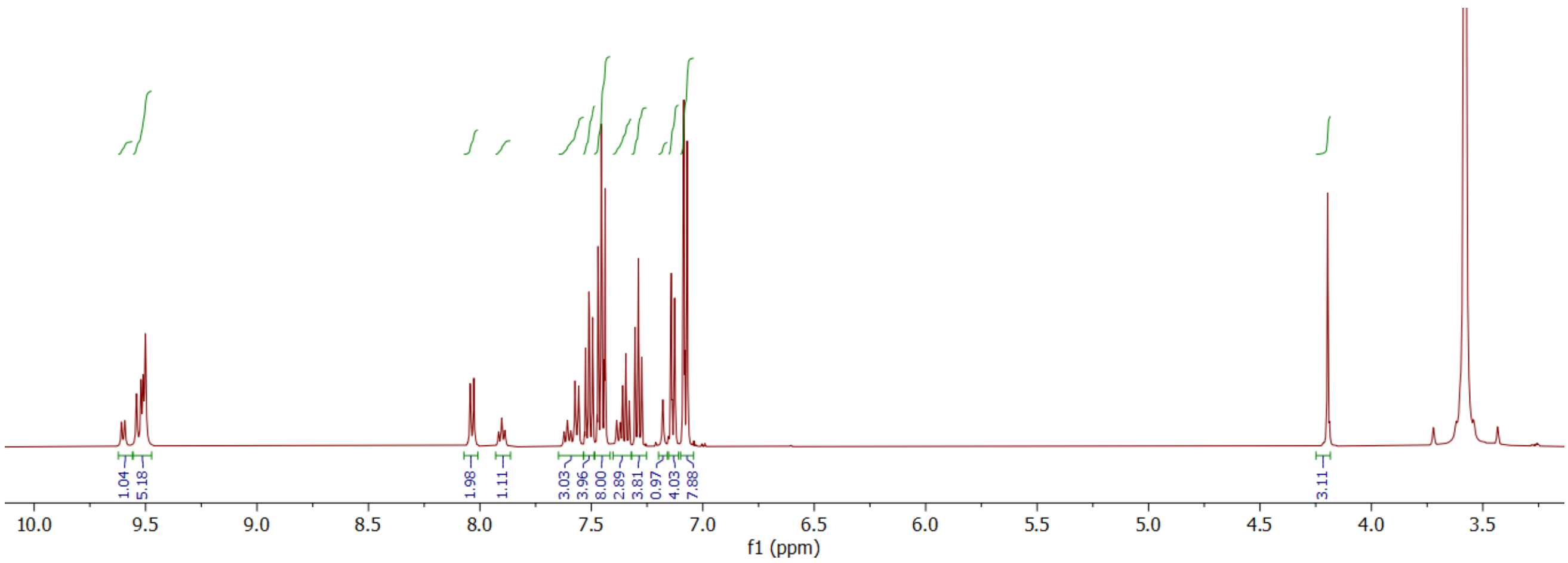
1.04
5.18
1.98
1.11
3.03
3.96
8.00
2.89
3.81
0.97
4.03
7.88
3.11
10.0 9.5 9.0 8.5 8.0 7.5 7.0 6.5 6.0 5.5 5.0 4.5 4.0 3.5
f1 (ppm)

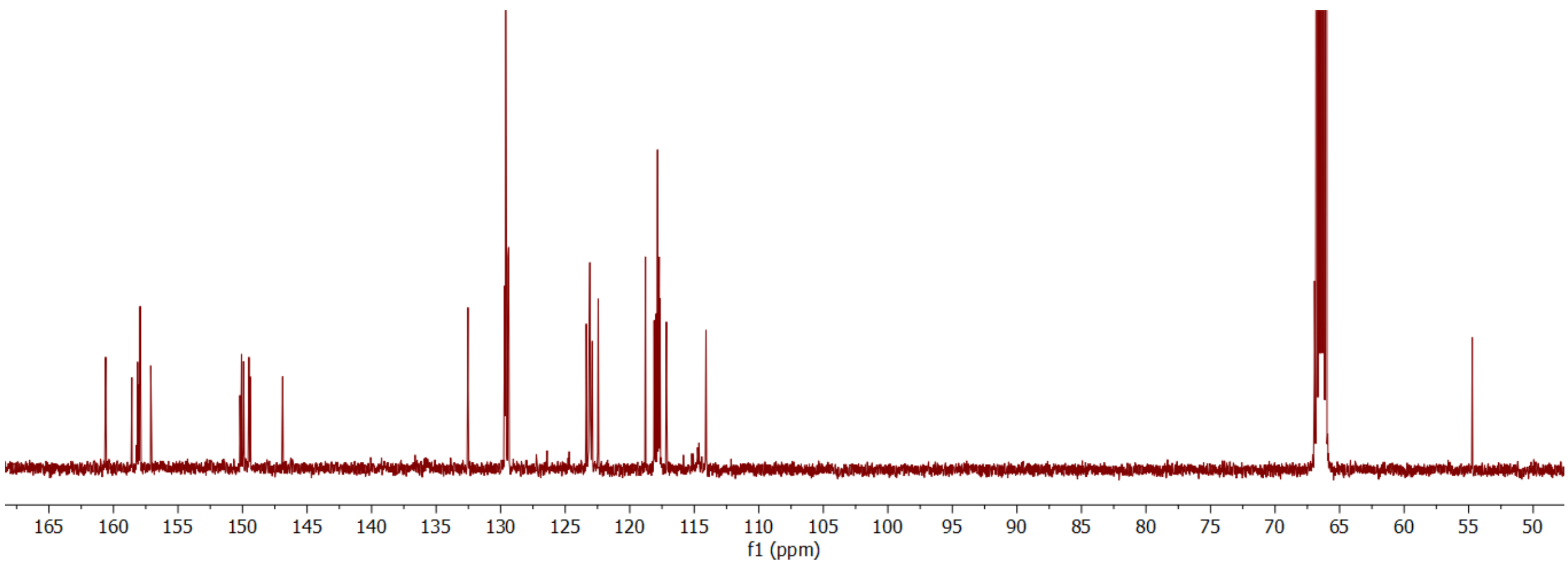
165 160 155 150 145 140 135 130 125 120 115 110 105 100 95 90 85 80 75 70 65 60 55 50
f1 (ppm)

1:3 MgTBTAP (20)

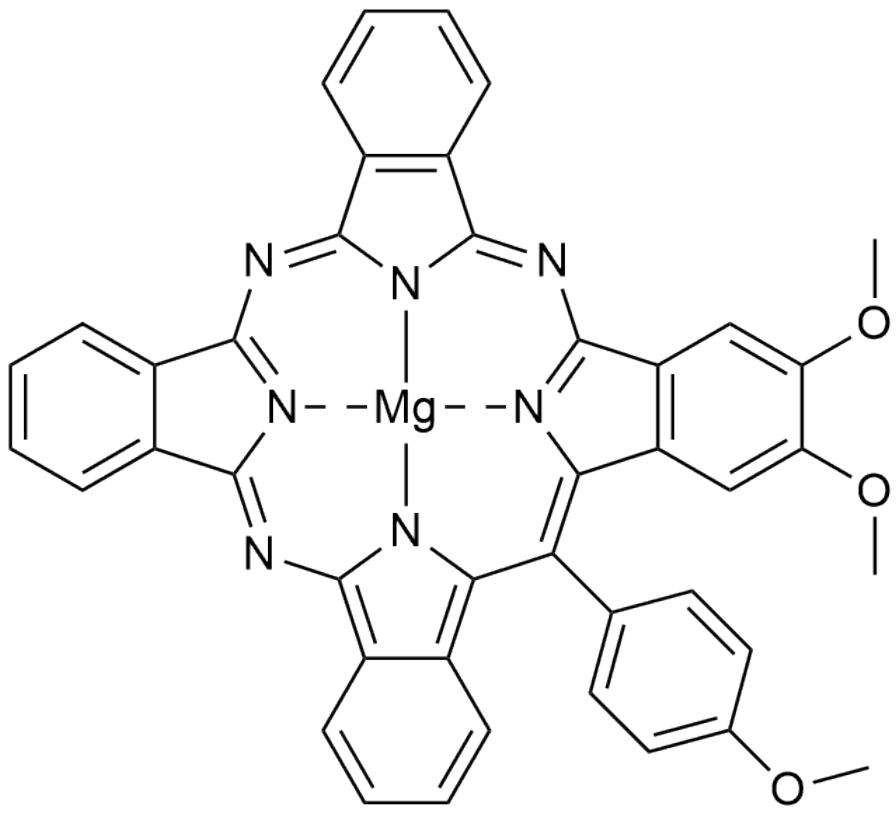

Condensation product **16** (209 mg, 0.48 mmol, 2 eq) was added to a mixture of $MgBr_2$ (44 mg, 0.24 mmol, 1 eq) in *p*-xylene (3 mL) and heated to reflux for 3 hr. After the reaction was complete the solvent was removed *in vaccuo*, and the crude mixture dissolved in DCM/MeOH (50 mL, 1:1) with the aid of sonication. The crude mixture was loaded on silica and purified by silica gel chromatography utilizing DCM/NEt_3/THF (10:1:4). The green fractions were collected and subjected to a second column chromatography eluting with PE/THF/MeOH (10:3:1). The green fractions were then recrystallised from DCM/MeOH to yield **20** as a dark green solid (58 mg, 52 %).

^{1}H NMR (500 MHz, THF-d_8) δ 9.61 (d, J = 7.6 Hz, 1H), 9.55 – 9.48 (m, 4H), 9.03 (s, 1H), 8.21 – 8.15 (m, 4H), 8.07 (d, J = 8.4 Hz, 2H), 7.92 (t, J = 7.3 Hz, 1H), 7.62 (t, J = 7.3 Hz, 1H), 7.57 (d, J = 8.4 Hz, 2H), 7.45 (d, J = 8.0 Hz, 1H), 6.64 (s, 1H), 4.30 (s, 3H), 4.17 (s, 3H), 3.78 (s, 3H). **^{13}C NMR** (125.7 MHz, $CDCl_3$) δ 160.85, 155.89, 153.36, 150.30, 142.91, 139.40, 136.90, 135.56, 133.07, 128.82, 128.71, 128.64, 128.47, 126.92, 126.36, 126.18, 124.70, 122.69, 122.67, 122.64, 122.48, 116.84, 114.39, 106.78, 103.84, 55.50, 55.07, 54.89. **MP:** > 300°C. **UV-Vis:** λ $_{max}$ (nm) (ϵ (dm^3.mol^{-1}.cm^{-1})) = 674 (8.41x10^4), 648 (3.92x10^4), 442 (1.05x10^4), 401 (2.79x10^4). **MALDI-TOF:** m/z = 700.38 [M]$^+$ (100%).

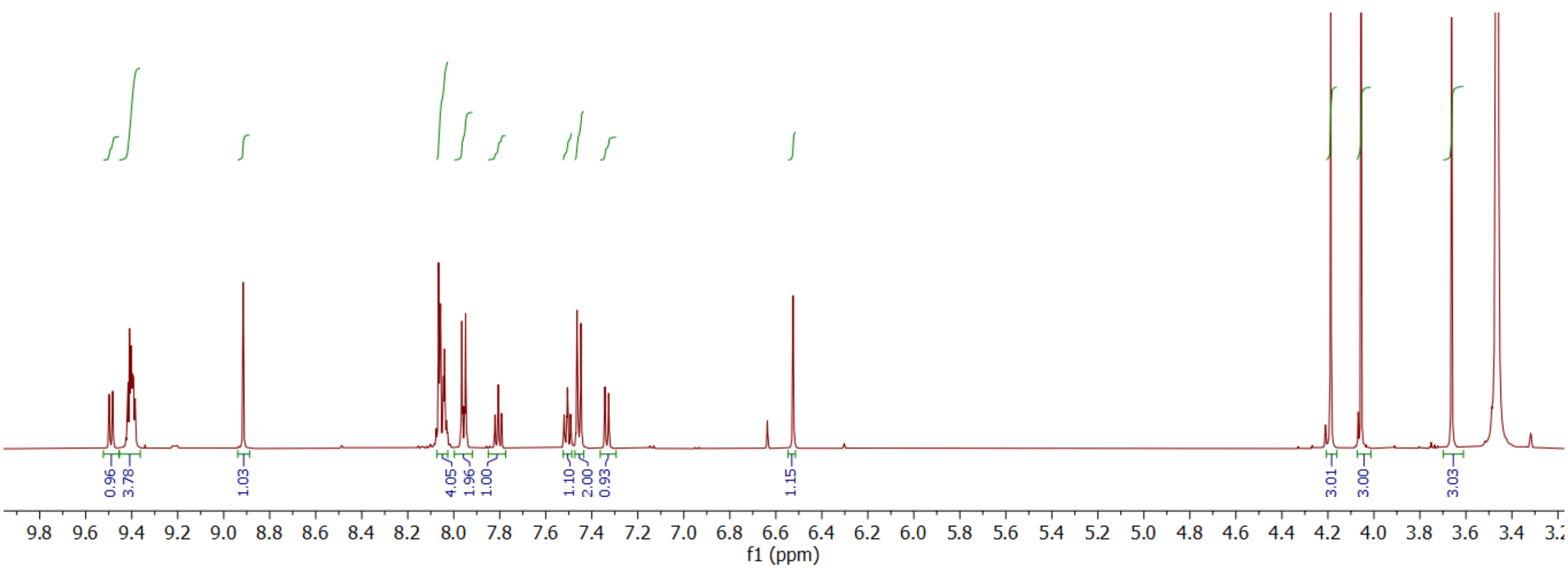

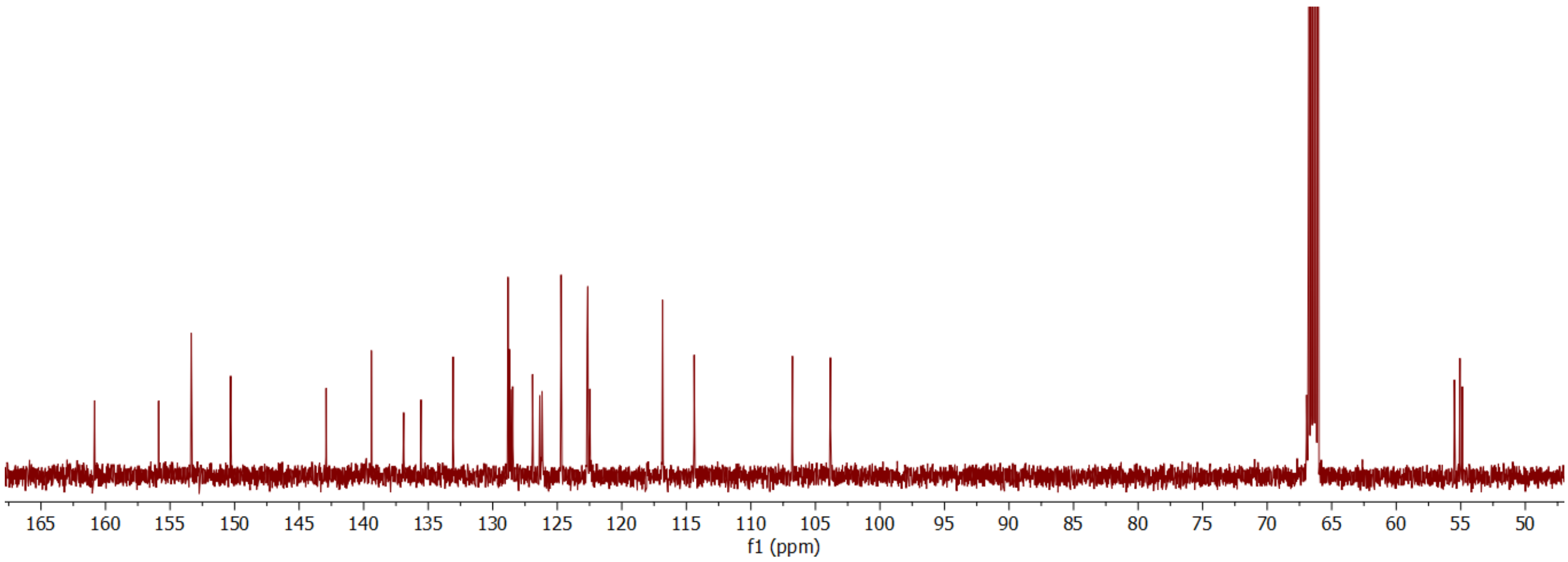
165 160 155 150 145 140 135 130 125 120 115 110 105 100 95 90 85 80 75 70 65 60 55 50
f1 (ppm)

Pathway analysis for reactions (scrambling) of AB dimers

DFT calculations were performed at the B3LYP/6-31++G(d,p) level of theory using the Gaussian16 software package. Atomic charges for AB dimer **1a** were calculated via natural bond order analysis (NBO). Approximately equal partial positive charge (electrophilicity) of the three reaction sites was identified as shown by the colour map.

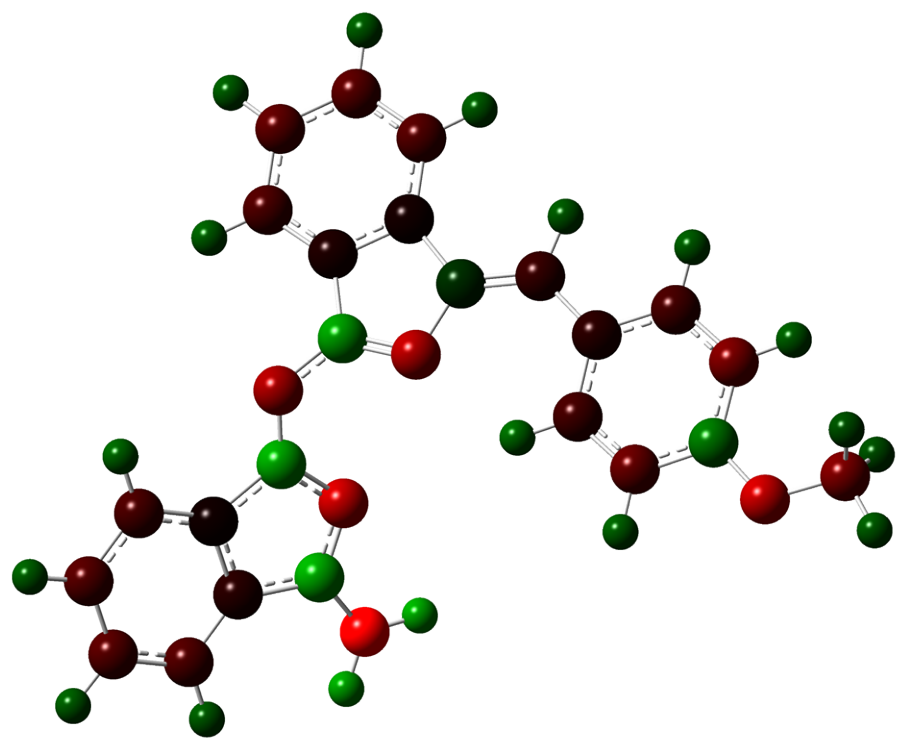

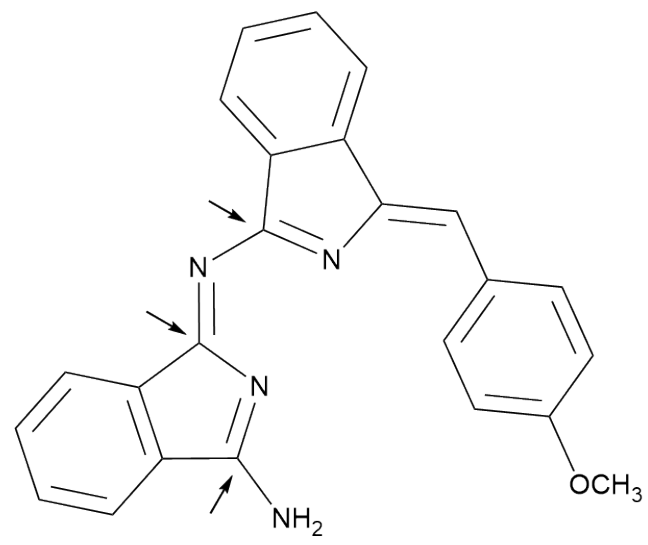

MALDI-TOF analysis of low temperature reaction conditions showing scrambling of the AB starting material and formation of key intermediates.

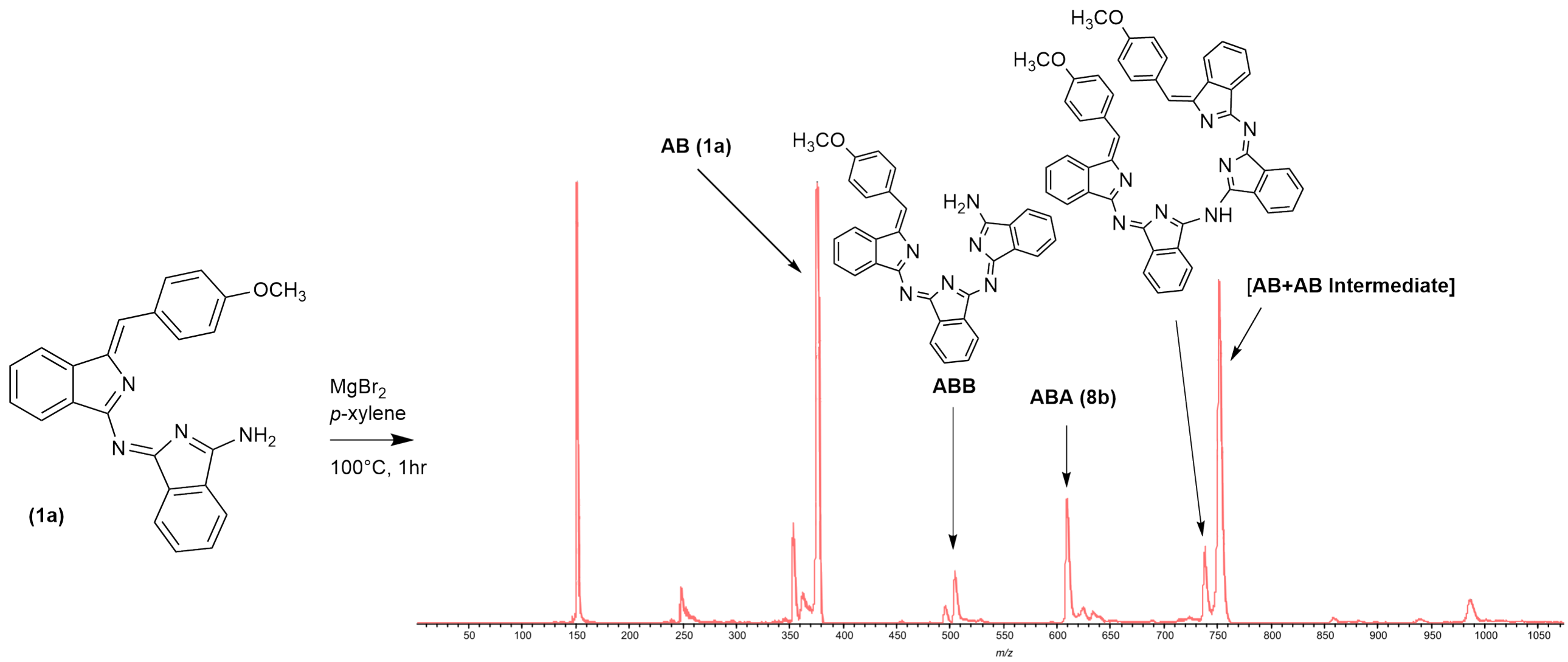

X-ray crystal structures

Crystal data and structure refinement for ABA trimer **25**

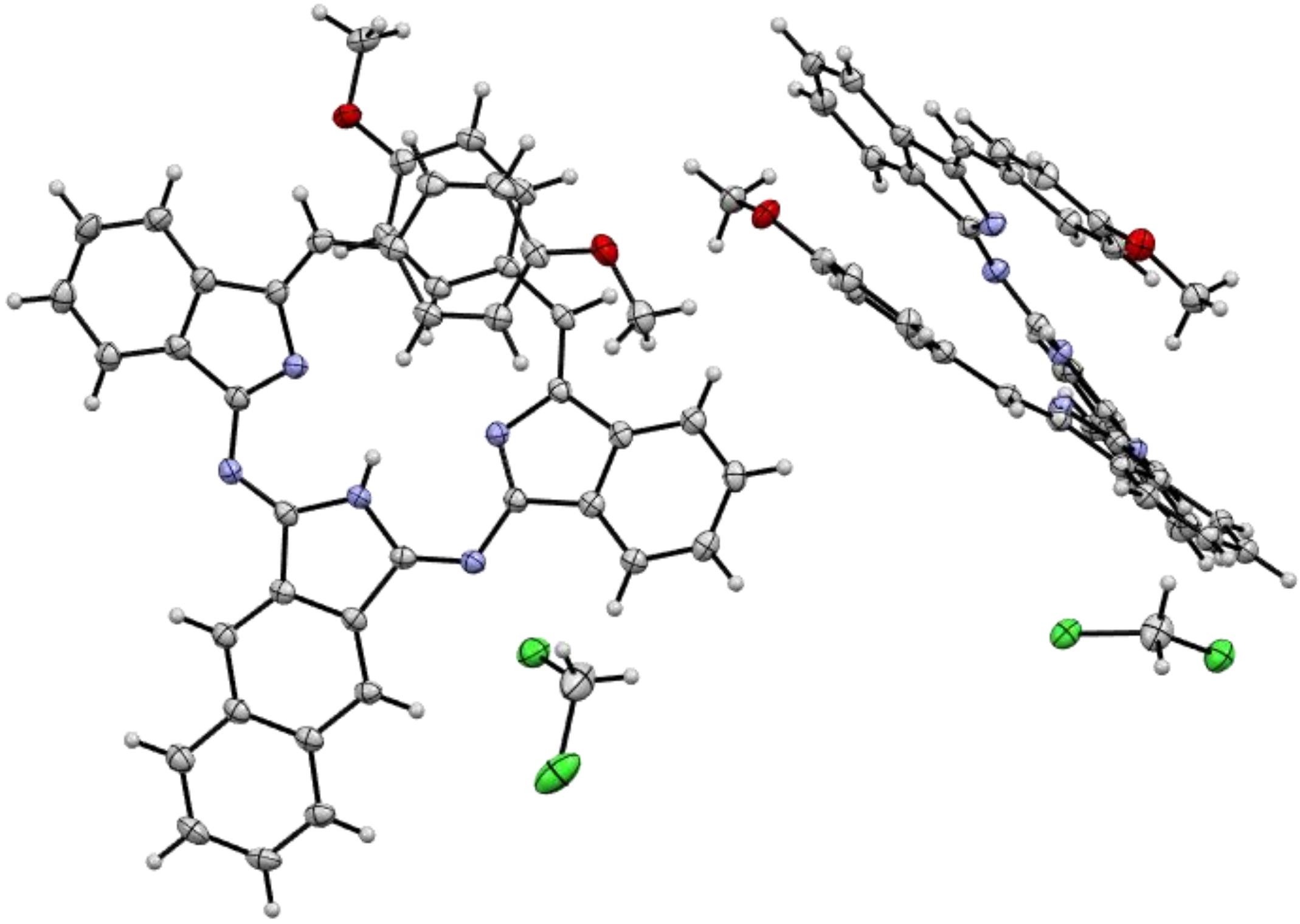

Identification code	**25**
Elemental formula	2(C44 H30 N5 O2, C H2 Cl2)
Formula weight	1493.32
Crystal system, space group	Orthorhombic, $Pca2_1$ (no. 29)
Unit cell dimensions	a = 16.1812(3) Å α = 90 °
	b = 14.6930(3) Å β = 90 °
	c = 30.2522(6) Å γ = 90 °
Volume	7192.5(2) $Å^3$
Z, Calculated density	4, 1.379 Mg/m^3
F(000)	3104
Absorption coefficient	2.004 mm^{-1}
Temperature	106(2) K
Wavelength	1.54184 Å
Crystal colour, shape	dark orange block
Crystal size	0.72 x 0.45 x 0.43 mm
Crystal mounting:	on a small loop, in oil, fixed in cold N_2 stream

On the diffractometer:

Theta range for data collection	2.921 to 72.479 °
Limiting indices	-19<=h<=15, -17<=k<=17, -36<=l<=36
Completeness to theta = 67.684	99.7 %
Absorption correction	Semi-empirical from equivalents
Max. and min. transmission	1.00000 and 0.24577
Reflections collected (not including absences)	29676
No. of unique reflections	11425 [R(int) for equivalents = 0.041]
No. of 'observed' reflections ($I > 2\sigma I$)	10830
Structure determined by:	dual methods, in SHELXT
Refinement:	Full-matrix-block least-squares on F^2, in SHELXL
Data / restraints / parameters	11425 / 1 / 985
Goodness-of-fit on F^2	1.055
Final R indices ('observed' data)	R_1 = 0.039, wR_2 = 0.102
Final R indices (all data)	R_1 = 0.041, wR_2 = 0.103

Reflections weighted:
$w = [\sigma^2(Fo^2)+(0.0652P)^2+0.8113P]^{-1}$ where $P=(Fo^2+2Fc^2)/3$

Absolute structure parameter	0.104(8)
Extinction coefficient	n/a
Largest diff. peak and hole	0.48 and -0.43 e.Å^{-3}
Location of largest difference peak	near Cl(95)

Crystal structure analysis of 25

Crystal data: 2($C_{44}H_{30}N_5O_2$, CH_2Cl_2), M = 1493.32. Orthorhombic, space group $Pca2_1$ (no. 29), a = 16.1812(3), b = 14.6930(3), c = 30.2522(6) Å, α = 90, β = 90, γ = 90 °, V = 7192.5(2) $Å^3$. Z = 4, Dc = 1.379 g cm^{-3}, F(000) = 3104, T = 106(2) K, μ(Cu-Kα) = 20.0 cm^{-1}, λ(Cu-Kα) = 1.54184 Å.

The crystal was a dark orange block. From a sample under oil, one, *ca* 0.43 x 0.45 x 0.72 mm, was mounted on a small loop and fixed in the cold nitrogen stream on a Rigaku Oxford Diffraction XtaLAB Synergy diffractometer, equipped with Cu-Kα radiation, HyPix detector and mirror monochromator. Intensity data were measured by thin-slice ω-scans. Total no. of reflections recorded, to θ_{max} = 72.5°, was 29,676 of which 11,425 were unique (Rint = 0.041); 10,830 were 'observed' with $I > 2\sigma_I$.

Data were processed using the CrysAlisPro-CCD and -RED (CrysAlisPro, Rigaku Oxford Diffraction Ltd., Abingdon, UK (2018)) programs. The structure was determined by the intrinsic phasing routines in the SHELXT program (G. M. Sheldrick, Programs for crystal structure determination (SHELXT), *Acta Cryst.* (2015) A**71**, 3-8) and refined by full-matrix least-squares methods, on F^2's, in SHELXL (*Acta Cryst.* (2008) A**64**, 112-122 and (2015) C**71**, 3-8). There are two organic molecules and two solvent, CH_2Cl_2 molecules in the asymmetric unit. The non-hydrogen atoms were refined with anisotropic thermal parameters. The hydrogen atoms on N(1) and N(101 were located in a difference map and were refined freely. The remaining hydrogen atoms were included in idealised positions and their Uiso values were set to ride on the Ueq values of the parent carbon atoms. At the conclusion of the refinement, wR_2 = 0.103 and R_1 = 0.041 (2B) for all 11,425 reflections weighted $w = [\sigma^2(F_o^2) + (0.0625\ P)^2 + 0.8113\ P]^{-1}$ with $P = (F_o^2 + 2F_c^2)/3$; for the 'observed' data only, R_1 = 0.039. The Flack x parameter is 0.104(8) and the diagrams show the correct absolute configuration.

In the final difference map, the highest peak (*ca* 0.5 e$Å^{-3}$) was near Cl(95).

Scattering factors for neutral atoms were taken from (*'International Tables for X-ray Crystallography'*, Kluwer Academic Publishers, Dordrecht (1992). Vol. C, pp. 500, 219 and 193). Computer programs used in this analysis have been noted above, and were run through WinGX (L. J. Farrugia, *J. Appl. Cryst.* (2012) **45**, 849–854) on a Dell Optiplex 780 PC at the University of East Anglia.

Notes on the structure

The crystal structure analysis shows two independent, tris-isoindole molecules and two solvent (CH_2Cl_2) molecules in the asymmetric unit. The principal molecules have very similar dimensions and conformations; the only significant differences are in the orientations of one of the methoxy groups; the methoxy group of O(57)–C(58) is the odd one out, and the crystals are therefore racemic mixtures.

Each molecule forms a spiral chain, from O(31) to C(24), N(14), C(40), C(50) and O(57) in the first molecule and correspondingly from O(131) to O(157) in the second. The phenyl groups in each molecule are essentially parallel and overlapping with interplanar distances *ca* 3.4 Å in both molecules.

The central, benzo-isoindole nitrogen atom in each molecule, N(1) and N(101), has a hydrogen attached; these hydrogen atoms form bifurcated hydrogen bonds to the other isoindole N atoms in the molecule. Each solvent molecule donates one hydrogen to a hydrogen bond to a 'bridging' N atom (between isoindole groups). There are several instances of $\pi \ldots \pi$ contacts between parallel (or almost parallel) planes but stacking appears to be limited to three planes.

Crystal data and structure refinement details for **8a**.

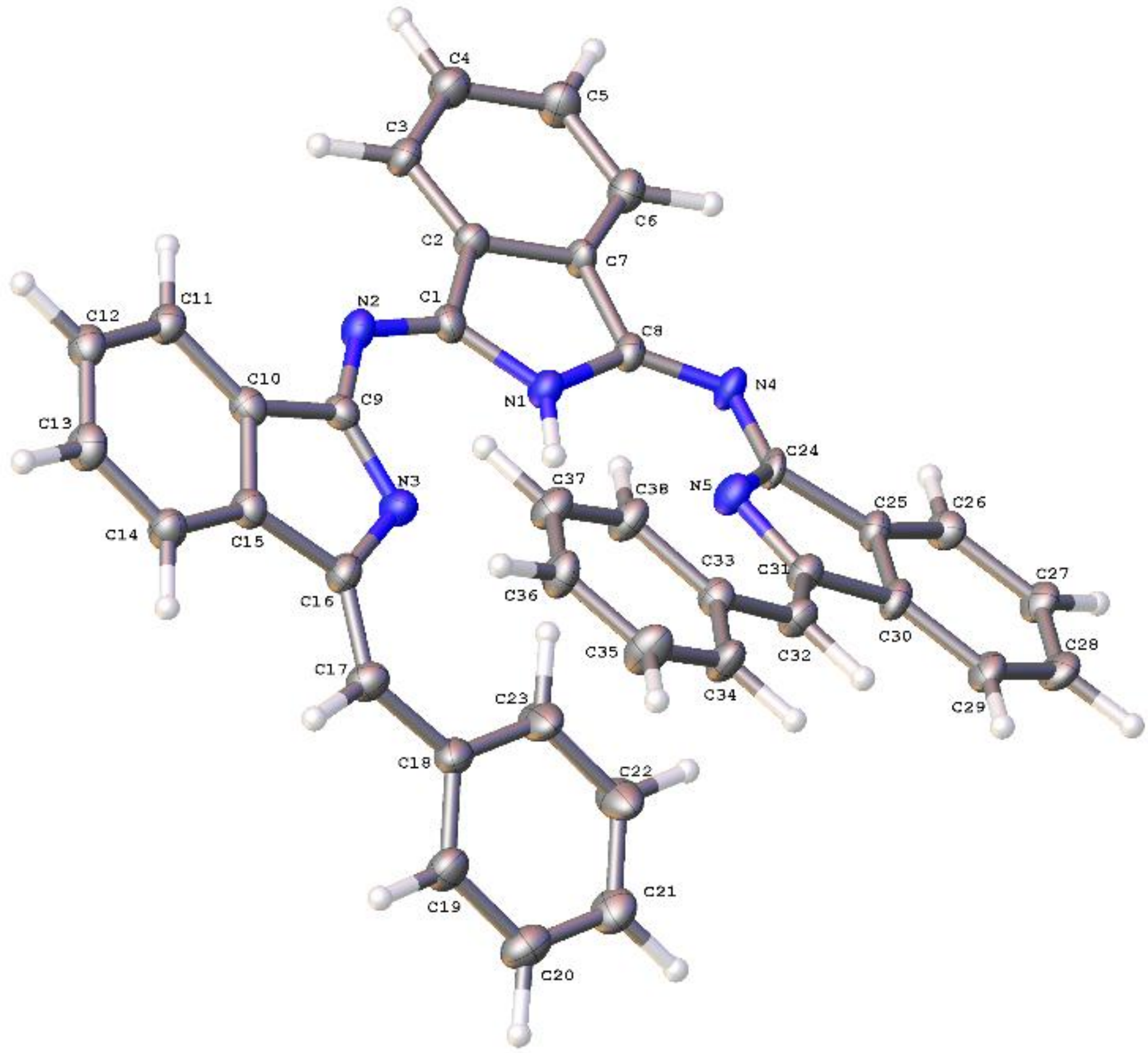

**Compound**	**8a**
Formula	$C_{38}H_{25}N_5$
$D_{calc.}$/ g cm^{-3}	1.333
μ/mm^{-1}	0.080
Formula Weight	551.656
Colour	red
Shape	block-shaped
Size/mm	0.03×0.03×0.01
T/K	100(2)
Crystal System	triclinic
Space Group	P-1
a/Å	11.5165(10)
b/Å	12.2746(10)
c/Å	12.3845(11)
α/°	115.823(8)
β/°	106.203(8)
γ/°	103.866(7)
V/Å^3	1374.8(3)
Z	2
Z'	1
Wavelength/Å	0.71073
Radiation type	Mo K_α
Θ_{min}/°	2.78
Θ_{max}/°	25.03
Index range h	-13 ≥ h ≥ 13
Index range k	-13 ≥ k ≥ 14
Index range l	-14 ≥ l ≥ 13
Measured Refl's.	14910
Indep't Refl's	4850
Refl's I≥2σ(I)	2510
R_{int}	0.1056
Parameters	388
Restraints	0
Largest Peak/eÅ^3	0.6345
Deepest Hole/eÅ^3	-0.8184
GooF	1.0176
R_1 (I≥2σ(I) / all)	0.0679 / 0.1594
wR_2 (I≥2σ(I) / all)	0.1014 / 0.1250

A red block-shaped crystal with dimensions 0.03 × 0.03 × 0.01 mm was mounted on a MITIGEN holder in oil. Data were collected using a Rigaku FRE+ diffractometer equipped with VHF Varimax confocal mirrors and an AFC12 goniometer and HG Saturn 724+ detector diffractometer equipped with an Oxford Cryosystems low-temperature device operating at T = 100(2) K.

Data were measured using profile data from ω-scans with Mo K_α radiation. The diffraction pattern was indexed and the total number of runs and images was based on the strategy calculation from the program CrystalClear-SM Expert 3.1 b27 (Rigaku, 2013) . The maximum resolution achieved was Θ = 25.03° (0.84 Å).

The unit cell was refined using CrysAlis^{Pro} on 4427 reflections, 30% of the observed reflections.

Data reduction, scaling and absorption corrections were performed using CrysAlis^{Pro}. The final completeness is 99.84 % out to 25.03° in Θ. A multi-scan absorption correction was performed using

CrysAlis[Pro]. The absorption coefficient μ of this material is 0.080 mm^{-1} at this wavelength (λ = 0.71073Å) and the minimum and maximum transmissions are 0.849 and 1.000.

The structure was solved in the space group *P*-1 (# 2) by ShelXT 2018/2 (Sheldrick, 2018) using dual methods. It was refined by full matrix least squares minimisation on $|\boldsymbol{F}|^2$ using version of olex2.refine 1.5-dev (Bourhis et al., 2015). All non-hydrogen atoms were refined anisotropically.

Hydrogen atom positions were calculated geometrically and refined using the riding model.

_olex2_refine_details: Refinement using NoSpherA2, an implementation ofNOn-SPHERical Atom-form-factors in Olex2.Please cite:F. Kleemiss et al. Chem. Sci. DOI 10.1039/D0SC05526C - 2021NoSpherA2 implementation of HAR makes use oftailor-made aspherical atomic form factors calculatedon-the-fly from a Hirshfeld-partitioned electron density (ED) - not fromspherical-atom form factors.The ED is calculated from a gaussian basis set single determinant SCFwavefunction - either Hartree-Fock or DFT using selected funtionals- for a fragment of the crystal.This fragment can be embedded in an electrostatic crystal field by employing cluster chargesor modelled using implicit solvation models, depending on the software used.The following options were used: SOFTWARE: ORCA 6.1 PARTITIONING: NoSpherA2 INT ACCURACY: Normal METHOD: r2SCAN BASIS SET: cc-pVTZ CHARGE: 0 MULTIPLICITY: 1 DATE: 2025-12-05_15-46-53

_exptl_absorpt_process_details: CrysAlis[Pro] 1.171.44.129a (Rigaku Oxford Diffraction, 2025) using spherical harmonics,implemented in SCALE3 ABSPACK scaling algorithm.

There is a single formula unit in the asymmetric unit, which is represented by the reported sum formula. In other words: Z is 2 and Z' is 1. The moiety formula is C38 H25 N5.
